# Inhibiting Na^+^/K^+^ ATPase Can Impair Mitochondrial Energetics and Induce Abnormal Ca^2+^ Cycling and Automaticity in Guinea Pig Cardiomyocytes

**DOI:** 10.1371/journal.pone.0093928

**Published:** 2014-04-10

**Authors:** Qince Li, Steven M. Pogwizd, Sumanth D. Prabhu, Lufang Zhou

**Affiliations:** 1 Division of Cardiovascular Disease, Department of Medicine, University of Alabama at Birmingham, Birmingham, Alabama, United States of America; 2 Department of Biomedical Engineering, University of Alabama at Birmingham, Birmingham, Alabama, United States of America; 3 Comprehensive Cardiovascular Center, University of Alabama at Birmingham, Birmingham, Alabama, United States of America; Rutgers-New Jersey Medical School, United States of America

## Abstract

Cardiac glycosides have been used for the treatment of heart failure because of their capabilities of inhibiting Na^+^/K^+^ ATPase (NKA), which raises [Na^+^]_i_ and attenuates Ca^2+^ extrusion *via* the Na^+^/Ca^2+^ exchanger (NCX), causing [Ca^2+^]_i_ elevation. The resulting [Ca^2+^]_i_ accumulation further enhances Ca^2+^-induced Ca^2+^ release, generating the positive inotropic effect. However, cardiac glycosides have some toxic and side effects such as arrhythmogenesis, confining their extensive clinical applications. The mechanisms underlying the proarrhythmic effect of glycosides are not fully understood. Here we investigated the mechanisms by which glycosides could cause cardiac arrhythmias *via* impairing mitochondrial energetics using an integrative computational cardiomyocyte model. In the simulations, the effect of glycosides was mimicked by blocking NKA activity. Results showed that inhibiting NKA not only impaired mitochondrial Ca^2+^ retention (thus suppressed reactive oxygen species (ROS) scavenging) but also enhanced oxidative phosphorylation (thus increased ROS production) during the transition of increasing workload, causing oxidative stress. Moreover, concurrent blocking of mitochondrial Na^+^/Ca^2+^ exchanger, but not enhancing of Ca^2+^ uniporter, alleviated the adverse effects of NKA inhibition. Intriguingly, NKA inhibition elicited Ca^2+^ transient and action potential alternans under more stressed conditions such as severe ATP depletion, augmenting its proarrhythmic effect. This computational study provides new insights into the mechanisms underlying cardiac glycoside-induced arrhythmogenesis. The findings suggest that targeting both ion handling and mitochondria could be a very promising strategy to develop new glycoside-based therapies in the treatment of heart failure.

## Introduction

Glycosides are capable of inhibiting sarcolemmal Na^+^/K^+^-ATPase (NKA), which blocks the extrusion of Na^+^ and results in cytosolic Na^+^ accumulation [1], [2]. Elevation of Na^+^ consequently suppresses Na^+^/Ca^2+^ exchanger (NCX), the primary Ca^2+^ efflux pathway in cardiac myocytes, leading to Ca^2+^ overload and increased sarcoplasmic reticulum (SR) Ca^2+^ uptake. The resultant greater Ca^2+^-induced Ca^2+^ release (CICR) allows for more powerful contractions by cross-bridge cycling in response to stimulations [3]. Because of their positive inotropic effects, cardiac glycosides, such as digoxin, have been widely used in the treatment of congestive heart failure [2], [4]. However, recently the use of glycoside treatment in HF patients has been largely supplanted by other drugs (e.g., angiotensin-converting enzyme (ACE) inhibitors, β-blockers and aldosterone antagonists) and cardiac resynchronization therapies [5], [6]. The diminished use of glycosides in the clinic was partially due to their well-known side effects such as cardiac arrhythmias, gastrointestinal symptoms, and central nervous system abnormalities [2], [5], [7], [8]. Whereas the proarrhythmic effect of glycosides largely confines their clinic applications, the detailed underlying molecular mechanisms are not completely understood.

A classical hypothesis on the proarrhythmic effect of glycosides is that when SR Ca^2+^ stores become too high, some Ca^2+^ might be released spontaneously through ryanodine receptors (RyRs), causing early or delayed afterdepolarizations or triggered activity [1], [8], [9]. However, emerging evidence suggests that SR Ca^2+^ overload is not the only root cause of glycoside-induced cardiac arrhythmias [5]. A series of work by Dr. O’Rourke’s group has suggested that glycosides (e.g. ouabain) may impair mitochondrial energy metabolism and raise oxidative stress in guinea pig cardiomyocytes, especially those at increased workload [1], [10], [11]. Particularly, their studies showed that ouabain-induced cytosolic Na^+^ accumulation caused mitochondrial Ca^2+^ deficiency, NADH imbalance, and increased reactive oxygen species (ROS) accumulation. Since many ion channels/exchangers underlying the action potential (AP) or involved in Ca^2+^ handling (e.g., the fast Na^+^ channels, RyRs, and SR Ca^2+^ ATPase) are redox and/or ATP sensitive, ouabain-induced mitochondrial dysfunction can disturb Ca^2+^ cycling and elicit erratic action potentials. Indeed, Liu *et al.* have demonstrated that ouabain caused mitochondrial oxidative stress and DADs in guinea pig ventricular myocytes [1]. They also showed that concurrent application of CGP-37157 (a mitochondrial Na^+^/Ca^2+^ exchanger, a.k.a. mNCE, inhibitor) with ouabain retained mitochondrial Ca^2+^ and NADH levels, suppressed ROS production and prevented ouabain-induced DADs [1], [10]. These findings highlight the important roles of mitochondrial Ca^2+^ and NADH homeostasis in glycoside-induced oxidative stress and cardiac arrhythmogenesis.

In cardiac cells, mitochondrial Ca^2+^ is regulated not only by mNCE but also by mitochondrial Ca^2+^ uniporters (MCU), the primary pathway of mitochondrial Ca^2+^ uptake. While the molecular identity of MCU has been revealed recently [12], [13], the mechanisms governing MCU Ca^2+^ uptake are still incompletely understood [14]–[16]. Consequently, the role of MCU in regulating Ca^2+^ cycling and excitation-contraction (E-C) coupling remains controversial. The major restriction of mitochondrial Ca^2+^ uptake is attributed to the low affinity of MCU to Ca^2+^. Specifically, the Ca^2+^ concentration for half-maximal mitochondrial Ca^2+^ uptake *via* MCU was reported as ∼10–20 μM in isolated mitochondria [17], [18], which substantially exceeds the cytosolic Ca^2+^ concentration during E-C coupling (0.5–1.5 μM). Therefore, it has been argued that mitochondria may have a minor role in regulating cellular Ca^2+^ cycling and E-C coupling [14], [19], [20]. On the contrary, other evidence suggests that mitochondria are functionally and physically tethered to SR by a mitochondrial fusion protein (namely mitofusin 2, *a.k.a* mfn2) [21]–[23]. The proximity between mitochondria and SR [24]–[28] can form a high Ca^2+^ microdomain, facilitating dynamic interorganellar coupling (e.g. rapid, beat-to-beat MCU Ca^2+^ uptake [10], [15], [29]) and the modulation of SR Ca^2+^ release by mitochondria [30], [31]. For instance, several studies have shown that the impairment of mitochondrial function, such as dissipation of mitochondrial membrane potential or inhibition of ATP synthesis, promotes proarrhythmic Ca^2+^ alternans [32]–[34]. How glycoside-induced mitochondrial dysfunction affects Ca^2+^ handling and how the altered Ca^2+^ cycling influences energy metabolism through feedback mechanisms are not well understood and difficult to examine experimentally due to the complex and nonlinear interactions formed by the participating subcellular components.

In this study, we incorporated a detailed antioxidant system into a recently published computational model of the cardiomyocyte to quantitatively investigate the mechanisms underlying cardiac glycoside-induced mitochondrial energetic dysfunction and glycoside- mediated abnormal Ca^2+^ cycling and automaticity. Our simulation work showed that NKA inhibition caused cytosolic Na^+^ and Ca^2+^ accumulations that adversely affected mitochondrial Ca^2+^ retention and energetics (such as reduced NADH and ATP production) similar to experimental observations. In addition, the impaired mitochondrial function could be significantly alleviated by inhibiting mNCE but barely ameliorated by enhancing MCU. Finally both Ca^2+^ and AP alternans could be elicited when severe ATP depletion occurred as the result of high frequency pacing and concurrent NKA inhibition, implicating a novel mechanism underlying the arrhythmogenic effect of cardiac glycosides.

## Methods

### Model Development

**Figure 1:**
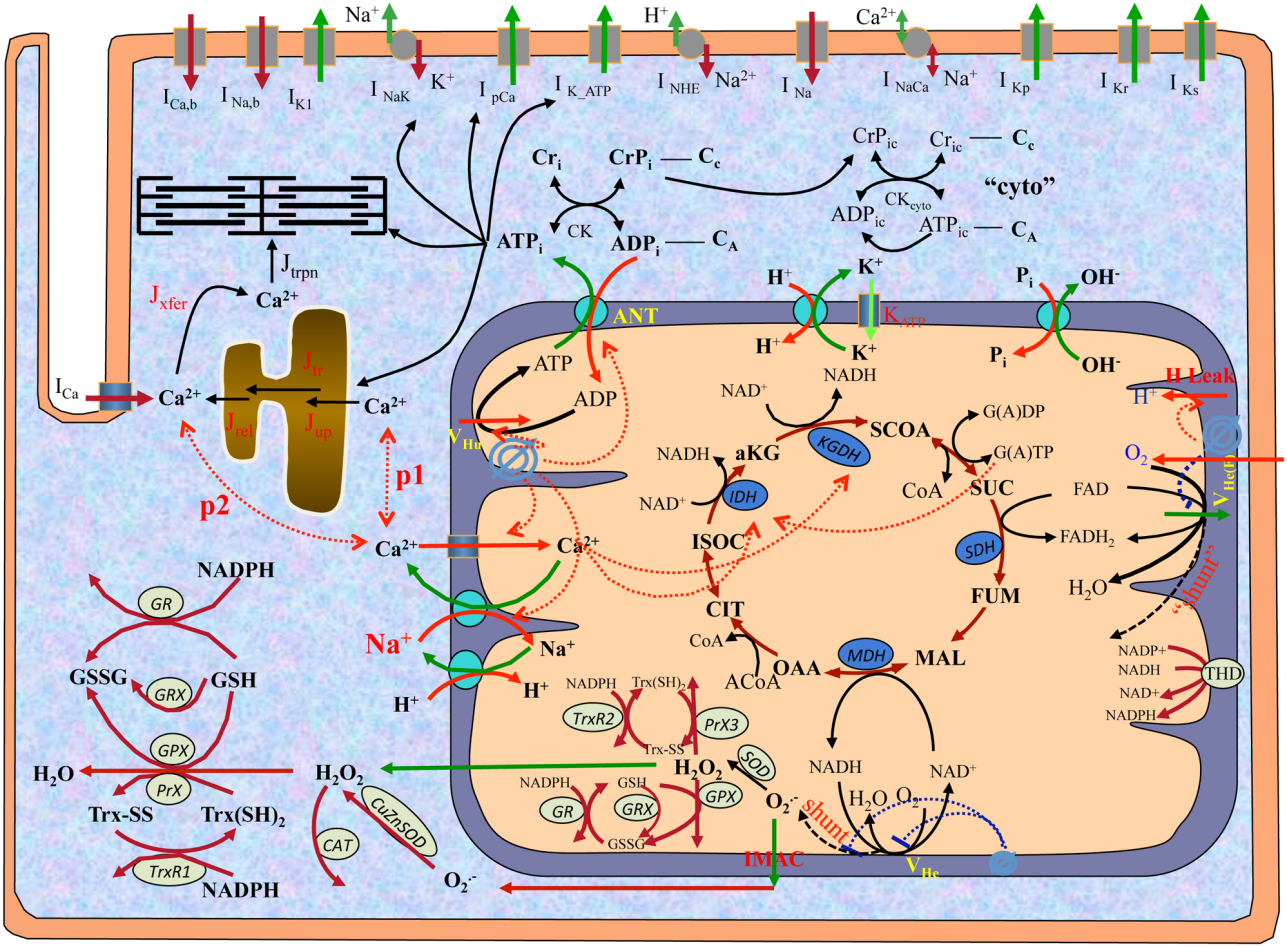
General scheme of guinea pig cardiomyocyte model. The electrophysiological module describes major ion channels underlying the action potential, E-C coupling and Ca^2+^ handling. The mitochondrial module accounts for major components of mitochondrial energetics such as TCA cycle. The ROS-induced ROS release (RIRR) module describes ROS production, transport and scavenging. The mitochondrial energetics and RIRR are linked to Ca^2+^ handling and cellular electrophysiology through signaling ions (e.g., Ca^2+^ and Na^+^) and metabolic intermediates (e.g. ATP). In the figure, *shunt* represents the fraction of ROS production from the electron transport chain, and *p1* and *p2* represent the fraction of Ca^2+^ that MCU take up from the cytosol and the mitochondria-SR microdomain, respectively.

A multiscale guinea pig cardiomyocyte model was developed in order to systematically examine the effects of glycoside-induced high cytosolic Na^+^ ([Na^+^]_i_) on the interaction between mitochondrial energetics and cellular electrophysiology. The model was based on our recently published ECME-RIRR model [35] and consisted of major ion channels/transporters underlying the action potential, E-C coupling and Ca^2+^ handling, as well as mitochondrial energetics, ROS-induced ROS release (RIRR) and intramitochondrial Ca^2+^ regulation. To replicate mitochondrial Ca^2+^ and Na^+^ handling, a refined mitochondrial model [36] consisting of Na^+^, H^+^ and P_i_^+^ channels was incorporated. Moreover, a detailed antioxidant subsystem developed by Kembro et al. [37] was modified and added to account for the regulation of mitochondrial energetics (e.g. NADH) on ROS production and scavenging. Specifically, the expanded ROS metabolism pathway is comprised of: (i) glutathione peroxidase (GPX), Mn superoxide dismutase (MnSOD), glutaredoxin (GRX), and thioredoxin (Trx) subsystems in mitochondrial matrix; (ii) GPX, catalase (CAT), and Cu,Zn superoxide dismutase (Cu,ZnSOD) subsystems in the extra-mitochondrial space; and (iii) transhydrogenase (THD) on mitochondrial inner membrane. For simplicity, the pathway of isocitrate dehydrogenase 2 (IDH2) and diffusion of reduced glutathione (GSH) in the Kembro model [37] were not considered in the current work. The general model scheme is shown in Figure 1 and the complete model equations and parameters are listed in the Supplemental Information (File S1 and S2, respectively).

### Modeling Mitochondrial Ca^2+^ Uptake through MCU

It is still debated how mitochondria take up Ca^2+^ in the cardiomyocyte. Some studies suggest that mitochondria take up Ca^2+^ solely from the cytoplasm [20], while others propose that MCU channels are tightly coupled to the SR and thus the main uptake is from the SR-mitochondria Ca^2+^ microdomain [10], [16]. We hypothesized that MCU distribute non-uniformly along the mitochondrial membrane, with expression abundant in areas facing the microdomain and scarce on membrane away from the SR. Consequently, MCU were modeled to draw Ca^2+^ from both the cytosol and the microdomain. The kinetics formulas and parameters of Ca^2+^ uptake remained the same as those in the ECME-RIRR model [35], except 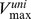 (the maximal rate of MCU) was split as 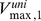
(representing uptake from the cytosol) and 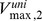 (representing uptake from the microdomain). For details see equations E168.1 and E168.2 in the Supplemental Information. The values of 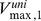
and 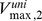
were adjusted accordingly to ensure that the total steady-state mitochondrial Ca^2+^ uptake flux equals the flux in the ECME-RIRR model: 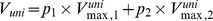, where *V_uni_*, *V_uni,1_* and *V_uni,2_* are the flux of the total Ca^2+^ uptake, the Ca^2+^ uptake from the cytosol, and the Ca^2+^ uptake from the microdomain, respectively. p1 and p2 represent the percentages of Ca^2+^ uptake flux from each pool, and their values are determined by the distribution of MCU proteins on the mitochondrial membrane.

### Model Simulation Protocol

The formulas of other processes, such as membrane potential, ion channels and metabolic reactions, and model parameters were the same as those in the ECME-RIRR model [35] (see the Supplemental Information). The code of the whole cell model was written in C^++^ (Visual Studio; Microsoft, Redmond, WA). The nonlinear ordinary differential equations were integrated numerically with CVODE as described previously [35], [38].

The cardiomyocyte was stimulated at 0.25 Hz until the steady state was reached. The steady state values were then used as initial conditions for all simulations. In the model, the effect of cardiac glycosides was mimicked by inhibiting NKA activity by 50%, which caused a 2.4-fold increase in cytosolic Na^+^ concentration in 3 mins. This amount of Na^+^ increase is comparable to that caused by 1 μM ouabain in the experimental study [1]. The cell was paced at 0.25 Hz for 1 min and then at an increasing rate (e.g., 2 Hz or 4 Hz) for 3 mins with or without NKA inhibition. We first simulated the dynamics of mitochondrial Ca^2+^ uptake, energy production and ROS metabolism during the transition of increasing workload. Various p1:p2 ratios (ranging from 1∶0 to 0∶1) were evaluated to match the experimental data [1]. The defined p1:p2 ratio was then used in the subsequent simulations. We then examined the effect of NKA inhibition-induced mitochondrial dysfunction on Ca^2+^ transients and AP under low (i.e. the fraction of electron transport chain O_2_^−^ production, *shunt*,  = 1%) and relatively high (i.e. *shunt* = 2.5%) O_2_^−^ production conditions. We also explored whether blocking mNCE or enhancing MCU alleviates NKA inhibition-induced mitochondrial energetics impairment. Finally we examined whether NKA inhibition and associated mitochondrial dysfunction are capable of triggering Ca^2+^ alternans in a paced cardiomyocyte. The simulation results were post-processed and plotted using Origin 8.6 (OriginLab, Northampton, MA).

## Results

### Effects of MCU Ca^2+^ Pools and NKA Inhibition on Intracellular Ca^2+^ Dynamics during the Transition of Increasing Workload

**Figure 2:**
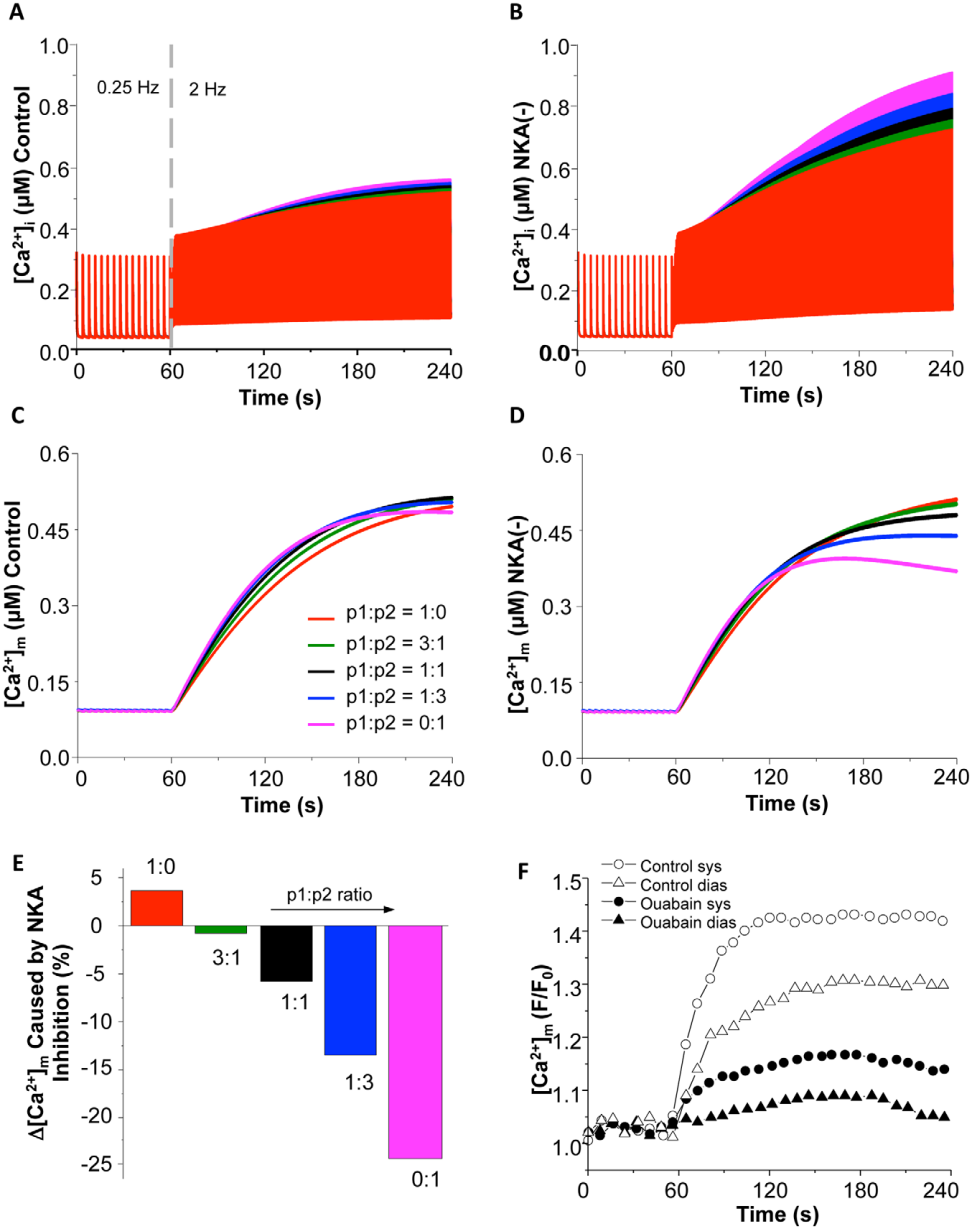
Model simulated effects of MCU Ca^2+^ uptake pool on [Ca^2+^]_i_ and [Ca^2+^]_m_ under control (A and C) and NKA inhibition (B and D) conditions during the transition of increasing pacing (from 0.25 Hz to 2 Hz). Different colors represent different p1:p2 ratios: red (1∶0); green (3∶1); black (1∶1); blue (1∶3); and pink (0∶1). (E). Summarized effect of p1:p2 ratio on [Ca^2+^]_m_ accumulation in response to increased energy demand. (F) Experimental data of the effect of NKA inhibition on [Ca^2+^]_m_ accumulation (Reproduced from [1] with permission). The pacing frequency was increased to 1 Hz at 60 s.

We first examined the impacts of different subcellular mitochondrial Ca^2+^ uptake pools (i.e. cytosol *vs.* mitochondria-SR microdomain or p1:p2 ratio) on Ca^2+^ dynamics during the transition of increasing workload with or without NKA inhibition. As expected, increasing pacing frequency (from 0.25 Hz to 2 Hz) caused significant increase in both cytosolic and mitochondrial [Ca^2+^] (Fig. 2A and 2C, respectively). Interestingly, these elevations were differentially influenced by the p1:p2 ratio. Particularly, when MCU were modeled to take up Ca^2+^ completely from the cytoplasmic pool (i.e. *p1* = 1 & *p2* = 0), [Ca^2+^]_i_ had the smallest elevation (e.g. systolic [Ca^2+^]_i_ increased by 62.5%) in response to the increasing workload (Fig. 2A). It is clear that shifting the portion of Ca^2+^ uptake from the cytosol to the microdomain (or reducing p1:p2 ratio) enhanced [Ca^2+^]_i_ accumulation. When p1 = 0 & p2 = 1 (i.e. completely from the microdomain), the increase of [Ca^2+^]_i_ was the greatest (Fig. 2A). The effect of p1:p2 ratio on mitochondrial Ca^2+^ ([Ca^2+^]_m_) was similar to that of [Ca^2+^]_i_, except when [Ca^2+^]_m_ was completely taken from the microdomain (i.e. p1 = 0 & p2 = 1), in which case [Ca^2+^]_m_ increased first and then decreased slightly (Fig. 2C).

NKA inhibition (by 50%) monotonically enhanced the accumulation of [Ca^2+^]_i_ and amplified the influence of MCU Ca^2+^ uptake pool (Fig. 2B). The effect of blocking NKA on pacing-induced [Ca^2+^]_m_ elevation was complex and dependent upon the p1:p2 ratio (Fig. 2D). As summarized in Figure 1E, NKA inhibition potentiated [Ca^2+^]_m_ accumulation when mitochondria were modeled to sequestrate all Ca^2+^ from the cytosol (i.e. p1 = 1). NKA inhibition started to suppress the pacing-induced [Ca^2+^]_m_ elevation with the pool shifting to the microdomain (i.e. reducing p1:p2 ratio). Specifically, when p1:p2 = 1∶3, NKA inhibition caused about 15% reduction in [Ca^2+^]_m_ accumulation, which was comparable to the experimental data (18% reduction in systolic [Ca^2+^]_m_ and 16% in diastolic [Ca^2+^]_m_) (Fig. 2F, reproduced from [1]). Therefore, we proposed that mitochondria take up Ca^2+^ ions mainly, if not exclusively, from mitochondrial-SR microdomain. The p1:p2 ratio of 1∶3 was used in all subsequent simulations. It is worth pointing out that in the experiments [1] the cardiomyocytes were treated with isoproterenol in addition to 1 Hz pacing. Thus, in our simulations, a higher (2 Hz) pacing frequency was used to produce comparable energy demand. In addition, the percentage of NKA activity inhibition caused by ouabain was not measured in the experiment, so the comparison was more qualitative than quantitative to some extent.

### Effect of NKA Inhibition on Intracellular Ion Homeostasis and Mitochondrial Energetics

**Figure 3:**
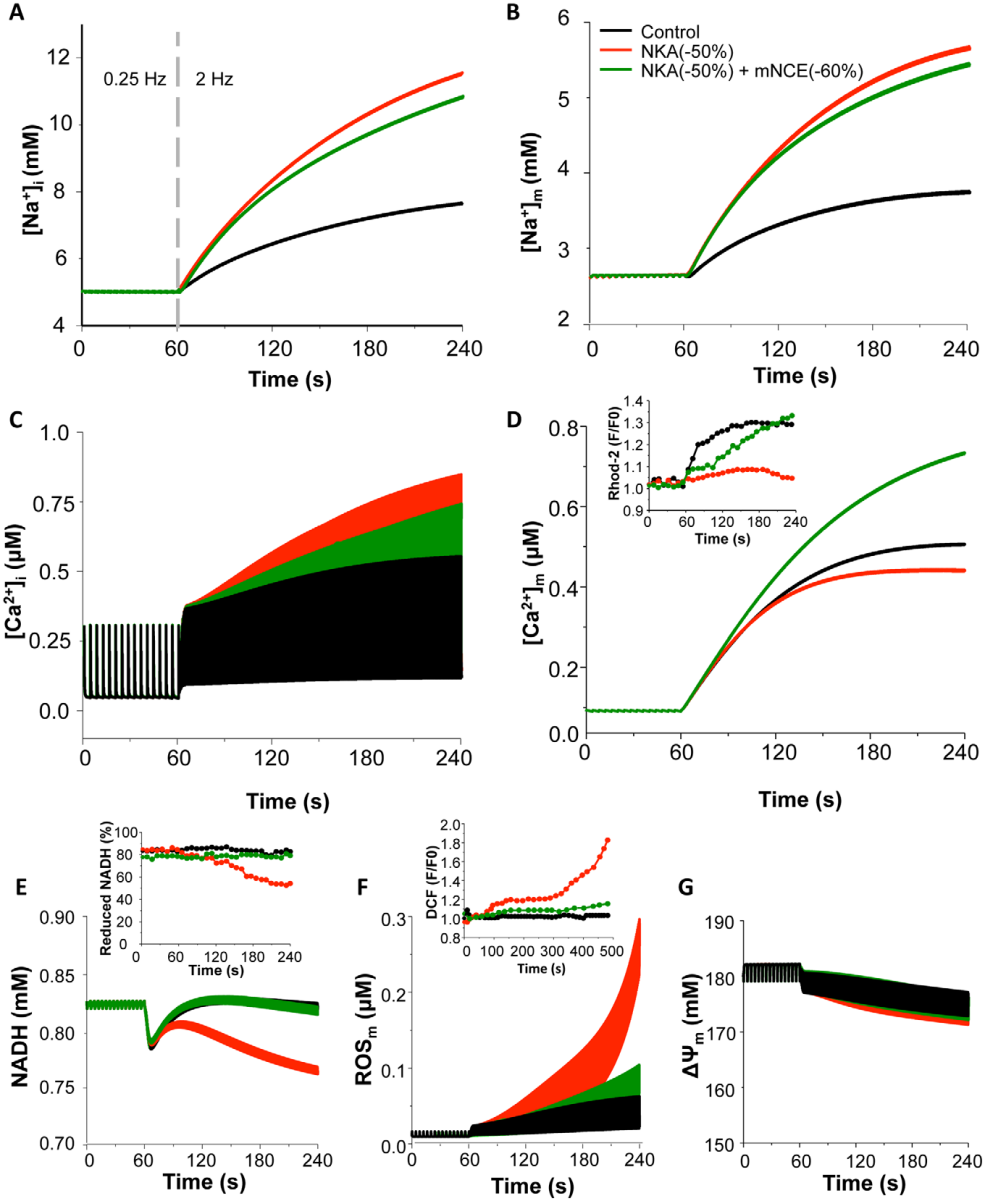
Effects of blocking NKA on ion homeostasis and mitochondrial energetics under low O_2_^−^ production conditions with or without concurrent mNCE inhibition. The cell was paced at 0.25: control; Red: 50% NKA inhibition; Dark green: 50% NKA inhibition+60% mNCE inhibition. (A): [Na^+^]_i_; (B): [Na^+^]_m_; (C): [Ca^2+^]_i_; (D): [Ca^2+^]_m_; (E): NADH; (F): ROS; and (G): mitochondrial membrane potential (ΔΨ_m_). In this simulation, *shunt* = 1% and p1:p2 = 1∶3. Inserts: Experimental data of effects of ouabain (red) and CGP-37157 (dark green) on diastolic [Ca^2+^]_m_ (Insert D), NADH (Insert E), and oxidative stress (Insert F) (Reproduced from [1]). In insert F, ouabain and CGP-37157 were applied at 1 min and pacing frequency was increased to 1 Hz at 5 min.

Next, the effects of NKA inhibition on ionic dynamics and mitochondrial energetics during the transition of increasing workload were simulated under the physiological ROS production conditions (e.g. ROS production fraction, *shunt = 1%*). Increasing pacing frequency from 0.25 Hz to 2 Hz caused a 1.6-fold elevation in [Na^+^]_i_ and a 1.5-fold increase in mitochondrial Na^+^ concentration ([Na^+^]_m_), respectively (Fig. 3A and 3B). The NADH concentration decreased transiently and then returned to the basal level (Fig. 3E), which is consistent with the experimental data when workload is acutely increased [10], [39]. Increasing pacing rate alone caused a slight increase in ROS (Fig. 3F) and had minor effects on mitochondrial membrane potential (ΔΨ_m_) (Fig. 3G), [ATP]_i_ and [ATP]_m_ (Fig. S1). Inhibiting NKA led to further increases of [Na^+^]_i_ and [Na^+^]_m_ (by 2.4-fold and 2.1-fold, respectively, Fig. 3A and 3B) and enhanced [Ca^2+^]_i_ accumulation and [Ca^2+^]_m_ attenuation (Fig. 3C and 3D). The elevation of [Na^+^]_i_ was comparable to that caused by 1 μM ouabain in the experimental study [1]. The NADH level decreased by 9% and did not return to the basal level (Fig. 3E), along with a large (∼30- fold) increase of ROS (Fig. 3F) and small loss of ΔΨ_m_ (Fig. 3G) and ATP (Fig. S1). These simulations were comparable to experimental results (Inserts in Fig. 3; reproduced from [1] with permission).

**Figure 4:**
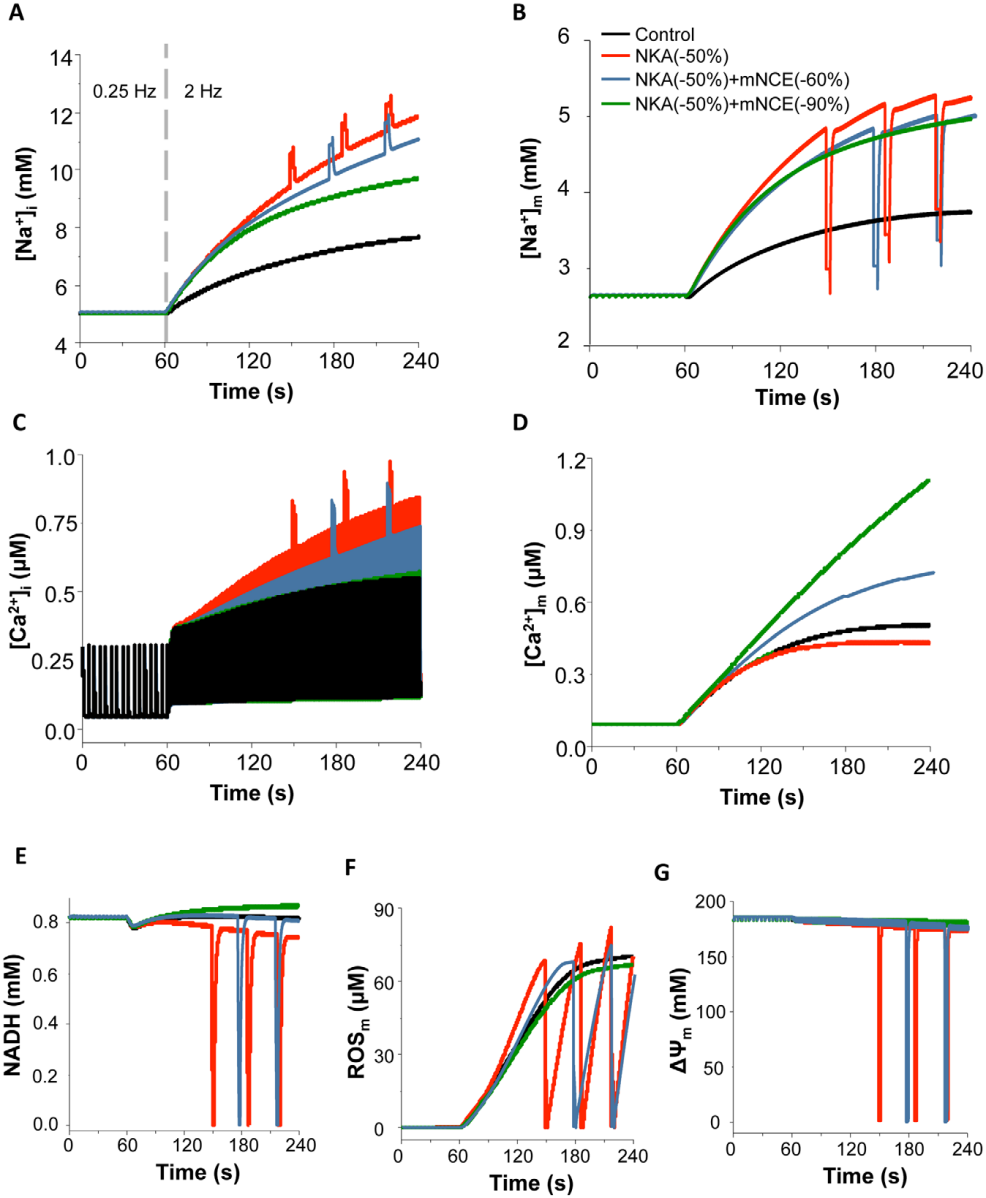
Effects of blocking NKA on ion homeostasis and mitochondrial energetics under higher O_2_^−^ production conditions with or without concurrent mNCE inhibition. Black: control; Red: 50% NKA inhibition; Blue grey: 50% NKA inhibition+60% mNCE inhibition; Dark green: 50% NKA inhibition+90% mNCE inhibition. (A): [Na^+^]_i_; (B): [Na^+^]_m_; (C): [Ca^2+^]_i_; (D): [Ca^2+^]_m_; (E): NADH; (F): ROS; and (G): mitochondrial membrane potential (ΔΨ_m_). In this simulation, *shunt* = 2.5% and p1:p2 = 1∶3.

As heart failure often associates with increased ROS production, we simulated the effect of NKA inhibition on mitochondrial energetics under a relatively higher ROS production condition (e.g. *shunt* = 2.5%). As shown in Figure 4, the small increase of *shunt* per se did not significantly alter ion concentrations and mitochondrial energetics under control condition. However, simultaneously blocking NKA (by 50%) and increasing energy demand (from 0.25 Hz to 2 Hz) caused detriment on mitochondrial energetics under the higher ROS production condition. Specifically, NKA inhibition triggered sustained mitochondrial oscillations, including NADH (Fig. 4E), ROS production (Fig. 4F) and ΔΨ_m_ (Fig. 4G). The oscillations also included Ca^2+^ and Na^+^ ions in both cytosol and mitochondria (Fig. 4A–4D).

### The Effect of Modulating mNCE or MCU on NKA Inhibition-induced Mitochondrial Dysfunction

In cardiomyocyte, mitochondrial energetics largely relies on mitochondrial Ca^2+^ retention and NADH homeostasis mediated by that Ca^2+^ retention, especially during the transition of increasing energy demand [38], [40], [41]. Therefore, we examined whether modulating mitochondrial Ca^2+^ handling channels, namely mNCE and MCU, could influence the effects of NKA inhibition on mitochondrial energetics. Our results showed that the concurrent inhibition of mNCE (e.g. by 60%) significantly ameliorated NKA inhibition-induced mitochondrial dysfunction, without abolishing the inotropic effect of NKA inhibition (Fig. 3). Specifically, NKA inhibition-induced [Na^+^]_i_ and [Na^+^]_m_ elevations were slightly attenuated and [Ca^2+^]_i_ accumulation was reduced by 13%. mNCE inhibition also overturned the blunted [Ca^2+^]_m_ retention and restored NADH concentration, as wells as suppressed ROS production and preserved ΔΨ_m_ (Fig. 3, dark green curves) and ATP (Fig. S1). The beneficial effect of mNCE inhibition was consistent with previous experimental findings that application of CGP, an mNCE inhibitor, prevented ouabain-induced mitochondrial dysfunction and resultant oxidative stress (Fig. 3 Inserts) [1], [10]. While moderate inhibition of mNCE was beneficial, too much mNCE blocking (e.g. by 90%) completely suppressed NKA inhibition-induced [Ca^2+^]_i_ elevation (and thus the inotropic effect) (data not shown). At the higher ROS production rate condition (e.g. *shunt* = 2.5%), blocking mNCE by 60% significantly delayed NKA inhibition-induced mitochondrial oscillations (Fig. 4, sky grey curves). Again, 90% mNCE inhibition counteracted the inotropic effect of glycoside, although it successfully prevented NKA inhibition-induced oxidative stress and mitochondrial oscillations (Fig. 4, dark green curves).

**Figure 5:**
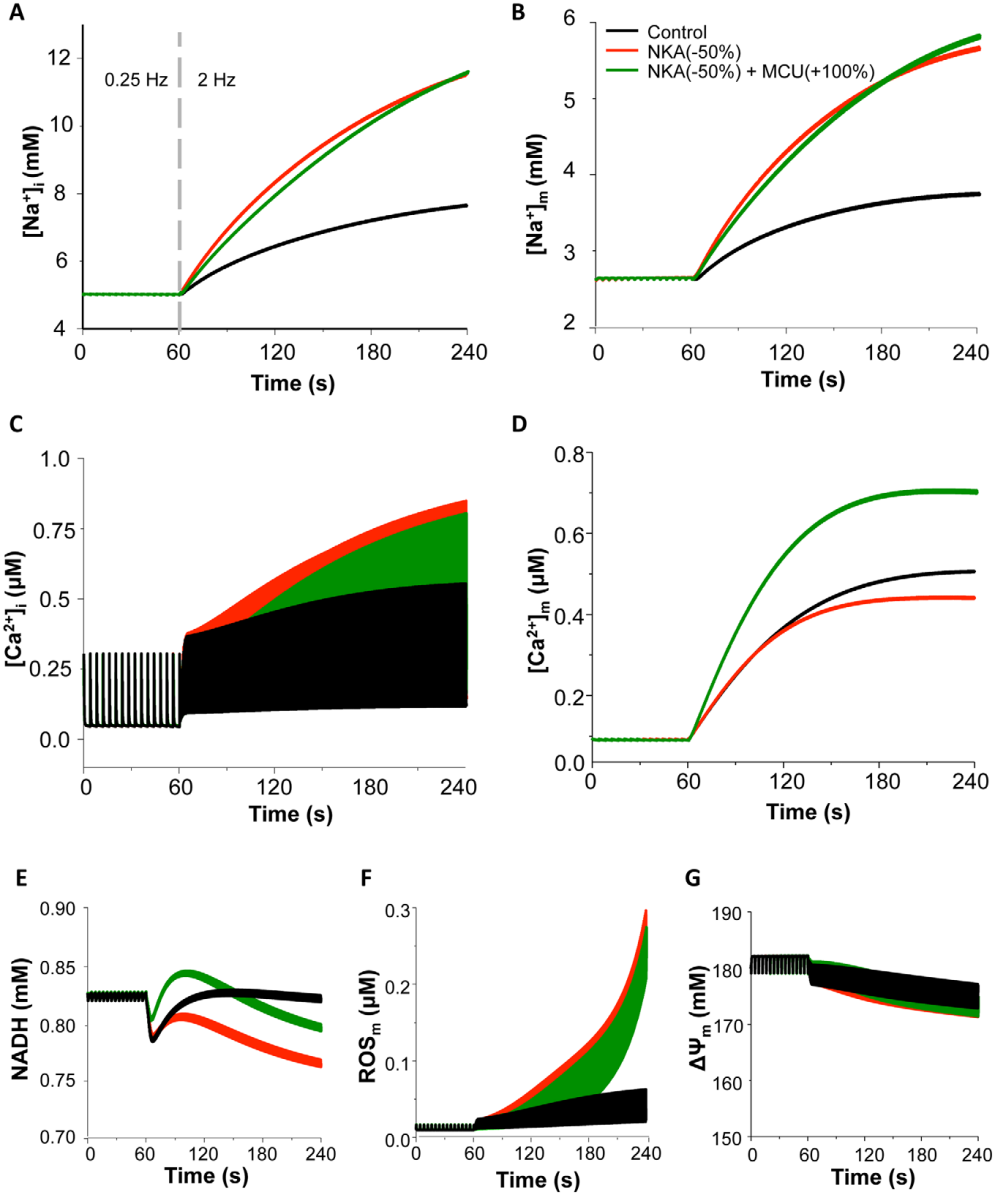
Effect of enhancing MCU on NKA inhibition-induced mitochondrial energetic dysfunction. Black: control; Red: 50% NKA inhibition; Dark green: 50% NKA inhibition+100% MCU enhancement. (A): [Na^+^]_i_; (B): [Na^+^]_m_; (C): [Ca^2+^]_i_; (D): [Ca^2+^]_m_; (E): NADH; (F): ROS; and (G): mitochondrial membrane potential (ΔΨ_m_). In this simulation, *shunt* = 1% and p1:p2 = 1∶3.

Another way to compensate NKA inhibition-induced mitochondrial Ca^2+^ decline is to increase mitochondrial Ca^2+^ uptake *via* MCU. Therefore, we examined whether enhancing MCU can alleviate NKA inhibition-induced mitochondrial energetics dysfunction similarly to mNCE inhibition. As expected, increasing MCU rate (e.g. by 100%) significantly increased [Ca^2+^]_m_ (Fig. 5D). However, its effects on NKA inhibition-induced [Na^+^]_i_, [Na^+^]_m_, and [Ca^2+^]_i_ alterations were not significant (Fig. 5A–5C). Enhancing MCU barely alleviated NKA inhibition-induced mitochondrial energetic impairments. For instance, NADH level slightly increased but did not completely recover (Fig. 5E), ROS still accumulated (Fig. 5F), and ΔΨ_m_ still dropped (Fig. 5G). Further enhancing MCU (e.g. by 4-fold) could increase mitochondrial Ca^2+^ accumulation to a level comparable to that caused by mNCE inhibition, but did not completely abolish NKA inhibition-induced mitochondrial energetics dysfunction (data not shown).

In addition to blocking mNCE and enhancing MCU, we also examined the effects of concurrently enhancing mNCE or inhibiting MCU with NKA inhibition. Increasing mNCE (100%) slightly enhanced NKA inhibition-induced [Ca^2+^]_i_ elevation (Fig. S2A) and significantly exacerbated [Ca^2+^]_m_ decline (Fig. S2B), NADH reduction (Fig. S2C) and ROS elevation (Fig. S2D). Concurrently blocking MCU (60%) with NKA inhibition had similar effects except it didn’t substantially promote ROS elevation (Fig. S2D). Further blocking MCU (100%) induced a more profound ROS elevation (data not shown).

### Effect of NKA Inhibition on Ca^2+^ Cycling and AP under more Stressed Conditions

**Figure 6:**
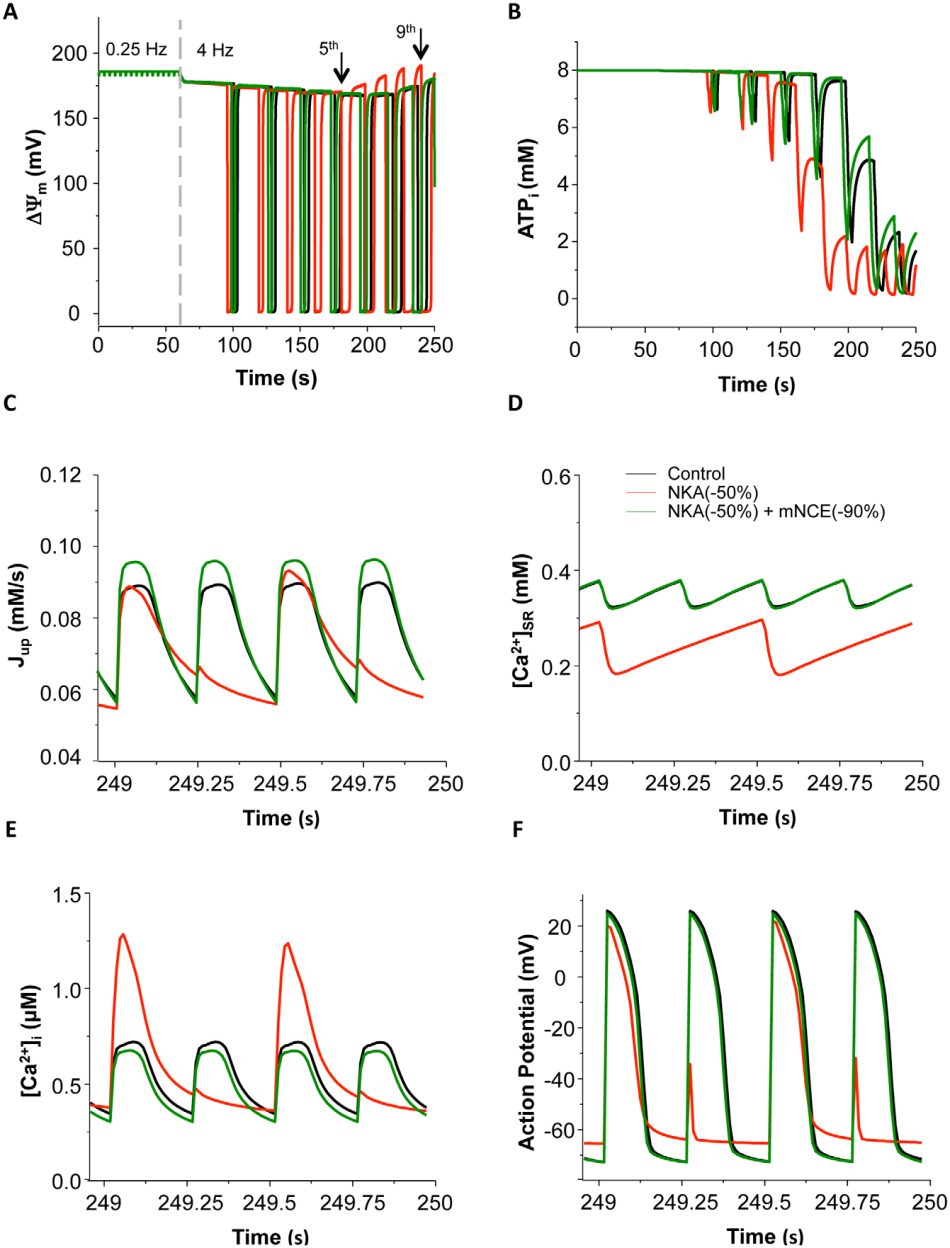
Action potential (AP) and Ca^2+^ alternans induced by NKA inhibition. The cell was paced at 0.25(by 50%). AP and Ca^2+^ alternans were observed at the end of pacing. Black: control; Red: 50% NKA inhibition; Dark green: 50% NKA inhibition+90% mNCE inhibition. (A): mitochondrial membrane potential (ΔΨ_m_); (B): ATP_i_; (C): SERCA Ca^2+^ uptake; (D): [Ca^2+^]_SR_; (E): [Ca^2+^]_i_ and (F): AP. In this simulation, *shunt* = 2.5% and p1:p2 = 1∶3.

Finally, we investigated the effect of NKA inhibition on mitochondrial energetics and cellular electrophysiology under more stressed conditions, such as more severe NKA inhibition and/or higher energy demand. Increasing pacing rate from 0.25 Hz to 4 Hz resulted in mitochondrial depolarization and oscillations when the *shunt* was set to 2.5% (Fig. 6A), as well as cyclic ATP depletions (Fig. 6B). Similar results have been reported in our previous study [35]. Concurrent induction of NKA inhibition by 50% prompted mitochondrial depolarization (Fig. 6A red curve) and accelerated ATP consumption, resulting in further decline of ATP (Fig. 6B red curve). Figures 6C–6F present the dynamics of SERCA Ca^2+^ uptake (J_up_), SR Ca^2+^ content, Ca^2+^ transients and action potential (AP) at the last second (i.e. 249 s) of (the 4 Hz) simulations. It is clear that the depletion of ATP significantly impaired SERCA Ca^2+^ uptake (Fig. 6C) and caused imbalance of SR Ca^2+^ cycling, leading to reducing and alternating SR Ca^2+^ content (Fig. 6D), as well as Ca^2+^ transient alternans with significantly increased amplitude of the larger Ca^2+^ transients within the large-small Ca^2+^ alternations (Fig. 6E). AP alternans with elevated resting potential and suppressed amplitude was also observed (Fig. 6F). More severe NKA inhibition (e.g. by 90%) accelerated ATP depletion, resulting in earlier occurrence of alternans (data not shown). Concurrent inhibition of mNCE (by 90%) retarded mitochondrial oscillations and mitigated cytosolic ATP depletion (Fig. 6A and 6B, green curve), which consequently augmented SR Ca^2+^ uptake (Fig. 6C), leading to a higher SR Ca^2+^ concentration (Fig. 6D). The stabilization and restoration of SR Ca^2+^ content abolished Ca^2+^ and AP alternans, producing a smaller Ca^2+^ transient and a larger AP amplitude (Fig. 6E and 6F).

**Figure 7:**
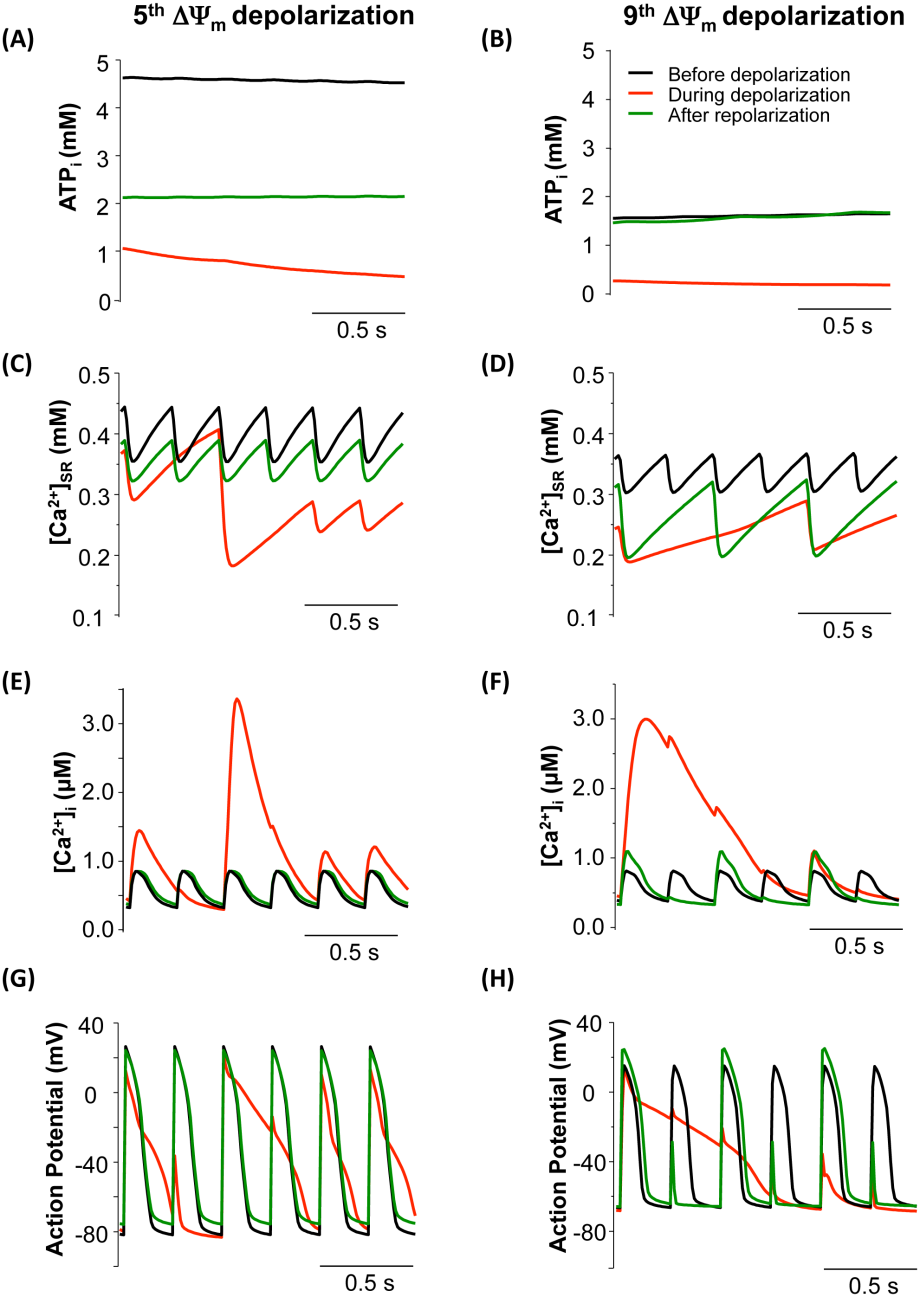
Comparisons of ATP_i_ (A and B), [Ca^2+^]_SR_ (C and D), [Ca^2+^]_i_ (E and F), and action potential (AP) (G and H) before (Black), during (Red) and after (Dark green) the 5^th^ (A,C,E,G) and 9^th^ (B,D,F,H) mitochondrial depolarization (marked with arrows in Fig. 6A). In this simulation, *shunt* = 2.5%, p1:p2 = 1∶3, and NKA inhibition was 50%.

To further explore the development of NKA inhibition-induced Ca^2+^ and AP alternans, the dynamics of ATP, [Ca^2+^]_i_, [Ca^2+^]_SR_ and AP before, during and after the 5^th^ and 9^th^ mitochondrial depolarization (indicated by arrows in Fig. 6A) were analyzed. Within the first 4 oscillations, the morphology of APs did not change significantly, although their amplitudes dropped gradually (data not shown). At the 5^th^ mitochondrial depolarization, [ATP]_i_ decreased dramatically from 4.8 mM to 2.1 mM (Fig. 7A). Consequently, SERCA Ca^2+^ uptake was significantly impaired and the SR Ca^2+^ release-uptake cycle was disturbed, resulting in a large [Ca^2+^]_SR_ depletion and [Ca^2+^]_i_ accumulation (Fig. 7C and 7E). The elevated intracellular Ca^2+^ stimulated Na^+^/Ca^2+^ exchanger and caused APD prolongation (Fig. 7G). As a result, the AP could not fully repolarize and was still in the refractory period when the next electrical stimulation fired, resulting in EAD-like abnormal APs (Fig. 7G). Nonetheless, when ΔΨ_m_ recovered and [ATP]_i_ rebounded to a relative higher level (Fig. 7A, dark green curve), the disturbed AP and Ca^2+^ transients could return to normal rhythms (Fig. 7E and 7G, dark green curves).

As mitochondrial oscillations continued, ATP (Fig. 7B) and SR Ca^2+^ content further declined (Fig. 7D), the morphologies of Ca^2+^ transients and AP became more distorted. For instance, right before the 9^th^ depolarization, [ATP]_i_ and basal [Ca^2+^]_SR_ were already dramatically low (1.6 mM and 0.36 mM, respectively) (Fig. 7B and 7D, black curves). The subsequent ΔΨ_m_ depolarization caused extra decreases of [ATP]_i_ and SR Ca^2+^ (Fig. 7B and 7D, red curves) and a much larger Ca^2+^ transient that spanned several cycle lengths (Fig. 7F, red curve). The APs also displayed erratic morphologies, with a significantly prolonged AP followed by several ones with much smaller AP amplitudes (Fig. 7H, red curve). Interestingly, the Ca^2+^ transients and AP developed alternating behaviors (i.e., alternans) rather than recovering to normal rhythm (Fig. 7F and 7H, dark green curves) when ΔΨ_m_ repolarized and ATP restored (Fig. 7B).

## Discussion

The present study provides, for the first time, a computational framework to quantitatively examine the mechanisms underlying mitochondrial dysfunction and cardiac arrhythmias induced by cardiac glycosides that are used in the treatment of heart failure. The main findings are: (i) NKA inhibition simultaneously raises [Ca^2+^]_i_ and blunts [Ca^2+^]_m_ retention, causing ATP depletion and ROS accumulation that disturb SR Ca^2+^ handling and elicit abnormal APs; (ii) NKA inhibition-induce adverse effects on mitochondrial energetics and Ca^2+^ cycling can be ameliorated by blocking mNCE, but not by enhancing MCU; (iii) under more severe conditions, NKA inhibition can cause proarrhythmic Ca^2+^ and AP alternans; and (iv) mitochondrial uniporter takes up Ca^2+^ mainly from the mitochondria-SR microdomain instead of the cytoplasm.

### Glycosides and Mitochondrial Energetics Dysfunction: New Links Revealed by Model Simulations

**Figure 8:**
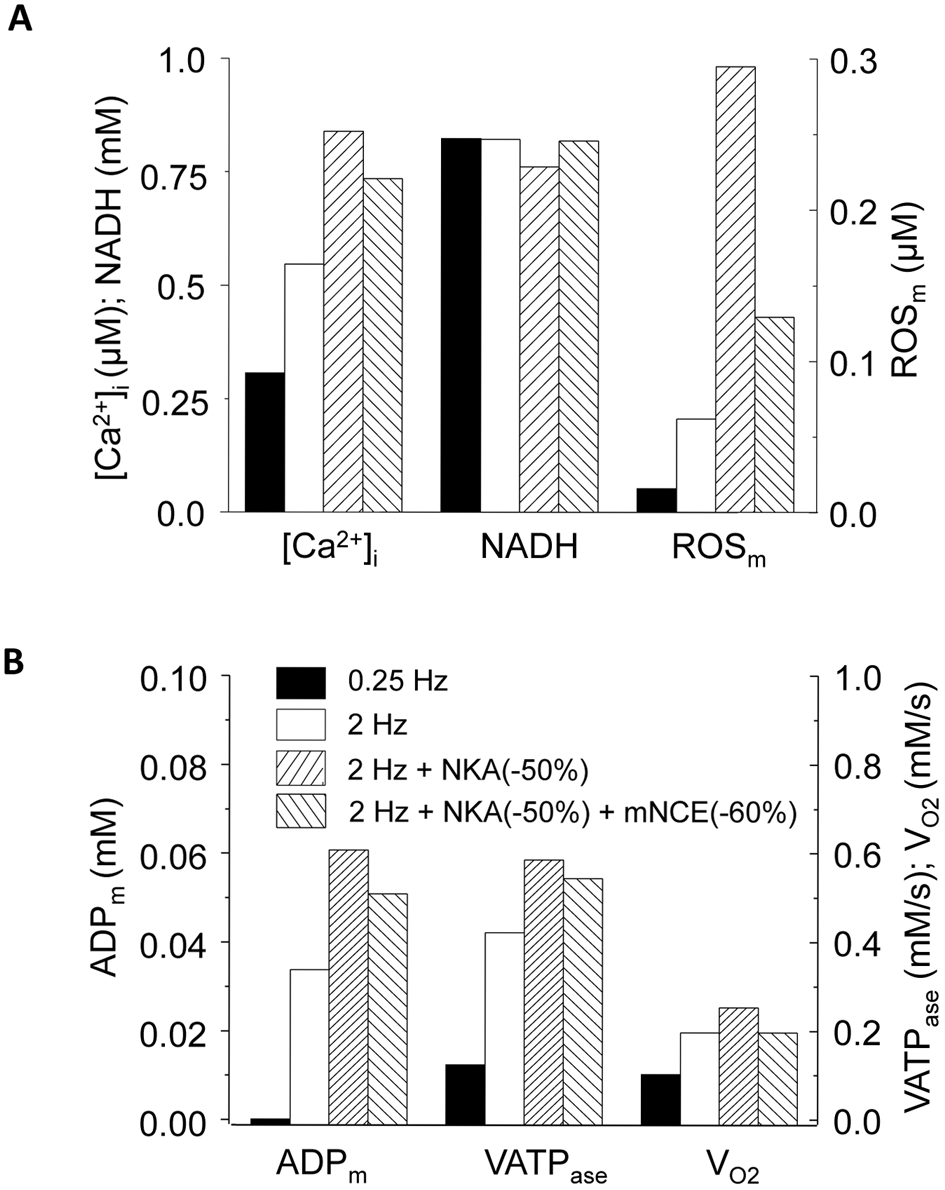
Summary of [Ca^2+^]_i_ and mitochondrial energetics under 0.25 Hz, 2 Hz, 2 Hz + NKA inhibition, and 2 Hz + NKA inhibition + mNCE inhibition conditions. (A): [Ca^2+^]_i_, NADH and ROS_m_; and (B): ADP_m_, complex V activity (V_ATPase_) and respiration rate (V_O2_). The data was recorded at the end point of simulations (i.e. 3 mins after increasing pacing). In this simulation *shunt* = 1%, p1:p2 = 1∶3, and NKA inhibition was 50%.

In cardiomyocytes, mitochondria are the major sites of ROS production, and NADH plays a vital role in regulating both the production and scavenging of ROS. Previous studies showed that inhibiting mitochondrial Ca^2+^ uptake impaired NADH production and promoted ROS accumulation [1], [10]. Our simulations were able to reproduce these experimental observations. In addition, our data demonstrated for the first time that the blunted [Ca^2+^]_m_ uptake (and resultant reduced NADH production) was not the sole reason causing oxidative stress during NKA inhibition, because ROS accumulation could occur even when NADH did not decline significantly (Fig. 8). It is known that increasing pacing frequency not only increases energy consumption but also causes ADP accumulation, which directly stimulates oxidative phosphorylation and the associated ROS production. Since NKA inhibition exacerbates [Ca^2+^]_i_ overload and enhances ATP hydrolysis, the resulting further ADP_m_ elevation can activate ATP synthesis and respiration, resulting in additional ROS production. Therefore, we propose that NKA inhibition such as by cardiac glycosides causes mitochondrial oxidative stress through two separate but concomitant pathways: (i) NKA inhibition blunting [Ca^2+^]_m_ accumulation, which reduces NADH production and therefore ROS removal, and (ii) NKA inhibition increasing [Ca^2+^]_i_ and ATP hydrolysis, which produces a large amount of ADP that stimulates mitochondrial respiration and therefore ROS production.

While the role of NKA inhibition in regulating mitochondrial ROS is well appreciated, the effect of ouabain on mitochondrial ATP synthesis remains elusive. A recent study showed that ouabain suppressed glucose-induced mitochondrial ATP production in rat pancreatic islets [42]. This inhibitory effect, caused by enhanced ROS production that impaired electron transport chain, could be observed only at high glucose concentration but not at the basal level of glucose. Similarly, another study reported that ouabain caused ATP reduction, a dramatic increase of intracellular Ca^2+^ (up to 40-fold) and enhanced ATP consumption in cultured chick ventricular cells [43]. The ATP reduction induced by ouabain was much less than that induced by cyanide and 2-deoxyglucosein, so the ATP loss observed in this experiment was likely caused by the increase of ATP consumption rather than the impairment of mitochondrial ATP synthesis. On the contrary, it has been shown that ouabain directly enhanced the rate of electron transport chain and the synthesis of ATP in cardiomyocytes *via* increasing the synthesis of ubiquinone [7]. These contradictory results might be due to differences in experimental conditions, animal species or tissue variations. Our results suggest that NKA inhibition differentially influence ATP production, depending on the severity of stress: (i) concurrent NKA inhibition and moderate increase of workload stimulates oxidative phosphorylation and ATP production, and (ii) under more severe conditions such as higher frequency pacing, NKA inhibition caused dramatic ROS accumulation, mitochondrial depolarization, and tremendous ATP depletion.

### Glycosides-induced Ca^2+^ Alternans: A New Mitochondria-originated Arrhythmic Substrate

In previous animal and clinical studies, the arrhythmogenic effects of glycosides have been mainly attributed to NKA inhibition-induced Ca^2+^ overload and associated abnormal RyR Ca^2+^ release [2], [5], [7], [8], [44]. More recently, studies by Liu *et al.* suggested that mitochondrial dysfunction, particularly ouabain-induced ROS production, might also contribute to the arrhythmogenic effect of glycosides [1]. To better understand the mechanisms underlying glycoside-induced cardiac arrhythmias, we analyzed the dynamics of Ca^2+^ in the cytosol, mitochondria and SR, as well as the profiles of metabolic intermediates such as ATP and NADH. Our results showed that NKA inhibition caused a significant ROS accumulation during the transition of moderate workload increment. The mitochondria-derived ROS may lead to abnormal Ca^2+^ handling and erratic APs, as demonstrated in [1]. More importantly, the model revealed a novel phenomenon that has not reported before: under more stressed conditions such as higher frequency pacing, NKA inhibition causes Ca^2+^ and AP alternans by reducing ATP. It is worthy to point out that although in our simulations Ca^2+^ alternans could be induced only at higher pacing rate (i.e. 4 Hz), the mechanism underlying the genesis of this type of alternans is different from that of classical alternans induced by increasing pacing rate. Actually, in our hands pacing at 4 Hz per se did not induce Ca^2+^ alternans. Further analysis showed that the genesis of Ca^2+^ alternans was attributed to NKA inhibition-induced mitochondrial dysfunction. Particularly, severe ATP depletion impaired SERCA activity, reduced the SR Ca^2+^ content, and thereby generated SR release and uptake mismatch, causing intracellular Ca^2+^ alternans. A similar mitochondrial dysfunction-induced Ca^2+^ alternans has been reported by several experimental studies [45], [46]. The role of mitochondrial dysfunction in the genesis of Ca^2+^ alternans was further supported by the mNCE inhibition simulation. As shown in Figure 5, blocking mNCE ameliorated NKA inhibition-induced mitochondrial dysfunction, mitigating the decline of SR Ca^2+^ and suppressing the genesis of alternans. Thus, our *in silico* study revealed a new plausible mechanism responsible for glycoside-induced cardiac arrhythmias, which is different from the traditional paradigm of SR Ca^2+^ overload and associated abnormal RyR Ca^2+^ release. Our model also suggested that the increase of workload could potentiate the mismatch between energy supply and demand and increase the risk of NKA inhibition-mediated arrhythmias. Thus, digitalis toxic effects could be more problematic at increased heart rate.

### Can Modulating Mitochondrial Ca^2+^ Handling Alleviate Glycoside-induced Arrhythmogenesis?

Studies have shown that CGP-17157, a mNCE inhibitor, mitigated ouabain-induced adverse effects on mitochondrial function and lowered the occurrence of cardiac arrhythmias in guinea pigs [1], [10]. The beneficial effect of CGP was thought due to its capability of retaining [Ca^2+^]_m_ accumulation that preserves mitochondrial NADH homeostasis and reduces ROS production. Our simulations are quantitatively comparable to these experimental results. Moreover, we showed that the mNCE inhibition attenuated cytosolic Ca^2+^ accumulation, which contributed to the anti-arrhythmic effect of CGP-17157. In another word, the therapeutic effect of mNCE inhibitors was attributed to not only preserving [Ca^2+^]_m_ retention and mitochondrial function, but also attenuating glycoside-induced intracellular Ca^2+^ overload. One should be aware though that it is the [Ca^2+^]_i_ elevation that is primarily responsible for the (beneficial) inotropic effect of glycosides treatment. Therefore, the dosage of the mNCE inhibitor is likely to be critical to counteracting the adverse effects of glycosides. Actually, we showed that too much mNCE inhibition, such as by 90%, completely abolished NKA inhibition-induced Ca^2+^ accumulation, although it significantly improved mitochondrial function and suppressed oxidative stress.

While inhibiting mNCE efficiently relieved NKA inhibition-induced adverse influence on mitochondrial energetics, the effect of increasing mitochondrial Ca^2+^ uniporters (MCU) activity was not evident, which might be caused by the substrate compartmentation of MCU. Our model analysis showed that MCU need to take up most of Ca^2+^ (>70%) from the microdomain in order to reproduce NKA inhibition-induced [Ca^2+^]_m_ attenuation observed in the experimental studies [1], [10]. These results indicate that the majority of Ca^2+^ taken up by MCU is from the microdomain instead of cytoplasm. Thus, enhancing MCU activity increased [Ca^2+^]_m_ uptake and retention, but it did not substantially reduce NKA inhibition-induced [Ca^2+^]_i_ overload and ROS production. These *in silico* results also imply that MCU are not uniformly distributed across the mitochondrial membrane, but rather are concentrated at mitochondria-SR contacting sites. The non-homogenous localized expression of ion channels on the sarcoplasmic or intracellular membranes has been reported previously. For instance, studies showed that L-type Ca^2+^ channels are concentrated in the neighborhood of the junctional SR at T-tubules [47], and this unique microstructure allows a correlation between ionic channel function and distribution. Similarly, abundant expression of MCU at the mitochondria-SR contacting sites can facilitate fast mitochondrial Ca^2+^ uptake in response to increased energy demand. With the molecular identity of MCU emerging [13], experimentally verifying the non-homogenous distribution of MCU in cardiac mitochondria is no longer far-fetched.

### Study Limitations

The model developed in this study incorporates subcellular Ca^2+^ compartmentation for MCU, therefore providing advantages over existing cardiomyocyte models [48]–[51]. However, it also has some limitations that need be addressed. (i) The current model lacks an isoproterenol signaling pathway, which impedes the direct comparison between model simulations and experimental data of Liu et al. [1]. However, this limitation would not alter our conclusion on the NKA inhibition-induced mitochondrial dysfunction. (ii) Previous studies have suggested that Ca^2+^ wave propagation plays a critical role in intracellular Ca^2+^ regulation and genesis of Ca^2+^ alternans [45], [46]. The “mitochondria-SR Ca^2+^ microdomain” notion proposed in this study (and by others elsewhere) may be involved in regulating Ca^2+^ wave propagation. Incorporating spatial resolution of local Ca^2+^ distribution in the microdomain would allow examination of its role in regulating cellular electrophysiology and mitochondrial energetics. (iii) The current model did not incorporate the direct link between mitochondrial ROS and redox sensitive SR Ca^2+^ proteins. It has been shown that oxidative stress can modulate SR Ca^2+^ handling proteins and cause abnormal electrical activities in cardiomyocytes [52]–[54]. The NKA inhibition-induced ROS production would exacerbate glycoside-induced abnormal cellular electrophysiology [1]. Adding mdROS modulation of redox sensitive ion channels/transporters is ongoing in our laboratory. (iv) In the current model, the equations of complexes of the electron transport chain are lumped, which makes it hard to investigate the specific role of the complexes of electron transport chain (ETC) in mitochondrial dysfunction. A more detailed ETC model [55] can be incorporated into the ECME-RIRR model to examine the effect of ETC complexes on mitochondrial function. (v) The present model did not consider the effect of energy depletion on ATP-sensitive potassium channels (K_ATP_), as our main focus is on mitochondrial dysfunction and Ca^2+^ handling. It is expected that incorporating K_ATP_ would exacerbate the deleterious effect of NKA inhibition-induced mitochondrial dysfunction on cardiac action potentials. As shown before, depletion of ATP activates K_ATP_ currents, resulting in AP shortening or even cellular inexcitability [35], [56].

In summary, the present study provides a novel computational tool to quantitatively investigate the proarrhythmic effects of glycosides (and resultant NKA inhibition) in the cardiomyocyte. The simulations reveal that NKA inhibition can cause mitochondrial oxidative stress through two separate and concomitant pathways, which involve an intricate interplay between cardiac electrophysiology and mitochondrial energetics. Moreover, the model suggests that under more severe conditions, NKA inhibition can induce Ca^2+^ alternans, contributing to its proarrhythmic effect. Finally, the study verifies the role of mitochondria-SR Ca^2+^ microdomain in regulating mitochondrial Ca^2+^ uptake and energetics. Taken together, this work underscores the importance of targeting both intracellular ion handling and mitochondrial energetics in developing new glycoside-based heart failure therapies.

## Supporting Information

Figure S1 **Effects of blocking NKA on cytosolic and mitochondrial ATP under low O_2_^−^ production conditions with or without concurrent mNCE inhibition.** The cell was paced at 0.25 Hz for 1 min then at 2 Hz for 3 mins. Black: control; Red: 50% NKA inhibition; Dark green: 50% NKA inhibition+60% mNCE inhibition. (A): [ATP]_i_; (B): [ATP]_m_. *shunt* = 1% and p1:p2 = 1∶3.
(TIF)

Figure S2 **Effect of blocking MCU or enhancing mNCE on NKA inhibition-induced Ca^2+^ and mitochondrial energetics changes.** Black: control; Red: 50% NKA inhibition; Dark green: 50% NKA inhibition+60% MCU inhibition; Blue: 50% NKA inhibition+100% mNCE enhancement. (A): [Ca^2+^]_i_; (B): [Ca^2+^]_m_; (C): NADH; (D): ROS. *shunt* = 1% and p1:p2 = 1∶3.
(TIF)

File S1 **Supplemental Information model equations.**
(DOCX)

File S2 **Supplemental Information model parameters.**
(DOCX)
