# Supplementary figures and images for: Inhibiting Na+/K+ ATPase Can Impair Mitochondrial Energetics and Induce Abnormal Ca2+ Cycling and Automaticity in Guinea Pig Cardiomyocytes
Source: PLoS One. 2014 Apr 10;9(4):e93928. doi: 10.1371/journal.pone.0093928 (PMC3983106; doi:10.1371/journal.pone.0093928)

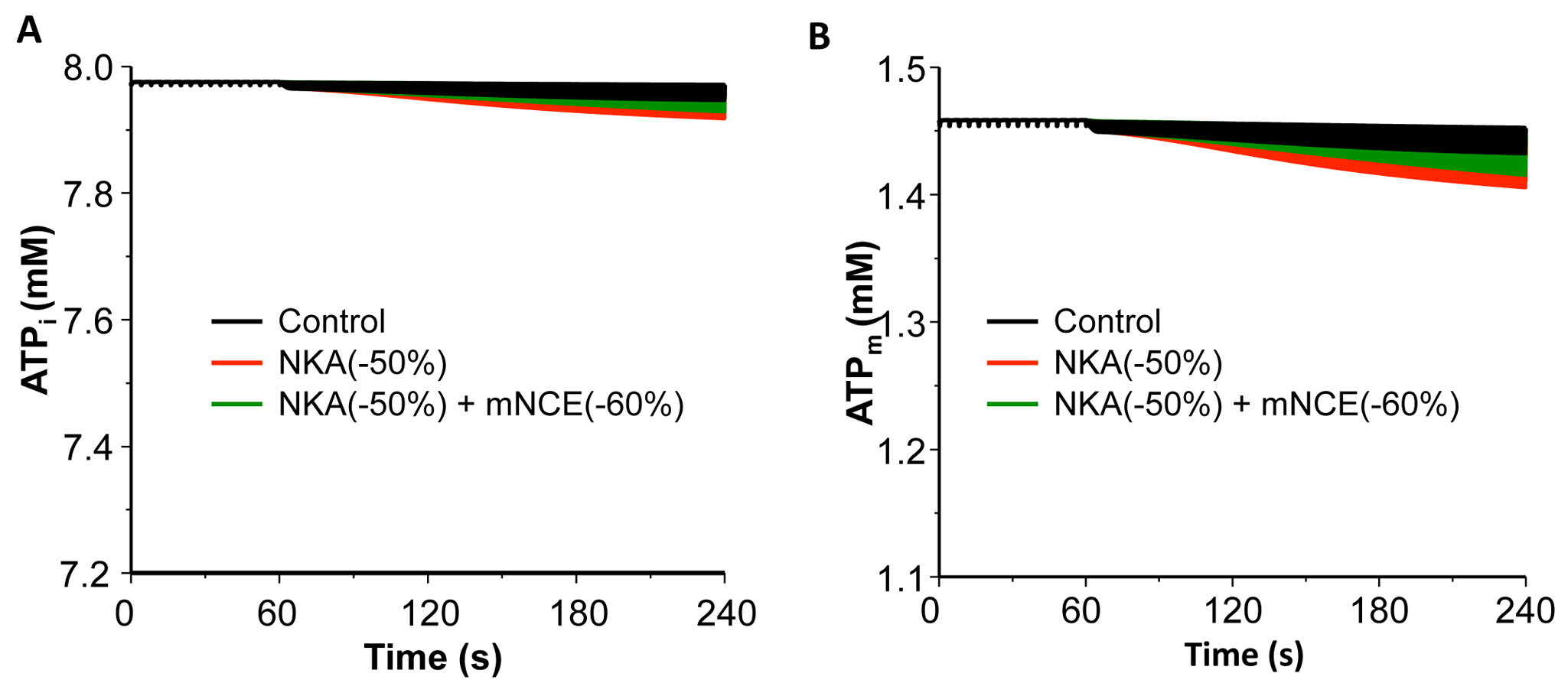

Supplement: Figure S1 — Effects of blocking NKA on cytosolic and mitochondrial ATP under low O2− production conditions with or without concurrent mNCE inhibition. The cell was paced at 0.25 Hz for 1 min then at 2 Hz for 3 mins. Black: control; Red: 50% NKA inhibition; Dark green: 50% NKA inhibition+60% mNCE inhibition. (A): [ATP]i; (B): [ATP]m. shunt = 1% and p1:p2 = 1∶3. (TIF) [file pone.0093928.s001.tif]

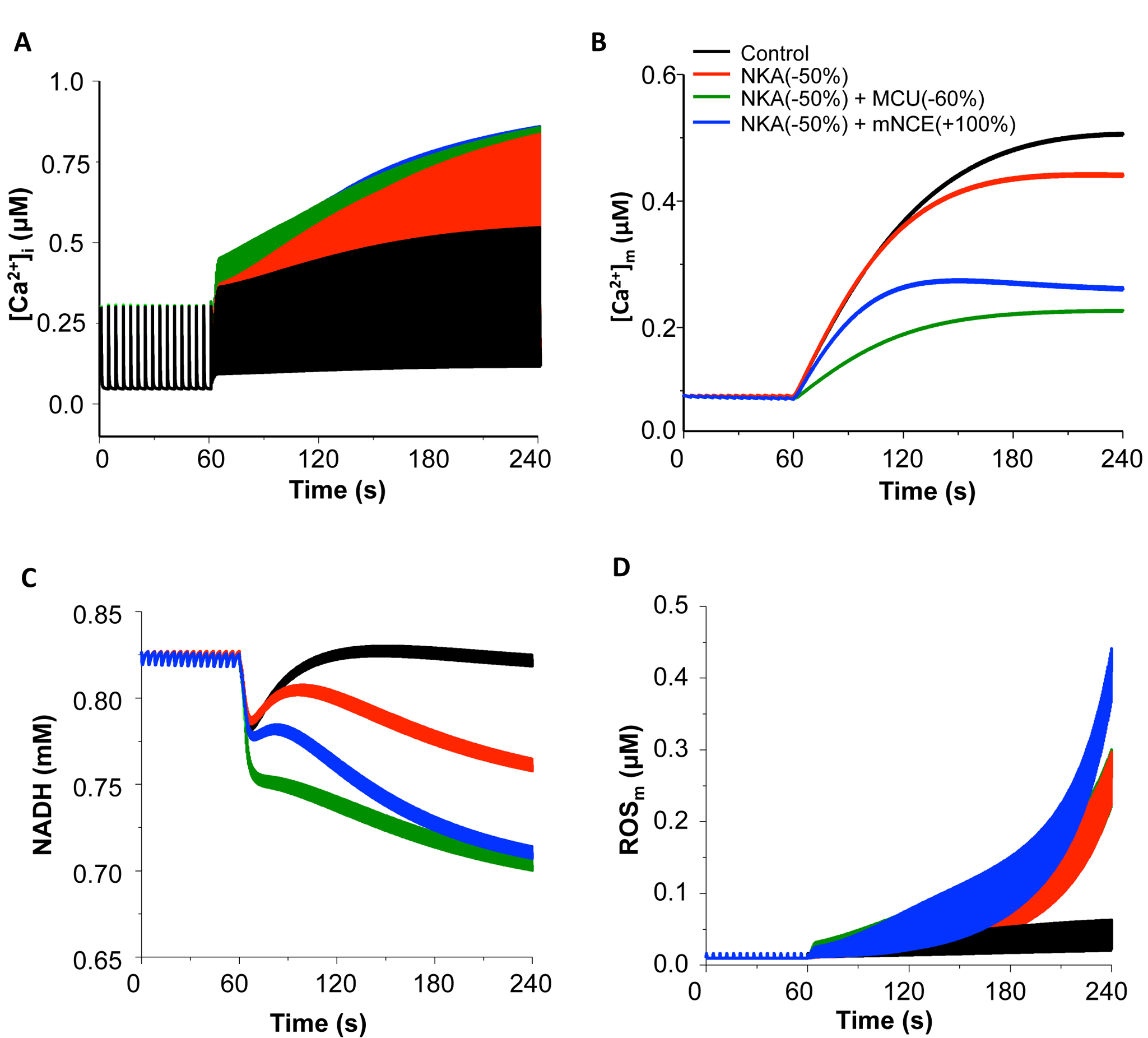

Supplement: Figure S2 — Effect of blocking MCU or enhancing mNCE on NKA inhibition-induced Ca2+ and mitochondrial energetics changes. Black: control; Red: 50% NKA inhibition; Dark green: 50% NKA inhibition+60% MCU inhibition; Blue: 50% NKA inhibition+100% mNCE enhancement. (A): [Ca2+]i; (B): [Ca2+]m; (C): NADH; (D): ROS. shunt = 1% and p1:p2 = 1∶3. (TIF) [file pone.0093928.s002.tif]
