# Supplementary material for: Inhibiting Na+/K+ ATPase Can Impair Mitochondrial Energetics and Induce Abnormal Ca2+ Cycling and Automaticity in Guinea Pig Cardiomyocytes
Source: PLoS One. 2014 Apr 10;9(4):e93928. doi: 10.1371/journal.pone.0093928 (PMC3983106; doi:10.1371/journal.pone.0093928)
Supplement: File S1 — Supplemental Information model equations. (DOCX) [file pone.0093928.s003.docx]

### S1. Supplemental Information model equations

The model is developed based on our recently published ECME-RIRR (27). Briefly, the following modifications/improvements have been made: (i) mitochondrial Ca^2+^ uptake was split into two components: the Ca^2+^ uptake from the cytosol V_uni1_ and the Ca^2+^ uptake from the microdomain V_uni2_; (ii) a detailed antioxidant subsystem developed by Kembro et al. (1) was modified and incorporated into the ECME-RIRR model. For simplicity, the pathway of IDH2 and GSH diffusion in the Kembro model (1) were not considered; (iii) a parameter of Pi buffer capacity was integrated to maintain mitochondrial Pi at mM level; and (iv) mitochondrial K^+^-H^+^ exchanger was integrated in to better replicate mitochondrial ion handling. The detailed model equations and parameters are listed below and S2, respectively.

### S1.1 Sarcolemmal membrane ionic currents

#### Fast Na^+^ current (I_Na_)

| $I_{Na}\text{ }=\text{ }\overline{\text{G}}_{\text{Na}}\text{ m}^{3}\text{ h j }\left(\text{V - E}_{\text{Na}}\right)$ | E1 |
| --- | --- |
| $E_{Na}\text{ }=\text{ }\frac{\text{RT}}{\text{F}}\text{ ln }\left(\frac{\text{[Na}^{+}\text{]}_{\text{o}}}{\text{[Na}^{+}\text{]}_{\text{i}}}\right)$ | E2 |
| $\frac{dm_{Na}}{dt}\text{ }=\text{ }\alpha_{\text{m}}\left(\text{1-}m_{Na}\right)\text{ - }\beta_{\text{m}}m_{Na}$ | E3 |
| $\frac{dh_{Na}}{dt}\text{ }=\text{ }\alpha_{\text{h}}\left(\text{1-}h_{Na}\right)\text{ - }\beta_{\text{h}}h_{Na}$ | E4 |
| $\frac{dj_{Na}}{dt}\text{ }=\text{ }\alpha_{\text{j}}\left(\text{1-}j_{Na}\right)\text{ - }\beta_{\text{j}}j_{Na}$ | E5 |
| $\alpha_{m}=\text{ 0.32 }\frac{\text{V }+\text{ 47.13}}{\text{1 - e }^{\text{-0.1 }\left(\text{V}+47.13\right)}}$ | E6 |
| $\beta_{m}\text{ }=\text{ 0.08 e}^{\text{-V/11}}$ | E7 |
| For V ≥ -40 mV |  |
| $\alpha_{h}\text{ }=\text{ 0.0}$ | E8 |
| $\alpha_{j}\text{ }=\text{ 0.0}$ | E9 |
| $\beta_{h}\text{ }=\text{ }\left(\text{0.13 }\left(\text{1 }+\text{ e }^{\left(\frac{\text{V}+10.66}{-11.1}\right)}\right)\right)^{-1}$ | E10 |
| $\beta_{j}\text{ }=\text{ 0.3 }\frac{\text{e }^{\text{-2.535 }\times\text{ 10}^{\text{-7}}V}}{\text{1 }+\text{ e }^{\text{-0.1 }\left(\text{V}+32\right)}}$ | E11 |
| For V< -40 mV |  |
| $\alpha_{h}\text{ }=\text{ 0.135 e}^{\frac{\text{80 }+\text{ V}}{\text{- 6.8}}}$ | E12 |
| $\alpha_{j}\text{ }=\text{ }\frac{\left(\text{-127,140 e}^{0.2444\text{ }V}\text{ -3.474 }\times\text{ 10}^{\text{-5}}\text{ e}^{-0.04\text{391 }V}\right)\text{ }\times\text{(V }+\text{ 37.78)}}{\text{1 }+\text{ e }^{\text{0.311 (V}+\text{79.23)}}}$ | E13 |
| $\beta_{h}\text{ }=\text{ 3.56 e}^{\text{ 0.079 V}}\text{ }+\text{ }3\text{.1 }\times\text{ 10}^{5}\text{ e }^{\text{0.35 V}}$ | E14 |
| $\beta_{j}\text{ }=\text{ 0.1212 }\frac{\text{e }^{\text{-}0\text{.01052 }V}}{\text{1 }+\text{ e }^{\text{-0.1378 }\left(\text{V}+\text{ 40.14}\right)}}$ | E15 |

#### Time-dependent delayed rectifier K^+^ current (I_K_)

| $I_{K}\text{ }=\text{ }\overline{G}_{K}\text{ }X_{1}\text{ }X^{2}\left(V\text{ }-\text{ }E_{K}\right)$ | E16 |
| --- | --- |
| $E_{K}\text{ }=\text{ }\frac{\text{RT}}{\text{F}}\text{ ln }\left(\frac{\text{[K}^{+}\text{]}_{\text{o}}\text{ }+\text{ P}_{\text{Na,K}}\text{ [Na}^{+}\text{]}_{\text{o}}}{\text{[K}^{+}\text{]}_{\text{i}}\text{ }+\text{ P}_{\text{Na,K}}\text{ [Na}^{+}\text{]}_{\text{i}}}\right)$ | E17 |
| $\overline{G}_{K}\text{ }=\text{ 0.282 }\sqrt{\frac{\text{[K}^{+}\text{]}_{\text{o}}}{5.4}}$ | E18 |
| $X_{1}\text{ }=\text{ }\left(\text{1 }+\text{ e }^{\left(\text{V- 40}\right)/40}\right)^{-1}$ | E19 |
| $\frac{dX}{dt}\text{ = }\alpha_{\chi}\text{ }\left(\text{1 - X}\right)\text{ - }\beta_{\chi}\text{ X}$ | E20 |
| $\alpha_{\chi}\text{ = 7.19 10}^{\text{-5}}\text{ }\frac{\text{V + 30}}{\text{1 - }e^{-0.148\left(V+30\right)}}$ | E21 |
| $\beta_{\chi}\text{ = 1.31 10}^{\text{-4}}\text{ }\frac{\text{V + 30}}{\text{-1 + }e^{0.0687 \left(V+30\right)}}$ | E22 |

#### Time-independent K^+^ current (I_K1_)

| $I_{K_{1}}\text{ }=\text{ }\overline{G}_{\text{K}_{1}}\text{ }K_{1\infty}\left(V\text{ }-\text{ }E_{K_{1}}\right)$ | E23 |
| --- | --- |
| $E_{K_{1}}\text{ }=\text{ }\frac{\text{RT}}{\text{F}}\text{ ln }\left(\frac{\text{[K}^{+}\text{]}_{\text{o}}}{\text{[K}^{+}\text{]}_{\text{i}}}\right)$ | E24 |
| $\overline{G}_{K_{1}}\text{ }=\text{ 0.75 }\sqrt{\frac{\text{[K}^{+}\text{]}_{\text{o}}}{5.4}}$ | E25 |
| $K_{1\infty}\text{ }=\text{ }\frac{\alpha_{\text{K}_{1}}}{\alpha_{\text{K}_{1}}\text{ }+\text{ }\beta_{K_{1}}}$ | E26 |
| $\alpha_{K_{1}}\text{ = }\frac{1.02}{\text{1 + }e^{0.2385\left(V-E_{K_{1}}-59.215\right)}}$ | E27 |
| $\beta_{K_{1}}\text{ = }\frac{0.4912 e^{0.08032\left(V-E_{K_{1}}+5.476\right)}\text{ + }e^{0.06175\left(V-E_{K_{1}}-594.31\right)}}{\text{1 + }e^{- 0.5143\left(V-E_{K_{1}}+4.753\right)}}$ | E28 |

#### Plateau K^+^ current (I_Kp_)

| $I_{K_{P}}\text{ }=\text{ }\overline{G}_{K_{P}}\text{ }K_{P}\left(V\text{ }-\text{ }E_{K_{P}}\right)$ | E29 |
| --- | --- |
| $E_{K_{P}}\text{ }=\text{ }E_{K_{1}}$ | E30 |
| $K_{p}\text{ = }\left(\text{1 + }e^{\left(7.488-V\right)/5.98}\right)^{-1}$ | E31 |

#### Na^+^/Ca^2+^ exchanger current (I_NaCa_)

| $\begin{matrix} I_{NaCa}\text{ = k}_{\text{NaCa}}\text{ }\frac{1}{\text{K}_{\text{m,Na}}^{3}+[Na^{+}]_{o}^{3}}\text{ }\frac{1}{\text{K}_{\text{m,Ca}}+[Ca^{2+}]_{o}}\left(1+k_{sat} e^{\left(\eta-1\right)\frac{VF}{RT}}\right)^{-1} \\ \left\{e^{\eta\frac{VF}{RT}}\text{ [Na}^{\text{+}}\text{]}_{\text{i}}^{3}\text{ [Ca}^{\text{2+}}\text{]}_{\text{o}}-\text{ }e^{\left(\eta-1\right)\frac{VF}{RT}}\text{ [Na}^{\text{+}}\text{]}_{\text{o}}^{3}\text{ [Ca}^{\text{2+}}\text{]}_{\text{i}}\right\} \end{matrix}$ | E32 |
| --- | --- |

#### Na^+^/ K^+^ pump current (I_NaK_)

| $I_{NaK}\text{ = }\overline{\text{I}}_{\text{NaK}}\text{ f}_{\text{NaK}}\text{ f}_{\text{NaK}}^{\text{ATP}} \left(\text{1 + }\left(\frac{\text{K}_{\text{m,Na}_{\text{i}}}}{\text{[Na}^{\text{+}}\text{]}_{\text{i}}}\right)^{1.5}\right)^{-1}\text{ }\frac{\text{[K}^{\text{+}}\text{]}_{\text{o}}}{\text{[K}^{\text{+}}\text{]}_{\text{o}}\text{ + K}_{\text{m,K}_{\text{o}}}}$ | E33 |
| --- | --- |
| $f_{NaK}\text{ = }\left(\text{1 + 0.1245 }e^{-0.1\text{ }\frac{\text{VF}}{\text{RT}}}\text{ + 0.0365 }e^{- \frac{\text{VF}}{\text{RT}}}\left(\frac{e^{[Na^{+}]_{o}/67.3}-1}{7}\right)\right)^{-1}$ | E34 |
| $f_{NaK}^{ATP}\text{ = }\left(\text{1+ }\frac{\text{K}_{\text{NaK}}^{\text{1, ATP}}}{[ATP]_{i}}\left(1\text{ + }\frac{\text{[ADP]}_{\text{i}}}{\text{K}_{\text{NaK}}^{\text{i, ADP}}}\right)\right)^{-1}$ | E35 |

#### Nonspecific Ca^2+^ activated current (I_nsCa_)

| $I_{nsCa}\text{ = I}_{\text{nsNa}}\text{ + I}_{\text{nsK}}$ | E36 |
| --- | --- |
| $I_{nsNa}=\overline{I}_{nsNa}\text{ }\left(\text{1 + }\left[\frac{\text{K}_{\text{m,nsCa}}}{\text{[Ca}^{\text{2+}}\text{]}_{\text{i}}}\right]^{3}\right)^{-1}$ | E37 |
| $\overline{I}_{nsNa}\text{ = P}_{\text{nsNa}}\text{ }\frac{\text{VF}^{2}}{\text{RT}}\text{ }\frac{\text{0.75 }\left(\text{[Na}^{\text{+}}\text{]}_{\text{i}}\text{ e}^{\text{VF/RT}}\text{ - [Na}^{\text{+}}\text{]}_{\text{o}}\right)}{\text{e}^{\text{VF/RT}}\text{ - 1}}$ | E38 |
| $I_{nsK}=\overline{I}_{nsK}\text{ }\left(\text{1 + }\left[\frac{\text{K}_{\text{m,nsCa}}}{\text{[Ca}^{\text{2+}}\text{]}_{\text{i}}}\right]^{3}\right)^{-1}$ | E39 |
| $\overline{I}_{nsK}\text{ = P}_{\text{nsK}}\text{ }\frac{\text{VF}^{2}}{\text{RT}}\text{ }\frac{\text{0.75 }\left(\text{[K}^{\text{+}}\text{]}_{\text{i}}\text{ e}^{\text{VF/RT}}\text{ - [K}^{\text{+}}\text{]}_{\text{o}}\right)}{\text{e}^{\text{VF/RT}}\text{ - 1}}$ | E40 |

#### Background Ca^2+^ current (I_Ca,b_)

| $I_{Ca,b}\text{ = }\overline{\text{G}}_{\text{Ca,b}}\text{ }\left(\text{V - E}_{\text{Ca,N}}\right)$ | E41 |
| --- | --- |
| $E_{Ca,N}\text{ = }\frac{\text{RT}}{\text{2F}}\text{ ln }\left(\frac{\text{[Ca}^{\text{2+}}\text{]}_{\text{o}}}{\text{[Ca}^{\text{2+}}\text{]}_{\text{i}}}\right)$ | E42 |

#### Background Na^+^ current (I_Na,b_)

| $I_{Na.b}\text{ = }\overline{\text{G}}_{\text{Na,b}}\text{ }\left(\text{V - E}_{\text{Na,N}}\right)$ | E43 |
| --- | --- |
| $E_{Na,N}\text{ = E}_{\text{Na}}$ | E44 |

#### Sarcolemmal Ca^2+^ pump current (I_pCa_)

| $\text{I}_{\text{pCa}}=\text{I}_{\text{pCa\_max}} F_{pCa}^{ATP}\frac{\text{[Ca}^{\text{+2}}\text{]}_{\text{i}}}{K_{m}^{pCa}+\text{[Ca}^{\text{+2}}\text{]}_{\text{i}}}$ | E45 |
| --- | --- |
| $\text{F}_{\text{pCa}}^{ATP}=\left(1+\frac{K_{m1\_pCa}^{ATP}}{[ATP]_{i}}\left(1+\frac{[ADP]_{i}}{K_{i\_pCa}^{ADP}}\right)\right)^{-1}+ \left(1+\frac{K_{m2\_pCa}^{ATP}}{[ATP]_{i}}\right)^{-1}$ | E46 |

#### SERCA pump (J_up_)

| $J_{up}=\text{ }\frac{V_{\max f} f_{b} - V_{\max r} r_{b} }{1 + f_{b} + r_{b}}\text{ }f_{ATP}^{SERCA}$ | E47 |
| --- | --- |
| $f_{b}\text{ }=\text{ }\left(\frac{[Ca^{2+}]_{i}}{K_{fb}}\right)^{Nfb}$ | E48 |
| $r_{b}\text{ }=\text{ }\left(\frac{[Ca^{2+}]_{NSR}}{K_{rb}}\right)^{Nrb}$ | E49 |
| $f_{ATP}^{SERCA}\text{ = }\left(\frac{\text{K}_{\text{m,up}}^{\text{ATP}}}{[ATP]_{i}}.\left(1+\frac{[ADP]_{i}}{K_{i,up}}\right)+\text{ }\left[1\text{ }+\text{ }\frac{[ADP]_{i}}{K_{i,up}^{'}}\right]\right)^{-1}$ | E50 |

#### L-type Ca^2+^ current (I_Ca_)

| $\alpha\text{ = 0.4 e}^{\text{(V+2)/10}}$ | E51 |
| --- | --- |
| $\beta\text{ = 0.05 e}^{\text{-(V+2)/13}}$ | E52 |
| $\alpha'\text{ = a }\alpha$ | E53 |
| $\beta'\text{ = }\frac{\beta}{\text{b}}$ | E54 |
| $\gamma\text{ = 0.1875 [Ca}^{\text{2+}}\text{]}_{\text{ss}}$ | E55 |
| $\frac{dC_{o}}{dt}\text{ = }\beta\text{ C}_{1}\text{ + }\omega\text{ C}_{\text{CaO}}\text{ - }\left(4\alpha\text{ + }\gamma\right)Co$ | E56 |
| $\frac{dC_{1}}{dt}\text{ = 4}\alpha\text{C}_{\text{o}}\text{ + 2}\beta\text{ C}_{2}\text{ + }\frac{\omega}{b}\text{ C}_{\text{Ca1}}\text{ - }\left(\beta\text{ + 3}\alpha\text{ + a}\gamma\right)C_{1}$ | E57 |
| $\frac{dC_{2}}{dt}\text{ = 3}\alpha\text{C}_{1}\text{ + 3}\beta\text{ C}_{3}\text{ + }\frac{\omega}{b^{2}}\text{ C}_{\text{Ca2}}\text{ - }\left(2\beta\text{ + 2}\alpha\text{ + a}^{2}\gamma\right)C_{2}$ | E58 |
| $\frac{dC_{3}}{dt}\text{ = 2}\alpha\text{C}_{2}\text{ + 4}\beta\text{ C}_{4}\text{ + }\frac{\omega}{b^{3}}\text{ C}_{\text{Ca3}}\text{ - }\left(3\beta\text{ + }\alpha\text{ + a}^{3}\gamma\right)C_{3}$ | E59 |
| $\frac{dC_{4}}{dt}\text{ = }\alpha\text{C}_{3}\text{ + }g\text{ O + }\frac{\omega}{b^{4}}\text{ C}_{\text{Ca4}}\text{ - }\left(4\beta\text{ + }f\text{ + a}^{4}\gamma\right)C_{4}$ | E60 |
| $\frac{dO}{dt}\text{ = }f\text{ C}_{4}\text{ - g O}$ | E61 |
| $\frac{dC_{Ca0}}{dt}\text{ = }\beta'\text{C}_{\text{Ca1}}\text{ + }\gamma\text{ }C_{0}\text{ - }\left(\text{ 4}\alpha'\text{ + }\omega\right) \text{C}_{\text{Ca0}}$ | E62 |
| $\frac{dC_{Ca1}}{dt}\text{ = 4}\alpha'\text{C}_{\text{CaO}}\text{ + 2}\beta'\text{C}_{\text{Ca2}}\text{ + a}\gamma\text{ }C_{1}\text{ - }\left(\beta'\text{ + 3}\alpha'\text{ + }\frac{\omega}{b}\right)\text{C}_{\text{Ca1}}$ | E63 |
| $\frac{dC_{Ca2}}{dt}\text{ = 3}\alpha'\text{C}_{\text{Ca1}}\text{ + 3}\beta'\text{C}_{\text{Ca3}}\text{ + a}^{2}\gamma\text{ }C2\text{ - }\left(2\beta'\text{ + 2}\alpha'\text{ + }\frac{\omega}{b^{2}}\right)\text{C}_{\text{Ca2}}$ | E64 |
| $\frac{dC_{Ca3}}{dt}\text{ = 2}\alpha'\text{C}_{\text{Ca2}}\text{ + 4}\beta'\text{C}_{\text{Ca4}}\text{ + a}^{3}\gamma\text{ }C_{3}\text{ - }\left(3\beta'\text{ + }\alpha'\text{ + }\frac{\omega}{b^{3}}\right)\text{C}_{\text{Ca3}}$ | E65 |
| $\frac{dC_{Ca4}}{dt}\text{ = }\alpha'\text{C}_{\text{Ca3}}\text{ + }g'O_{\text{Ca}}\text{ + a}^{4}\gamma\text{ }C_{4}\text{ - }\left(4\beta'\text{ + f}'\text{ + }\frac{\omega}{b^{4}}\right) \text{C}_{\text{Ca4}}$ | E66 |
|  | E67 |
| $I_{Ca_{\max}}\text{ }=\text{ 4 }\overline{\text{P}}_{\text{Ca}}\frac{\text{VF}^{2}}{\text{RT}}\text{ }\frac{\text{0.001 e}^{\text{2VF/RT}}\text{ - 0.341 [Ca}^{\text{2+}}\text{]}_{\text{o}}}{\text{e}^{\text{2V/RT}}\text{ - 1}}$ | E68 |
| $I_{Ca}\text{ }=\text{ 6 I}_{\text{Ca}_{\text{max}}}\text{ y [O]}$ | E69 |
| $I_{Ca,K}\text{ }=\text{ P}_{\text{K}}^{\text{'}}\text{ y}\left[\text{O + O}_{\text{Ca}}\right]\text{ }\frac{\text{VF}^{2}}{\text{RT}}\text{ }\frac{\text{[K}^{\text{+}}\text{]}_{\text{i}}\text{ e}^{\text{VF/RT}}\text{ - [K}^{\text{+}}\text{]}_{\text{o}}}{\text{e}^{\text{VF/RT}}\text{ - 1}}$ | E70 |
| $P_{K}^{'}\text{ = }\frac{\overline{\text{P}}_{\text{K}}}{\text{1 + }\frac{\text{I}_{\text{Ca}_{\text{max}}}}{\text{I}_{\text{Ca}_{\text{half}}}}}$ | E71 |
| $\frac{dy}{dt}\text{ = }\frac{\text{y}_{\infty}\text{ - y}}{\tau_{\text{y}}}$ | E72 |
| $y_{\infty}\text{ = }\frac{1}{\text{1 + e}^{\text{(V+55)/7.5}}}\text{ + }\frac{0.5}{\text{1+ e}^{\text{(-V+21)/6}}\text{ }}$ | E73 |
| $\tau_{y}\text{ = 20 + }\frac{600}{\text{1 + }e^{\left(V+30\right)/9.5}}$ | E74 |

### *Sarcolemmal membrane potential*

| $\frac{dV}{dt}\text{ = -}\frac{1}{C_{m}}\text{ }\left(\begin{matrix} \text{I}_{\text{Na}}\text{ + I}_{\text{Ca}}\text{ + I}_{\text{Ca,K}}\text{ + I}_{\text{K}}\text{+ I}_{\text{K}_{1}}\text{ + I}_{\text{Kp}}\text{ + I}_{\text{NaCa}} \\ \text{+ I}_{\text{NaK}}\text{ + I}_{\text{ins(Ca)}}\text{ + I}_{\text{pCa}}\text{ + I}_{\text{Ca,b}}\text{ + I}_{\text{Na,b}}+I_{K,ATP} \end{matrix}\right)$ | E80 |
| --- | --- |

**S1.2. Ca^2+^ handling system**

#### Ca^2+^ Release channel current (J_rel_)

| $\frac{dP_{C1}}{dt}\text{ = -k}_{\text{a}}^{\text{+}}\text{ [Ca}^{\text{2+}}\text{]}_{\text{ss}}^{\text{n}}\text{ P}_{\text{C1}}\text{ + k}_{\text{a}}^{\text{-}}\text{ P}_{\text{O1}}$ | E75 |
| --- | --- |
| $\begin{matrix} \frac{dP_{O1}}{dt}\text{ = k}_{\text{a}}^{\text{+}}\text{ [Ca}^{\text{2+}}\text{]}_{\text{ss}}^{\text{n}}\text{ P}_{\text{C1}}\text{ - k}_{\text{a}}^{\text{-}}\text{ P}_{\text{O1}}\text{ - k}_{\text{b}}^{\text{+}}\text{ [Ca}^{\text{2+}}\text{]}_{\text{ss}}^{\text{m}}\text{ P}_{\text{O1}}\text{ } \\ +\text{k}_{\text{b}}^{\text{-}}\text{ P}_{\text{O2}}\text{ - k}_{\text{c}}^{\text{+}}\text{ P}_{\text{O1}}\text{ + k}_{\text{c}}^{\text{-}}\text{ P}_{\text{C2}} \end{matrix}$ | E76 |
| $\frac{dP_{O2}}{dt}\text{ = k}_{\text{b}}^{\text{+}}\text{ [Ca}^{\text{2+}}\text{]}_{\text{ss}}^{\text{m}}\text{ P}_{\text{O1}}\text{ - k}_{\text{b}}^{\text{-}}\text{ P}_{\text{O2}}$ | E77 |
| $\frac{dP_{C2}}{dt}\text{ = k}_{\text{c}}^{\text{+}}\text{ P}_{\text{O1}}\text{ - k}_{\text{c}}^{\text{-}}\text{ P}_{\text{C2}}$ | E78 |
| $J_{rel}\text{ = v}_{1}\text{ }\left(\text{P}_{\text{O}_{1}}\text{ + P}_{\text{O}_{2}}\right)\text{ }\left(\text{[Ca}^{\text{2+}}\text{]}_{\text{JSR}}\text{ - [Ca}^{\text{2+}}\text{]}_{\text{SS}}\right)$ | E79 |

### *Ca^2+^ buffering and diffusive transport between compartments*

| $J_{trpn}\text{ = }\frac{d[HTRPNCa]}{dt}\text{ + }\frac{d[LTRPNCa]}{dt}$ | E81 |
| --- | --- |
| $J_{tr}\text{ = }\frac{[Ca^{2+}]_{NSR}\text{ }-\text{ [Ca}^{\text{2+}}\text{]}_{\text{JSR}}}{\tau_{tr}}\text{ }$ | E82 |
| $J_{xfer}\text{ = }\frac{[Ca^{2+}]_{SS}\text{ }-\text{ [Ca}^{\text{2+}}\text{]}_{\text{i}}}{\tau_{xfer}}$ | E83 |
| $\beta_{i}\text{ = }\left(1+\frac{K_{m}^{CMDN}[CMDN]_{tot}}{\left(K_{m}^{CMDN}\text{ + [Ca}^{\text{2+}}\text{]}_{\text{i}}\right)^{2}}\right)^{-1}$ | E84 |
| $\beta_{SS}\text{ = }\left(1+\frac{K_{m}^{CMDN}[CMDN]_{tot}}{\left(K_{m}^{CMDN}\text{ + [Ca}^{\text{2+}}\text{]}_{\text{SS}}\right)^{2}}\right)^{\text{-1}}\text{ }$ | E85 |
| $\beta_{JSR}\text{ = }\left(1+\frac{K_{m}^{CSQN}[CSQN]_{tot}}{\left(K_{m}^{CSQN}\text{ + [Ca}^{\text{2+}}\text{]}_{\text{JSR}}\right)^{2}}\right)^{-1}\text{ }$ | E86 |
| $\begin{matrix} \frac{d[HTRPNCa]}{dt} = k_{htrpn}^{+} [Ca^{2+}]_{i} \left([HTRPN]_{tot} - [HTRPNCa]\right) \\ - k_{htrpn}^{-} [HTRPNCa] \end{matrix}$ | E87 |
| $\begin{matrix} \frac{d[LTRPNCa]}{dt} = k_{ltrpn}^{+} [Ca^{2+}]_{i} \left([LTRPN]_{tot} - [LTRPNCa]\right) \\ - k_{ltrpn}^{-} \left(1 - \frac{2}{3} Force_{Norm}\right) [LTRPNCa] \end{matrix}$ | E88 |

### S1.3. Ionic concentrations balance equations

| $\frac{d[Ca^{2+}]_{i}}{dt}\text{ = }\beta_{\text{i}}\left[\begin{matrix} \text{J}_{\text{xfer}}\text{ - J}_{\text{up}}\text{ - J}_{\text{trpn}}\text{ - }\left(\text{I}_{\text{Ca,b}}\text{ - 2 I}_{\text{NaCa}}\text{ + I}_{\text{pCa}}\right)\frac{A_{cap}}{2V_{myo}F} \\ +\text{ }\left(\text{V}_{\text{NaCa}}\text{ - V}_{\text{uni}}\right)\text{ }\frac{\text{V}_{\text{mito}}}{\text{V}_{\text{myo}}} \end{matrix}\right]$ | E89 |
| --- | --- |
| $\frac{d[Ca^{2+}]_{ss}}{dt}\text{ = }\beta_{\text{ss}}\left[\text{J}_{\text{rel}}\frac{\text{V}_{\text{JSR}}}{\text{V}_{\text{ss}}}\text{ - J}_{\text{xfer}}\frac{\text{V}_{\text{myo}}}{\text{V}_{\text{ss}}}\text{ - }\left(\text{I}_{\text{Ca}}\right)\frac{A_{cap}}{2V_{ss}F}\right]$ | E90 |
| $\frac{d[Ca^{2+}]_{_{JSR}}}{dt}\text{ = }\beta_{_{\text{JSR}}}\left[\text{J}_{\text{tr}}\text{ - J}_{\text{rel}}\right]$ | E91 |
| $\frac{d[Ca^{2+}]_{NSR}}{dt}\text{ = J}_{\text{up}}\frac{\text{V}_{\text{myo}}}{\text{V}_{\text{NSR}}}\text{ - J}_{\text{tr}}\frac{\text{V}_{\text{JSR}}}{\text{V}_{\text{NSR}}}\text{ }$ | E92 |
| $\frac{d[Ca^{2+}]_{m}}{dt}\text{ = }\delta\left(\text{V}_{\text{uni}}\text{ - V}_{\text{NaCa}}\right)\text{ }$ | E93 |
| $\frac{d[Na^{+}]_{i}}{dt}\text{ = - }\left(\text{I}_{\text{Na}}\text{ + I}_{\text{Na,b}}\text{ + I}_{\text{ns,Na}}\text{ + 3 I}_{\text{NaCa}}\text{ + 3 I}_{\text{NaK}}\text{ }\right)\frac{A_{cap}}{V_{myo}F}$ | E94 |
| $\frac{d[K^{+}]_{i}}{dt}\text{ = - }\left(\text{I}_{\text{K}}\text{ + I}_{\text{K}_{1}}\text{ + I}_{\text{Kp}}\text{ + I}_{\text{ns,K}}\text{ + I}_{\text{Ca,K}}\text{ - 2 I}_{\text{NaK}}\right)\frac{A_{cap}}{V_{myo}F}$ | E95 |

### S1.4. Force generation model (1)

| $\frac{d[P_{o}]}{dt} = - \left(k_{pn}^{trop} + f_{01}\right) [P_{o}] + k_{np}^{trop} [N_{0}] + g_{01}(SL) [P_{1}]$ | E96 |
| --- | --- |
|  | E97 |
|  | E98 |
|  | E99 |
|  | E100 |
| $[N_{0}]\text{ = 1 - }\left(\text{[N}_{1}\text{] + [P}_{0}\text{] + [P}_{1}\text{] + [P}_{2}\text{] + [P}_{3}\text{]}\right)$ | E101 |
| $f_{01}\text{ = 3 }\times\text{ f}_{\text{XB}}$ | E102 |
| $f_{12}\text{ = 10 }\times\text{f}_{\text{XB}}$ | E103 |
| $f_{23}\text{ = 7 }\times\text{ f}_{\text{XB}}$ | E104 |
| $g_{01}\text{ = 1 }\times\text{ g}_{_{\text{XB}}}^{\text{min}}$ | E105 |
| $g_{12}\text{ = 2 }\times\text{ g}_{_{\text{XB}}}^{\text{min}}$ | E106 |
| $g_{23}\text{ = 3 }\times\text{ g}_{_{\text{XB}}}^{\text{min}}$ | E107 |
| $g_{01}\text{(SL) = 1 }\times\text{ }\varphi\text{ }\times\text{ g}_{_{\text{XB}}}^{\text{min}}$ | E108 |
| $g_{12}\text{(SL) = 2 }\times\text{ }\varphi\text{ }\times\text{ g}_{_{\text{XB}}}^{\text{min}}$ | E109 |
| $g_{23}\text{(SL) = 3 }\times\text{ }\varphi\text{ }\times\text{ g}_{_{\text{XB}}}^{\text{min}}$ | E110 |
|  | E111 |
| $k_{np}^{trop}\text{ = }k_{pn}^{trop}\text{ }\left[\frac{[LTRPNCa]}{\text{K}_{\text{1/2}}^{\text{trop}}\text{ }[LTRPN]_{tot}}\right]^{N^{trop}}$ | E112 |
| $K_{1/2}^{trop}\text{ = }\left(\text{1 + }\frac{\text{K}_{\text{Ca}}^{\text{trop}}}{\text{1.7 10}^{\text{-3}}\text{ - 0.8 10}^{\text{-3}}\text{ }\frac{\left(\text{SL - 1.7}\right)}{0.6}\text{ }}\right)^{-1}$ | E113 |
| $N^{trop}\text{ = 3.5 }\times\text{ SL - 2.0}$ | E114 |
| $K_{Ca}^{trop} = \frac{k_{ltrpn}^{-}}{k_{ltrpn}^{+}}$ | E115 |
| $\sum PATHS\text{ = g}_{01}\text{ g}_{12}\text{ g}_{23}\text{ + f}_{01}\text{ g}_{12}\text{ g}_{23}\text{ + f}_{01}\text{ f}_{12}\text{ g}_{23}\text{ + f}_{01}\text{ f}_{12}\text{ f}_{23}$ | E116 |
| $P1_{\max}\text{ = }\frac{\text{f}_{01}\text{ g}_{12}\text{ g}_{23}}{\sum\text{PATHS}}$ | E117 |
| $P2_{\max}\text{ = }\frac{\text{f}_{01}\text{ f}_{12}\text{ g}_{23}}{\sum\text{PATHS}}$ | E118 |
| $P3_{\max}\text{ = }\frac{\text{f}_{01}\text{ f}_{12}\text{ f}_{23}}{\sum\text{PATHS}}$ | E119 |
| $Force\text{ = }\zeta\frac{\text{P}_{1}\text{ + N}_{1}\text{ + 2 P}_{2}\text{ + 3 P}_{3}}{\text{P1}_{\text{max}}\text{ + 2 P2}_{\text{max}}\text{ + 3 P3}_{\text{max}}}$ | E120 |
| $Force_{Norm}\text{ = }\frac{\text{P}_{1}\text{ + N}_{1}\text{ + P}_{2}\text{ + P}_{3}}{\text{P1}_{\text{max}}\text{ + P2}_{\text{max}}\text{ + P3}_{\text{max}}}$ | E121 |
| $\begin{matrix} \text{V}_{\text{AM}}\text{ }=\text{ V}_{\text{AM}}^{\text{max}}\left(\frac{\text{f}_{01}\text{ [P}_{0}\text{] }+\text{ f}_{12}\text{ [P}_{1}\text{] }+\text{ f}_{23}\text{ [P}_{2}\text{]}}{\text{f}_{01}\text{ }+\text{ f}_{12}\text{ }+\text{ f}_{23}}\right) \\ \times\left(\text{1 }+\text{ }\frac{\text{K}_{\text{M,AM}}^{\text{ATP}}}{\text{[ATP]}_{\text{i}}}\text{ }\left[\text{1 }+\text{ }\frac{\text{[ADP]}_{\text{i}}}{\text{K}_{\text{i,AM}}}\right]\right)^{-1} \end{matrix}$ | E122 |

### S1.5. Mitochondrial membrane potential (ΔΨ_m_)

| $\frac{d\text{ }\Delta\Psi_{m}}{\text{dt}}\text{ }=\text{ }\frac{\text{V}_{\text{He}}\text{ }+\text{ V}_{\text{HSDH}}\text{ - V}_{\text{Hu}}\text{- V}_{\text{ANT}}\text{ - V}_{\text{HLeak }}\text{- V}_{\text{NaCa}}\text{ - 2 V}_{\text{uni}}-V_{IMAC}}{\text{C}_{\text{mito}}}$ | E123 |
| --- | --- |

### S1.6. Energy metabolism system

### *Mitochondrial metabolites balance equations*

| $\frac{d\text{ [ATP]}_{\text{i}}}{\text{dt}}=\text{ V}_{\text{ANT}}\frac{\text{V}_{\text{mito}}}{\text{V}_{\text{myo}}}\text{ -V}_{\text{CK}}^{\text{mito}}\text{-V}_{\text{AM}}\text{ - }\frac{1}{2}\text{J}_{\text{up}}\text{ - }\left(\text{I}_{\text{pCa}}\text{ + I}_{\text{NaK}}\right)\frac{A_{cap}}{V_{myo}F}$ | E124 |
| --- | --- |
| $\frac{d\text{ [ATP]}_{\text{ic}}}{\text{dt}}=\text{ -V}_{\text{CK}}^{\text{cyto}}\text{-V}_{\text{ATPase}}^{\text{cyto}}\text{ }$ | E125 |
| $\frac{d\text{ [CrP]}_{\text{i}}}{\text{dt}}=\text{ V}_{\text{CK}}^{\text{mito}}\text{- V}_{\text{tr}}^{\text{CrP}}$ | E126 |
| $\frac{d\text{ [CrP]}_{\text{ic}}}{\text{dt}}=\text{ V}_{\text{tr}}^{\text{CrP}}\text{ + V}_{\text{CK}}^{\text{cyto}}$ | E127 |
| $\frac{d\text{ [ADP]}_{\text{m}}}{\text{dt}}=\text{ V}_{\text{ANT}}\text{ - V}_{\text{ATPase}}\text{ - V}_{\text{SL}}$ | E128 |
| $[ATP]_{m}\text{ = C}_{\text{A}}\text{ - [ADP]}_{\text{m}}$ | E129 |
| $\frac{\text{d[NADH]}_{\text{m}}}{\text{dt}}\text{=-V}_{\text{O}_{2}}\text{+V}_{\text{IDH}}\text{+V}_{\text{KGDH}}\text{+V}_{\text{MDH}}\text{-V}_{\text{THD}}$ | E130 |
| $\frac{d[ISOC]}{dt}\text{ }=\text{ V}_{\text{ACO}}\text{ - V}_{\text{IDH}}$ | E131 |
| $\frac{d[\alpha KG]}{dt}\text{ }=\text{ V}_{\text{IDH}}\text{ - V}_{\text{KGDH}}\text{ }+\text{ V}_{\text{AAT}}$ | E132 |
| $\frac{d[SCoA]}{dt}\text{ }=\text{ V}_{\text{KGDH}}\text{ - V}_{\text{SL}}$ | E133 |
| $\frac{d[Suc]}{dt}\text{ }=\text{ V}_{\text{SL}}\text{ - V}_{\text{SDH}}$ | E134 |
| $\frac{d[FUM]}{dt}\text{ }=\text{ V}_{\text{SDH}}\text{ - V}_{\text{FH}}$ | E135 |
| $\frac{d[MAL]}{dt}\text{ }=\text{ V}_{\text{FH}}\text{ - V}_{\text{MDH}}$ | E136 |
| $\frac{d[OAA]}{dt}\text{ }=\text{ V}_{\text{MDH}}\text{ - V}_{\text{CS}}\text{ }-\text{ V}_{\text{AAT}}$ | E137 |
| $[CIT]\text{ = C}_{\text{Kint}}\text{ - }\left(\begin{matrix} \text{[ISOC] + [}\alpha KG]\text{ + }[SCoA]\text{ + }[Suc]\text{ } \\ \text{+ }[FUM]\text{ + }[MAL]\text{ + }[OAA] \end{matrix}\right)$ | E138 |

#### Cytosolic metabolic reaction rates

| $V_{ANT}\text{ }=\text{ V}_{\text{maxANT}}\text{ }\frac{0.75\left(\text{1 - }\frac{\text{0.25 [ATP]}_{\text{i}}\text{ }\times\text{ 0.45 [ADP]}_{\text{m}}}{\text{0.17 [ADP]}_{\text{i}}\text{ }\times\text{ 0.025 [ATP]}_{\text{m}}}\right)\left(e^{-\frac{F}{RT}\Delta\Psi_{m}}\right)}{\left(\text{1 }+\text{ }\frac{\text{0.25 [ATP]}_{\text{i}}}{\text{0.225 [ADP]}_{\text{i}}}\text{ e}\left(\frac{\text{-h}^{\text{ANT}}\text{ F }\Delta\Psi_{m}}{\text{RT}}\right)\right)\left(\text{1 }+\text{ }\frac{\text{0.45 [ADP]}_{\text{m}}}{0.025\text{ [ATP]}_{\text{m}}}\right)}$ | E139 |
| --- | --- |
| $V_{CK}^{cyto}\text{ }=\text{ }k_{CK}^{cyto}\left([ATP]_{ic}\text{ }[Cr]_{ic}\text{ }-\text{ }\frac{[ADP]_{ic}\text{ }[CrP]_{ic}}{K_{EQ}}\right)$ | E140 |
| $V_{CK}^{mito}\text{ }=\text{ }k_{CK}^{mito}\left([ATP]_{i}\text{ }[Cr]_{i}\text{ }-\text{ }\frac{[ADP]_{i}\text{ }[CrP]_{i}}{K_{EQ}}\right)$ | E141 |
| $\text{V}_{\text{tr}}^{\text{CrP}}\text{ = k}_{\text{tr}}^{\text{Cr}}\left(\text{[CrP]}_{\text{i}}\text{ - [CrP]}_{\text{ic}}\right)$ | E142 |

#### Tricarboxylic acid cycle reaction rates

| $V_{CS}\text{ }=\text{ k}_{\text{cat}}^{\text{CS}}\text{ E}_{\text{T}}^{\text{CS}}\left(\text{1 }+\text{ }\frac{\text{K}_{\text{M}}^{\text{AcCoA}}}{[AcCoA]}\text{ }+\frac{\text{K}_{\text{M}}^{\text{OAA}}}{[OAA]}\text{ }+\text{ }\frac{\text{K}_{\text{M}}^{\text{AcCoA}}}{[AcCoA]}\text{ }\frac{\text{K}_{\text{M}}^{\text{OAA}}}{[OAA]}\right)^{-1}$ | E143 |
| --- | --- |
| $V_{ACO}\text{ }=\text{ k}_{\text{f}}^{\text{ACO}}\text{ }\left(\text{[CIT] - }\frac{\text{[ISOC]}}{\text{K}_{\text{E}}^{\text{ACO}}}\right)$ | E144 |
| $f_{a}^{IDH}\text{ = }\left[\left(\text{1 +}\frac{\text{[ADP]}_{\text{m}}}{\text{K}_{\text{ADP}}^{a}}\right)\text{ }\left(\text{1 +}\frac{\text{[Ca}^{\text{2+}}\text{]}_{\text{m}}}{\text{K}_{\text{Ca}}^{a}}\right)\right]^{-1}$ | E145 |
| $f_{i}^{IDH}\text{ = }\left(1\text{ }+\text{ }\frac{\text{[NADH]}}{\text{K}_{\text{i,NADH}}}\right)$ | E146 |
| $V_{IDH}\text{ }=\text{ k}_{\text{cat}}^{\text{IDH}}\text{ E}_{\text{T}}^{\text{IDH}}\text{ }\left[\begin{matrix} \left(1\text{ }+\text{ }\frac{\text{[H}^{+}\text{]}}{\text{k}_{\text{h,1}}}\text{ }+\text{ }\frac{\text{k}_{\text{h,2}}}{\text{[H}^{+}\text{]}}\right)\text{ }+\text{ f}_{\text{i}}^{\text{IDH}}\left(\frac{\text{K}_{\text{M}}^{\text{NAD}}}{\text{[NAD]}}\right)\text{ } \\ \text{+f}_{\text{a}}^{\text{IDH}}\text{ }\left(\frac{\text{K}_{\text{M}}^{\text{ISOC}}}{\text{[ISOC]}}\right)^{ni}\text{+ f}_{\text{a}}^{\text{IDH}}\text{ f}_{\text{i}}^{\text{IDH}}\left(\frac{\text{K}_{\text{M}}^{\text{ISOC}}}{\text{[ISOC]}}\right)^{ni}\left(\frac{\text{K}_{\text{M}}^{\text{NAD}}}{\text{[NAD]}}\right) \end{matrix}\right]^{-1}$ | E147 |
| $f_{a}^{KGDH}\text{ = }\left[\left(1+\text{ }\frac{\text{[Mg}^{2+}]}{\text{K}_{\text{D}}^{\text{Mg}^{2+}}}\right)\left(1\text{ }+\text{ }\frac{\text{[Ca}^{2+}]_{m}}{\text{K}_{\text{D}}^{\text{Ca}^{2+}}}\right)\right]^{-1}$ | E148 |
| $V_{KGDH}\text{ }=\text{ }\frac{\text{ k}_{\text{cat}}^{\text{KGDH}}\text{ E}_{\text{T}}^{\text{KGDH}}}{\text{1 }+\text{ f}_{\text{a}}^{\text{KGDH}}\frac{\text{K}_{\text{M}}^{\alpha\text{KG}}}{[\alpha\text{KG]}}\text{ }+\text{ f}_{\text{a}}^{\text{KGDH}}\left(\frac{\text{K}_{\text{M}}^{\text{NAD}}}{\text{[NAD]}}\right)^{n_{aKG}}}$ | E149 |
| $V_{SL}\text{ }=\text{ k}_{\text{f}}^{\text{SL}}\text{ }\left(\text{[SCoA] [ADP]}_{\text{m}}\text{ - }\frac{\text{[Suc][ATP]}_{\text{m}}[CoA]}{\text{K}_{\text{E}}^{\text{SL}}}\right)$ | E150 |
| $V_{SDH}\text{ }=\text{ }\frac{\text{ k}_{\text{cat}}^{\text{SDH}}\text{ E}_{\text{T}}^{\text{SDH}}}{\text{1 }+\text{ }\left(\frac{\text{K}_{\text{M}}^{\text{Suc}}}{\text{[Suc]}}\right)\text{ }\left(1\text{ }+\text{ }\frac{\text{[OAA]}}{\text{K}_{\text{i,sdh}}^{\text{OAA}}}\right)\text{ }\left(1\text{ }+\text{ }\frac{\text{[FUM]}}{\text{K}_{\text{i}}^{\text{FUM}}}\right)}$ | E151 |
| $V_{FH}\text{ }=\text{ k}_{\text{f}}^{\text{FH}}\text{ }\left(\text{[FUM] - }\frac{\text{[MAL]}}{\text{K}_{\text{E}}^{\text{FH}}}\right)$ | E152 |
| $f_{h,a}\text{ }=\text{ }\left(\text{1 }+\text{ }\frac{\text{[H}^{+}\text{]}}{\text{k}_{\text{h1}}}\text{ }+\frac{\text{[H}^{+}\text{]}^{2}}{\text{k}_{\text{h1}}\text{ k}_{\text{h2}}}\right)^{-1}+\text{ k}_{\text{offset}}$ | E153 |
| $f_{h,i}\text{ }=\text{ }\left(\text{1 }+\text{ }\frac{\text{k}_{\text{h3}}}{\text{[H}^{+}\text{]}}\text{ }+\frac{\text{k}_{\text{h3}}\text{ k}_{\text{h4}}}{\text{[H}^{+}\text{]}^{2}}\right)^{-2}$ | E154 |
| $V_{MDH}\text{ }=\text{ }\frac{\text{k}_{\text{cat}}^{\text{MDH}}\text{ E}_{\text{T}}^{\text{MDH}}\text{ f}_{\text{h,a}}\text{ f}_{\text{h,i}}\text{ }}{\text{1 }+\text{ }\frac{\text{K}_{\text{M}}^{\text{MAL}}}{[MAL]}\text{ }\left(\text{1 }+\text{ }\frac{\text{[OAA]}}{\text{K}_{\text{i}}^{\text{OAA}}}\right)\text{ }+\frac{\text{K}_{\text{M}}^{\text{NAD}}}{[NAD]}\text{ }+\text{ }\frac{\text{K}_{\text{M}}^{\text{MAL}}}{[MAL]}\text{ }\left(\text{1 }+\text{ }\frac{\text{[OAA]}}{\text{K}_{\text{i}}^{\text{OAA}}}\right)\text{ }\frac{\text{K}_{\text{M}}^{\text{NAD}}}{[NAD]}}$ | E155 |
| $V_{AAT}\text{ }=\text{ k}_{\text{f}}^{\text{AAT}}\text{[OAA][GLU] }\frac{k_{ASP} \text{K}_{\text{E}}^{\text{AAT}}}{\left(k_{ASP} \text{K}_{\text{E}}^{\text{AAT}} + [\alpha KG] \text{k}_{\text{f}}^{\text{AAT}}\right)}$ | E156 |

#### Oxidative phosphorylation reaction rates

| $V_{O_{2}}\text{ }=\text{ 0.5 }\rho^{\text{res}}\text{ }\frac{\left(\text{r}_{\text{a}}\text{ }+\text{ r}_{\text{c1}}\text{ e}^{\left(\frac{\text{6F }\Delta\Psi_{\text{B}}}{\text{R T}}\right)}\right)e^{\left(\frac{A_{res}\text{ F}}{\text{R T}}\right)}\text{ - r}_{\text{a}}\text{ e}^{\left(\frac{\text{g 6 F }\Delta\mu_{\text{H}}}{\text{R T}}\right)}\text{ }+\text{ r}_{\text{c2}} e^{\left(\frac{A_{res}\text{ F}}{\text{R T}}\right)}\text{ e}^{\left(\frac{\text{g 6 F }\Delta\mu_{\text{H}}}{\text{R T}}\right)}\text{ }}{\left(\text{1 }+\text{ r}_{1}\text{ e}^{\left(\frac{\text{F A}_{\text{res}}}{\text{R T}}\right)}\right)e^{\left(\frac{6\text{ F}\Delta\Psi_{\text{B}}\text{ }}{\text{R T}}\right)}\text{ }+\text{ }\left(\text{r}_{2}\text{ }+\text{ r}_{3}\text{ e}^{\left(\frac{\text{F A}_{\text{res}}}{\text{R T}}\right)}\right)\text{ e}^{\left(\frac{\text{g 6 F }\Delta\mu_{\text{H}}}{\text{R T}}\right)}}$ | E157 |
| --- | --- |
| $V_{He}\text{ }=\text{ 6 }\rho^{\text{res}}\text{ }\frac{\left(\text{r}_{a}\text{ }e^{\left(\frac{A_{res}\text{ F}}{\text{R T}}\right)}\text{ }-\text{ }\left(\text{r}_{\text{a}}\text{ }+\text{ r}_{\text{b}}\right)\text{ e}^{\left(\frac{\text{g 6F }\Delta\mu_{\text{H}}}{\text{R T}}\right)}\right)\text{ }}{\left(\text{1 }+\text{ r}_{1}\text{ e}^{\left(\frac{\text{F A}_{\text{res}}}{\text{R T}}\right)}\right)e^{\left(\frac{6\text{ F}\Delta\Psi_{\text{B}}\text{ }}{\text{R T}}\right)}\text{ }+\text{ }\left(\text{r}_{2}\text{ }+\text{ r}_{3}\text{ e}^{\left(\frac{\text{F A}_{\text{res}}}{\text{R T}}\right)}\right)\text{ e}^{\left(\frac{\text{g 6 F }\Delta\mu_{\text{H}}}{\text{R T}}\right)}}$ | E158 |
| $A_{res}\text{ }=\text{ }\frac{\text{R T}}{\text{F}}\text{ ln }\left(\text{K}_{\text{res}}\text{ }\sqrt{\frac{\text{[NADH]}}{\text{[NAD}^{+}]}}\right)$ | E159 |
| [NAD^+^] = C_PN_ - [ NADH] | E160 |
| $V_{HSDH}\text{ }=\text{ 4 }\rho^{\text{res(F)}}\text{ }\frac{\left(\text{r}_{a}\text{ }e^{\left(\frac{A_{res(F)}\text{ F}}{\text{R T}}\right)}\text{ }-\text{ }\left(\text{r}_{\text{a}}\text{ }+\text{ r}_{\text{b}}\right)\text{ e}^{\left(\frac{\text{g 4F }\Delta\mu_{\text{H}}}{\text{R T}}\right)}\right)\frac{1}{1+\frac{[OAA]}{K_{i}^{OAA}}}\text{ }}{\left(\text{1 }+\text{ r}_{1}\text{ e}^{\left(\frac{\text{F A}_{\text{res(F)}}}{\text{R T}}\right)}\right)e^{\left(\frac{4\text{ F}\Delta\Psi_{\text{B}}\text{ }}{\text{R T}}\right)}\text{ }+\text{ }\left(\text{r}_{2}\text{ }+\text{ r}_{3}\text{ e}^{\left(\frac{\text{F A}_{\text{res(F)}}}{\text{R T}}\right)}\right)\text{ e}^{\left(\frac{\text{g 4 F }\Delta\mu_{\text{H}}}{\text{R T}}\right)}}$ | E161 |
|  | E162 |
| $V_{ATPase}\text{ }=\text{ - }\rho^{\text{F1}}\text{ }\frac{\left(\text{ 10}^{\text{2 }}\text{p}_{\text{a}}\text{ }+\text{ p}_{\text{c1}}\text{ e}^{\left(\frac{\text{3 F }\Delta\Psi_{\text{B}}}{\text{R T}}\right)}\right)e^{\left(\frac{A_{F1}\text{ F}}{\text{R T}}\right)}\text{ - }\left(\text{p}_{\text{a}}\text{ e}^{\left(\frac{\text{3 F }\Delta\mu_{\text{H}}}{\text{R T}}\right)}\text{ }+\text{ p}_{\text{c2}}\text{ }e^{\left(\frac{A_{F1}\text{ F}}{\text{R T}}\right)}\text{ e}^{\left(\frac{\text{3 F }\Delta\mu_{\text{H}}}{\text{R T}}\right)}\right)\text{ }}{\left(\text{1 }+\text{ p}_{1}\text{ e}^{\left(\frac{\text{F A}_{\text{F1}}}{\text{R T}}\right)}\right)e^{\left(\frac{3\text{ F}\Delta\Psi_{\text{B}}\text{ }}{\text{R T}}\right)}\text{ }+\text{ }\left(\text{p}_{2}\text{ }+\text{ p}_{3}\text{ e}^{\left(\frac{\text{F A}_{\text{F1}}}{\text{R T}}\right)}\right)\text{ e}^{\left(\frac{\text{3 F }\Delta\mu_{\text{H}}}{\text{R T}}\right)}}$ | E163 |
| $V_{Hu}\text{ }=\text{ -3 }\rho^{\text{F1}}\text{ }\frac{\text{ 10}^{2}p_{a}\text{ }\left(\text{1 }+\text{ e}^{\left(\frac{\text{F A}_{\text{F1}}}{\text{R T}}\right)}\right)\text{ - }\left(\text{p}_{\text{a}}\text{ }+\text{ p}_{\text{b}}\right)\text{ e}^{\left(\frac{\text{3 F }\Delta\mu_{\text{H}}}{\text{R T}}\right)}\text{ }}{\left(\text{1 }+\text{ p}_{1}\text{ e}^{\left(\frac{\text{F A}_{\text{F1}}}{\text{R T}}\right)}\right)e^{\left(\frac{3\text{ F}\Delta\Psi_{\text{B}}\text{ }}{\text{R T}}\right)}\text{ }+\text{ }\left(\text{p}_{2}\text{ }+\text{ p}_{3}\text{ e}^{\left(\frac{\text{F A}_{\text{F1}}}{\text{R T}}\right)}\right)\text{ e}^{\left(\frac{\text{3 F }\Delta\mu_{\text{H}}}{\text{R T}}\right)}}$ | E164 |
| $A_{F1}\text{ }=\text{ }\frac{\text{R T}}{\text{F}}\text{ ln }\left(\text{K}_{\text{F1}}\text{ }\frac{\text{[ATP]}_{\text{m}}}{[ADP]_{m}\text{ Pi}}\right)$ | E165 |
| $V_{Hleak}\text{ }=\text{ g}_{\text{H}}\text{ }\Delta\mu_{\text{H}}$ | E166 |
| $\Delta\mu_{H}\text{ }=\text{ -2.303}\frac{\text{R T}}{\text{F}}\text{ }\Delta\text{pH }+\text{ }\Delta\Psi_{m}$ | E167 |

### *Mitochondrial Ca^2+^ handling rates*

| $V_{uni1}\text{ }=\text{ V}_{\text{max,1}}^{\text{uni}}\frac{\frac{[Ca^{2+}]_{i}}{K_{trans}}\left(\text{1 }+\text{ }\frac{[Ca^{2+}]_{i}}{K_{trans}}\right)^{3}\text{ }\frac{\text{2 F }\left(\Delta\Psi_{m}\text{ - }\Delta\Psi^{o}\right)}{R\text{ T}}}{\left(\left(\text{1 }+\text{ }\frac{[Ca^{2+}]_{i}}{K_{trans}}\right)^{4}\text{ }+\text{ }\frac{L}{\left(\text{1 }+\text{ }\frac{[Ca^{2+}]_{i}}{K_{act}}\right)^{n_{a}}}\right)\text{ }\left(\text{1 - e }^{\left\{\frac{\text{-2 F }\left(\Delta\Psi_{m}\text{ - }\Delta\Psi^{\text{o}}\right)}{R\text{ T}}\right\}}\right)}$ | E168.1 |
| --- | --- |
| $V_{uni2}\text{ }=\text{ V}_{\text{max,2}}^{\text{uni}}\frac{\frac{[Ca^{2+}]_{SS}}{K_{trans}}\left(\text{1 }+\text{ }\frac{[Ca^{2+}]_{SS}}{K_{trans}}\right)^{3}\text{ }\frac{\text{2 F }\left(\Delta\Psi_{m}\text{ - }\Delta\Psi^{o}\right)}{R\text{ T}}}{\left(\left(\text{1 }+\text{ }\frac{[Ca^{2+}]_{SS}}{K_{trans}}\right)^{4}\text{ }+\text{ }\frac{L}{\left(\text{1 }+\text{ }\frac{[Ca^{2+}]_{SS}}{K_{act}}\right)^{n_{a}}}\right)\text{ }\left(\text{1 - e }^{\left\{\frac{\text{-2 F }\left(\Delta\Psi_{m}\text{ - }\Delta\Psi^{\text{o}}\right)}{R\text{ T}}\right\}}\right)}$ | E168.2 |
|  | E169 |
| $\frac{d[Ca^{2+}]_{m}}{dt}=\delta(V_{uni}-V_{NaCa})$ | E170 |

### *Rates of antioxidant system*

| $V_{GPXm}=\frac{E_{T}^{GPXm}[H_{2}O_{2}]_{m}[GSH]_{m}}{\Phi_{1}[GSH]_{m}+\Phi_{2}[H_{2}O_{2}]_{m}}$ | E171 |
| --- | --- |
| $V_{GPXi}=\frac{E_{T}^{GPXi}[H_{2}O_{2}]_{i}[GSH]_{i}}{\Phi_{1}[GSH]_{i}+\Phi_{2}[H_{2}O_{2}]_{i}}$ | E172 |
| $V_{GRm}=\frac{k_{GR}^{1}E_{T}^{GRm}}{1+\frac{K_{M}^{GSSG}}{[GSSG]m}+\frac{K_{M}^{NADPH}}{[NADPH]_{m}}+\frac{K_{M}^{GSSG}}{[GSSG]_{m}}\frac{K_{M}^{NADPH}}{[NADPH]_{m}}}$ | E173 |
| $V_{GRi}=\frac{k_{GR}^{1}E_{T}^{GRi}}{1+\frac{K_{M}^{GSSG}}{[GSSG]_{i}}+\frac{K_{M}^{NADPH}}{[NADPH]_{i}}+\frac{K_{M}^{GSSG}}{[GSSG]_{i}}\frac{K_{M}^{NADPH}}{[NADPH]_{i}}}$ | E174 |
| $V_{GRXm}=\frac{k_{grx{}_{m}}K_{eq}^{GRX}\left(\left[GSH\right]_{m}\right)^{2}GrxT\left[PSSG\right]_{m}}{\left(\left[GSSG\right]_{m}+K_{eq}^{GRX}\left(\left[GSH\right]_{m}\right)^{2}\right)\left(\frac{K_{eq}^{GRX}\left(\left[GSH\right]_{m}\right)^{2}GrxT}{\left[GSSG\right]_{m}+K_{eq}^{GRX}\left(\left[GSH\right]_{m}\right)^{2}}+K_{m}^{Grx}\right)\left(\left[PSSG\right]_{m}+K_{m}^{PSSG}\right)}$ | E175 |
| $V_{GRXi}=\frac{k_{grx{}_{i}}K_{eq}^{GRX}\left(\left[GSH\right]_{i}\right)^{2}GrxT\left[PSSG\right]_{i}}{\left(\left[GSSG\right]_{i}+K_{eq}^{GRX}\left(\left[GSH\right]_{i}\right)^{2}\right)\left(\frac{K_{eq}^{GRX}\left(\left[GSH\right]_{i}\right)^{2}GrxT}{\left[GSSG\right]_{i}+K_{eq}^{GRX}\left(\left[GSH\right]_{i}\right)^{2}}+K_{m}^{Grx}\right)\left(\left[PSSG\right]_{i}+K_{m}^{PSSG}\right)}$ | E176 |
| $V_{PSSG_{m}}=\frac{k_{PSH}^{1}E_{T}^{PSH}\left(PSSGT-\left[PSSG\right]_{m}\right)}{\left(1+\frac{K_{M}^{GSH}}{\left[GSH\right]_{m}}\right)\left(1+\frac{[H_{2}O_{2}]_{m}}{K_{act}^{H_{2}O_{2}}}\right)}$ | E177 |
| $V_{PSSG_{i}}=\frac{k_{PSH}^{1}E_{T}^{PSH}\left(PSSGT-\left[PSSG\right]_{i}\right)}{\left(1+\frac{K_{M}^{GSH}}{\left[GSH\right]_{i}}\right)\left(1+\frac{[H_{2}O_{2}]_{i}}{K_{act}^{H_{2}O_{2}}}\right)}$ | E178 |
| $V_{MnSOD}=\frac{2k_{SOD}^{1}k_{SOD}^{5}\left(k_{SOD}^{1}+k_{SOD}^{3}\left(1+\frac{[H_{2}O_{2}]_{m}}{K_{i}^{H_{2}O_{2}}}\right)\right)E_{MnSOD}^{T}[O_{2}^{\bullet-}]_{m}}{k_{SOD}^{5}\left(2k_{SOD}^{1}+k_{SOD}^{3}\left(1+\frac{[H_{2}O_{2}]_{m}}{K_{i}^{H_{2}O_{2}}}\right)\right)+[O_{2}^{\bullet-}]_{m}k_{SOD}^{1}k_{SOD}^{3}\left(1+\frac{[H_{2}O_{2}]_{m}}{K_{i}^{H_{2}O_{2}}}\right)}$ | E179 |
| $V_{CuZnSOD}=\frac{2k_{SOD}^{1}k_{SOD}^{5}\left(k_{SOD}^{1}+k_{SOD}^{3}\left(1+\frac{[H_{2}O_{2}]_{i}}{K_{i}^{H_{2}O_{2}}}\right)\right)E_{CuZnSOD}^{T}[O_{2}^{\bullet-}]_{i}}{k_{SOD}^{5}\left(2k_{SOD}^{1}+k_{SOD}^{3}\left(1+\frac{[H_{2}O_{2}]_{i}}{K_{i}^{H_{2}O_{2}}}\right)\right)+[O_{2}^{\bullet-}]_{i}k_{SOD}^{1}k_{SOD}^{3}\left(1+\frac{[H_{2}O_{2}]_{i}}{K_{i}^{H_{2}O_{2}}}\right)}$ | E180 |
|  | E181 |
| $V_{H2O2diff}=c_{H2O2diff}([H_{2}O_{2}]_{m}-[H_{2}O_{2}]_{i})$ | E182 |
|  | E183 |
| $G_{T}=G_{T}-\left[GSH\right]_{m}-\left[GSH\right]_{i}-2\left[GSSG\right]_{m}-2\left[GSSG\right]_{i}-\left[PSSG\right]_{i}-\left[PSSG\right]_{m}$ | E184 |
| $\left[GSSG\right]_{i}=0.5\left(G_{T}-\left[GSH\right]_{m}-\left[GSH\right]_{i}-2\left[GSSG\right]_{m}-\left[PSSG\right]_{i}-\left[PSSG\right]_{m}\right)$ | E185 |
| $V_{TxPX_{m}}=\frac{E_{T}^{\Pr x3m}[H_{2}O_{2}]_{m}[TrxSH_{2}]_{m}}{\Phi_{1\Pr x}[TrxSH_{2}]_{m}+\Phi_{2\Pr x}[H_{2}O_{2}]_{m}}$ | E186 |
| $V_{TxPX_{i}}=\frac{E_{T}^{\Pr x3i}[H_{2}O_{2}]_{i}[TrxSH_{2}]_{i}}{\Phi_{1\Pr x}[TrxSH_{2}]_{i}+\Phi_{2\Pr x}[H_{2}O_{2}]_{i}}$ | E187 |
| $V_{TxR_{m}}=\frac{k_{TrxR}^{1}E_{T}^{TrxR2m}}{1+\frac{K_{M}^{TrxSS}}{[TrxSS]_{m}}+\frac{K_{Mtrx}^{NADPH}}{[NADPH]_{m}}+\frac{K_{M}^{TrxSS}}{[TrxSS]_{m}}\frac{K_{Mtrx}^{NADPH}}{[NADPH]_{m}}}$ | E188 |
| $V_{TxR_{i}}=\frac{k_{TrxR}^{1}E_{T}^{TrxR2i}}{1+\frac{K_{M}^{TrxSS}}{[TrxSS]_{i}}+\frac{K_{Mtrx}^{NADPH}}{[NADPH]_{i}}+\frac{K_{M}^{TrxSS}}{[TrxSS]_{i}}\frac{K_{Mtrx}^{NADPH}}{[NADPH]_{i}}}$ | E189 |
| $[TrxSS]_{m}=TrxT_{m}-[TrxSH_{2}]_{m}$ | E190 |
| $[TrxSS]_{i}=TrxT_{i}-[TrxSH_{2}]_{i}$ | E191 |
| $V_{CAT}=2k_{CAT}^{1}E_{CAT}^{T}[H_{2}O_{2}]_{i}e^{-fr[H_{2}O_{2}]_{i}}$ | E192 |
| $NADP_{m}=C_{NADP_{m}}-[NADPH]_{m}$ | E193 |
| $\begin{matrix} V_{THDen}=1+\frac{[NADH]_{m}}{k_{m\_THD}^{NADHm}}+\frac{NAD}{k_{m\_THD}^{NAD}}+\frac{NADP_{m}}{k_{m\_THD}^{NADP}}+\frac{[NADPH]_{m}}{k_{m\_THD}^{NADPH}}+\frac{[NADH]_{m}}{k_{m\_THD}^{NADHm}}\frac{NADP_{m}}{k_{m\_THD}^{NADP}}e^{(F/10RT)\cdot\Delta\mu_{H}}+ \\ \frac{[NADPH]_{m}}{k_{m\_THD}^{NADPH}}\frac{[NADH]_{m}}{k_{m\_THD}^{NADHm}}e^{(1-(F/10RT)\cdot\Delta\mu_{H})}+\frac{NAD}{k_{m\_THD}^{NAD}}\frac{NADP_{m}}{k_{m\_THD}^{NADP}}e^{(F/10RT)\cdot\Delta\mu_{H}}e^{(1-(F/10RT)\cdot\Delta\mu_{H})}+ \\ \frac{[NADH]_{m}}{k_{m\_THD}^{NADHm}}\frac{[NADPH]_{m}}{k_{m\_THD}^{NADPH}} \end{matrix}$ | E194 |
| $V_{THD}=\frac{E_{T}^{THD}k_{a}^{THD}\frac{[NADH]_{m}}{k_{m\_THD}^{NADHm}}\frac{NADP_{m}}{k_{m\_THD}^{NADP}}e^{(F/10RT)\cdot\Delta\mu_{H}}-E_{T}^{THD}k_{b}^{THD}\frac{NAD}{k_{m\_THD}^{NAD}}\frac{[NADPH]_{m}}{k_{m\_THD}^{NADPH}}e^{(1-(F/10RT)\cdot\Delta\mu_{H})}}{V_{THDen}}$ | E195 |

### *Antioxidant system balance equations*

| $\frac{\text{d[O}_{2}^{\text{•-}}\text{]}_{\text{m}}}{\text{dt}}\text{=shunt*V}_{\text{O}_{2}}\text{-V}_{\text{MnSOD}}\text{-V}_{\text{ROS}}^{\text{Tr}}$ | E196 |
| --- | --- |
| $\frac{\text{d[O}_{2}^{\text{•-}}\text{]}_{\text{i}}}{\text{dt}}\text{=}\frac{\text{V}_{\text{mito}}}{\text{V}_{\text{i}}}\text{V}_{\text{ROS}}^{\text{Tr}}\text{-V}_{\text{CuZnSOD}}$ | E197 |
| $\frac{\text{d[H}_{2}\text{O}_{2}\text{]}_{\text{m}}}{\text{dt}}\text{=V}_{\text{MnSOD}}\text{-V}_{\text{GPXm}}\text{-V}_{\text{TxPXm}}\text{-V}_{\text{H2O2diff}}$ | E198 |
| $\frac{\text{d[H}_{2}\text{O}_{2}\text{]}_{\text{i}}}{\text{dt}}\text{=V}_{\text{CuZnSOD}}\text{-V}_{\text{GPXi}}\text{-V}_{\text{TxPXi}}\text{-V}_{\text{CAT}}+\frac{\text{V}_{\text{mito}}}{\text{V}_{\text{i}}}\text{V}_{\text{H2O2diff}}$ | E199 |
| $\frac{\text{d[GSH]}_{\text{m}}}{\text{dt}}\text{=V}_{\text{GRm}}\text{-V}_{\text{GPXm}}\text{-V}_{\text{GRXm}}\text{-V}_{\text{PSSGm}}$ | E200 |
| $\frac{\text{d[GSH]}_{\text{i}}}{\text{dt}}\text{=V}_{\text{GRi}}\text{-V}_{\text{GPXi}}\text{-V}_{\text{GRXi}}\text{-V}_{\text{PSSGi}}$ | E201 |
| $\frac{\text{d[GSSG]}_{\text{m}}}{\text{dt}}\text{=0.5}\left(\text{V}_{\text{GPXm}}\text{-V}_{\text{GRm}}\right)\text{+V}_{\text{GRXm}}$ | E202 |
| $\frac{\text{d[TxR]}_{\text{m}}}{\text{dt}}\text{=V}_{\text{TxRm}}\text{-V}_{\text{TxPXm}}$ | E203 |
| $\frac{\text{d[TxR]}_{\text{i}}}{\text{dt}}\text{=V}_{\text{TxRi}}\text{-V}_{\text{TxPXi}}$ | E204 |
| $\frac{\text{d[PSSG]}_{\text{m}}}{\text{dt}}\text{=V}_{\text{PSSGm}}\text{-V}_{\text{GRXm}}$ | E205 |
| $\frac{\text{d[PSSG]}_{\text{i}}}{\text{dt}}\text{=V}_{\text{PSSGi}}\text{-V}_{\text{GRXi}}$ | E206 |
| $\frac{\text{d[NADPH]}_{\text{m}}}{\text{dt}}\text{=V}_{\text{THD}}\text{-V}_{\text{GRm}}\text{-V}_{\text{TxRm}}$ | E207 |

### *Mitochondrial ionic concentrations balance equations*

| $\frac{\text{d[H}^{\text{+}}\text{]}_{\text{m}}}{\text{dt}}\text{=δ}_{\text{Hm}}\left(\text{-V}_{\text{He}}\text{-V}_{\text{HSDH}}\text{+V}_{\text{Hu}}\text{+V}_{\text{NaH}}\text{+V}_{\text{PiC}}\text{+V}_{\text{KH}}\text{+V}_{\text{leak}}\right)$ | E208 |
| --- | --- |
| $\frac{\text{d[Pi]}_{\text{m}}}{\text{dt}}\text{=δ}_{\text{Pim}}\left(\text{-V}_{\text{ATPase}}\text{+V}_{\text{PiC}}\text{-V}_{\text{SL}}\right)$ | E209 |
| $\frac{\text{d[Na}^{\text{+}}\text{]}_{\text{m}}}{\text{dt}}\text{=-V}_{\text{NaH}}\text{+3V}_{\text{NaCa}}$ | E210 |

### *Mitochondrial ion transportations*

| $\text{V}_{\text{NaH}}\text{=c}_{\text{NHE}}\frac{\frac{\text{β}_{1}^{\text{+}}\text{β}_{2}^{\text{+}}\text{-β}_{1}^{\text{-}}\text{β}_{2}^{\text{-}}}{\text{β}_{1}^{\text{+}}\text{+β}_{2}^{\text{+}}\text{-β}_{1}^{\text{-}}\text{+β}_{2}^{\text{-}}}}{\text{1+10}^{\text{n}_{\text{i}}\text{(pH}_{\text{i}}\text{-pK}_{\text{i}}\text{)}}}$ | E211 |
| --- | --- |
| $\text{β}_{1}^{\text{+}}\text{=}\frac{\text{k}_{1}^{\text{+}}\text{K}_{\text{H\_NHE}}\text{[Na}^{\text{+}}\text{]}_{\text{m}}}{\text{K}_{\text{H\_NHE}}\text{[Na}^{\text{+}}\text{]}_{\text{m}}\text{+K}_{\text{H\_NHE}}\text{K}_{\text{Na\_NHE}}\text{+K}_{\text{Na\_NHE}}\text{[H}^{\text{+}}\text{]}_{\text{m}}}$ | E212 |
| $\text{β}_{2}^{\text{+}}\text{=}\frac{\text{k}_{4}^{\text{+}}\text{K}_{\text{Na\_NHE}}\text{[H}^{\text{+}}\text{]}_{\text{i}}}{\text{K}_{\text{H\_NHE}}\text{[Na}^{\text{+}}\text{]}_{\text{i}}\text{+K}_{\text{H\_NHE}}\text{K}_{\text{Na\_NHE}}\text{+K}_{\text{Na\_NHE}}\text{[H}^{\text{+}}\text{]}_{\text{i}}}$ | E213 |
| $\text{β}_{1}^{\text{-}}\text{=}\frac{\text{k}_{1}^{\text{-}}\text{K}_{\text{H\_NHE}}\text{[Na}^{\text{+}}\text{]}_{\text{i}}}{\text{K}_{\text{H\_NHE}}\text{[Na}^{\text{+}}\text{]}_{\text{i}}\text{+K}_{\text{H\_NHE}}\text{K}_{\text{Na\_NHE}}\text{+K}_{\text{Na\_NHE}}\text{[H}^{\text{+}}\text{]}_{\text{i}}}$ | E214 |
| $\text{β}_{2}^{\text{-}}\text{=}\frac{\text{k}_{4}^{\text{-}}\text{K}_{\text{Na\_NHE}}\text{[H}^{\text{+}}\text{]}_{\text{m}}}{\text{K}_{\text{H\_NHE}}\text{[Na}^{\text{+}}\text{]}_{\text{m}}\text{+K}_{\text{H\_NHE}}\text{K}_{\text{Na\_NHE}}\text{+K}_{\text{Na\_NHE}}\text{[H}^{\text{+}}\text{]}_{\text{m}}}$ | E215 |
| $\begin{matrix} \text{V}_{\text{KHen}}\text{=1+}\frac{\text{[H}^{\text{+}}\text{]}_{\text{i}}}{\text{K}_{\text{H\_KHE}}}\text{+}\frac{\text{[K}^{\text{+}}\text{]}_{\text{m}}}{\text{K}_{\text{K\_KHE}}}\text{+}\frac{\text{[H}^{\text{+}}\text{]}_{\text{i}}}{\text{K}_{\text{H\_KHE}}}\frac{\text{[K}^{\text{+}}\text{]}_{\text{m}}}{\text{K}_{\text{K\_KHE}}}\text{+}\frac{\text{[H}^{\text{+}}\text{]}_{\text{m}}}{\text{K}_{\text{H\_KHE}}}\text{+} \\ \frac{\text{[K}^{\text{+}}\text{]}_{\text{i}}}{\text{K}_{\text{K\_KHE}}}\text{+}\frac{\text{[H}^{\text{+}}\text{]}_{\text{m}}}{\text{K}_{\text{H\_KHE}}}\frac{\text{[K}^{\text{+}}\text{]}_{\text{i}}}{\text{K}_{\text{K\_KHE}}} \end{matrix}$ | E216 |
| $\text{V}_{\text{KH}}\text{=}\frac{\frac{\text{c}_{\text{KHE}}}{\text{K}_{\text{H\_KHE}}\text{K}_{\text{K\_KHE}}}\left(\text{[K}^{\text{+}}\text{]}_{\text{i}}\text{[H}^{\text{+}}\text{]}_{\text{m}}\text{-δ}_{\text{KH}}\text{[K}^{\text{+}}\text{]}_{\text{m}}\text{[H}^{\text{+}}\text{]}_{\text{i}}\right)}{\text{V}_{\text{KHen}}}$ | E217 |
| $\begin{matrix} J_{PiCen}=1+\frac{[H_{2}PO_{4}^{2-}]_{i}}{K_{Pi,i}}+\frac{[OH^{-}]_{m}}{K_{OH,m}}+\frac{[H_{2}PO_{4}^{2-}]_{m}}{K_{Pi,m}}+\frac{[OH^{-}]_{i}}{K_{OH,i}}+ \\ \frac{[H_{2}PO_{4}^{2-}]_{m}[OH^{-}]_{i}}{K_{Pi,m}K_{OH,i}}\frac{[H_{2}PO_{4}^{2-}]_{i}[OH^{-}]_{m}}{K_{Pi,i}K_{OH,m}} \end{matrix}$ | E218 |
| $\text{J}_{\text{PiC}}\text{=c}_{\text{PiC}}\frac{\text{V}_{\text{PIC,f}}\frac{\text{[H}_{2}\text{PO}_{4}^{\text{2-}}\text{]}_{\text{i}}\text{[OH}^{\text{-}}\text{]}_{\text{m}}}{\text{K}_{\text{Pi,i}}\text{K}_{\text{OH,m}}}\text{-V}_{\text{PIC,b}}\frac{\text{[H}_{2}\text{PO}_{4}^{\text{2-}}\text{]}_{\text{m}}\text{[OH}^{\text{-}}\text{]}_{\text{i}}}{\text{K}_{\text{Pi,m}}\text{K}_{\text{OH,i}}}}{\text{J}_{\text{PiCen}}}$ | E219 |
