# Supplementary material for: Inhibiting Na+/K+ ATPase Can Impair Mitochondrial Energetics and Induce Abnormal Ca2+ Cycling and Automaticity in Guinea Pig Cardiomyocytes
Source: PLoS One. 2014 Apr 10;9(4):e93928. doi: 10.1371/journal.pone.0093928 (PMC3983106; doi:10.1371/journal.pone.0093928)
Supplement: File S2 — Supplemental Information model parameters. (DOCX) [file pone.0093928.s004.docx]

## S2. Supplemental Information model parameters

### S2.1. General parameters

| Symbol | Value | Units | Description | Eq. | Ref. |
| --- | --- | --- | --- | --- | --- |
| *F* | 96.5 | C mmol^-1^ | Faraday constant |  |  |
| *T* | 310 | K | Absolute temperature |  |  |
| *R* | 8.314 | J mol^-1^ K^-1^ | Universal gas constant |  |  |
| C_m_ | 1.0 | µF cm^-2^ | Membrane capacitance | E79 | 2 |
| A_cap_ | 1.534 10^-4^ | cm^2^ | Capacitative cell surface area | E88 | 2 |
| *V*_myo_ | 25.84 | pL | Cytosolic volume | E88 | 2 |
| *V*_mito_ | 15.89 | pL | Mitochondrial volume | E92 | 11 |
| *V*_NSR_ | 1.4 | pL | NSR volume | E91 | 3 |
| *V*_JSR_ | 0.16 | pL | JSR volume | E89 | 3 |
| *V*_SS_ | 0.495 10^-3^ | pL | SS volume | E89 | 3 |
| [K^+^]_o_ | 5.4 | mM | Extracellular K^+^ concentration | E17 | 2 |
| [Na^+^]_o_ | 140.0 | mM | Extracellular Na^+^ concentration | E2 | 2 |
| [Ca^2+^]_o_ | 2.0 | mM | Extracellular Ca^2+^ concentration | E32 | 2 |

### S2.2. Sarcolemmal membrane current parameters

| Symbol | Value | Units | Description | Eq. | Ref. |
| --- | --- | --- | --- | --- | --- |
| $\overline{\text{G}}_{\text{Na}}$ | 12.8 | mS µF^-1^ | Maximal Na channel conductance | E1 | 2 |
| $\overline{\text{G}}_{\text{K}_{\text{P}}}$ | 8.28 × 10^-3^ | mS µF^-1^ | Maximal plateau K channel conductance | E29 | 2 |
| P_Na,K_ | 0.01833 |  | Na+ permeability of K+ channel | E17 | 2 |
| k_NaCa_ | 9000 | µA µF^-1^ | Scaling factor of Na­^+^/Ca^+^ exchange | E32 | 3 |
| k_m,Na_ | 87.5 | mM | Na half saturation constant NCX | E32 | 2 |
| k_m,Ca_ | 1.38 | mM | Na half saturation constant NCX | E32 | 2 |
| k_sat_ | 0.1 |  | Na^+^/Ca^2+^ exchange saturation factor at negative potentials | E32 | 2 |
| *η* | 0.35 |  | Controls voltage dependence of NCX | E32 | 2 |

### S2.3. Na^+^/K^+^ pump parameters

| Symbol | Value | Units | Description | Eq. | Ref. |
| --- | --- | --- | --- | --- | --- |
| __ | 3.147 | µA µF^-1^ | Maximum Na^+^/K^+^ pump current | E33 | 12,13 |
| K_m,Nai_ | 10 | mM | Na half saturation for Na^+^/K^+^ pump | E33 | 2 |
| K_m,Ko_ | 1.5 | mM | K half saturation for Na^+^/K^+^ pump | E33 | 2 |
| $K_{NaK}^{1,ATP}$ | 8.0 × 10^-3^ | mM | ATP half saturation constant for Na^+^/K^+^ pump | E35 | 5 |
| $K_{NaK}^{i,ADP}$ | 0.1 | mM | ADP inhibition constant for Na^+^/K^+^ pump | E35 | 5 |

### S2.4. Non-specific channel current parameters

| Symbol | Value | Units | Description | Eq. | Ref. |
| --- | --- | --- | --- | --- | --- |
| $P_{ns(Na)}$ | 1.75 × 10^-7^ | cm s^-1^ | Non specific channel current Na permeability | E38 | 7 |
| K_m,ns(Ca)_ | 1.2 × 10^-3^ | mM | Ca^2+^ half saturation constant for non specific current | E37 | 2 |
| $P_{ns(K)}$ | 0 | cm s^-1^ | Non specific channel current K permeability | E40 | 3 |

e. Luo and Rudy (20)

### S2.5. Background Ca^2+^ current parameters

| Symbol | Value | Units | Description | Eq. | Ref. |
| --- | --- | --- | --- | --- | --- |
| $\bar{G}_{Ca,b}$ | 3.217 × 10^-3^ | mS µF^-1^ | Maximum background current Ca^2+^ conductance | E41 | * |
| $\bar{G}_{Na,b}$ | 5.45 × 10^-4^ | mS µF^-1^ | Maximum background current Na+ conductance | E43 | * |

Note: *. Adjusted from (2, 3) based on physiological levels of Ca^2+^ and Na^+^ in cardiomyocytes (10, 12).

### S2.6. Sarcolemmal Ca^2+^ current parameters

| Symbol | Value | Units | Description | Eq. | Ref. |
| --- | --- | --- | --- | --- | --- |
| I_pCa_max_ | 0.575 | µA µF^-1^ | Maximum sarcolemmal Ca^2+^ pump current | E45 | 3 |
| $K_{m}^{pCa}$ | 5 × 10^-4^ | mM | Ca^2+^ half saturation constant for sarcolemmal Ca^2+^ pump | E45 | 2 |
| *$K_{m1\_pCa}^{ATP}$* | 0.012 | mM | First ATP half saturation constant for sarcolemmal Ca^2+^ pump | E46 | 14 |
| *$K_{m2\_pCa}^{ATP}$* | 0.23 | mM | Second ATP half saturation constant for sarcolemmal Ca^2+^ pump | E46 | 14 |
| *$K_{i\_pCa}^{ADP}$* | 1.0 | mM | ADP inhibition constant for sarcolemmal Ca^2+^ pump | E46 | 8 |

### S2.7. Sarcoplasmic reticulum Ca^2+^ ATPase parameters

| Symbol | Value | Units | Description | Eq. | Ref. |
| --- | --- | --- | --- | --- | --- |
| V_max,f_ | 2.989 × 10^-4^ | ms^-1^ | SERCA forward rate parameter | E47 | 2 |
| V_max,r_ | 3.179 × 10^-4^ | ms^-1^ | SERCA reverse rate parameter | E47 | 2 |
| K_fb_ | 2.4 × 10^-4^ | mM | Forward Ca^2+^ half saturation constant of SERCA | E48 | 3 |
| K_rb_ | 1.64269 | mM | Reverse Ca^2+^ half saturation constant of SERCA | E49 | 3 |
| N_fb_ | 1.4 |  | Forward cooperativity constant of SERCA | E48 | 3 |
| N_rb_ | 1.0 |  | Reverse cooperativity constant of SERCA | E49 | 3 |
| *$K_{m,up}^{ATP}$* | 0.01 | mM | ATP half saturation constant for SERCA | E50 | 5 |
| *$K_{i,up}$* | 0.14 | mM | ADP first inhibition constant for SERCA | E50 | 5 |
| *$K_{i,up}^{'}$* | 5.1 | mM | ADP second inhibition constant for SERCA | E50 | 5 |

### S2.8. L-type Ca^2+^ current parameters

| Symbol | Value | Units | Description | Eq. | Ref. |
| --- | --- | --- | --- | --- | --- |
| a | 2.0 |  | Mode transition parameter | E53 | 2 |
| b | 2.0 |  | Mode transition parameter | E54 | 2 |
| *ω* | 0.01 | ms^-1^ | Mode transition parameter | E56 | 2 |
| f | 0.3 | ms^-1^ | Transition rate into open state | E61 | 2 |
| g | 2.0 | ms^-1^ | Transition rate out of open state | E61 | 2 |
| f' | 0.0 | ms^-1^ | Transition rate into open state, mode Ca | E67 | 2 |
| g' | 0.0 | ms^-1^ | Transition rate out open state, mode Ca | E67 | 2 |
| $\bar{P}_{Ca}$ | 1.24 × 10^-3^ | cm s^-1^ | L-type Ca^2+^ channel permeability to Ca^2+^ | E68 | 3 |
| $\bar{P}_{K}$ | 1.11 × 10^-11^ | cm s^-1^ | L-type Ca^2+^ channel permeability to K^+^ | E71 | 3 |
| $\text{I}_{\text{Ca}_{\text{half}}}$ | -0.4583 | µA µF^-1^ | ICa level that reduces $\bar{P}_{K}$ by half | E71 | 3 |

### S2.9. Ca^2+^ release channel current parameters

| Symbol | Value | Units | Description | Eq. | Ref. |
| --- | --- | --- | --- | --- | --- |
| v_1_ | 3.6 | ms^-1^ | RyR flux channel constant | E79 | 3 |
| n | 4 |  | Cooperativity parameter | E75 | 2 |
| m | 3 |  | Cooperativity parameter | E76 | 2 |
| $k_{a}^{+}$ | 1.215 × 10^10^ | mM^-4^ ms^-1^ | RyR rate constant | E75 | 2 |
| __ | 0.576 | ms^-1^ | RyR rate constant | E75 | 15 |
| $k_{b}^{+}$ | 4.05 × 10^6^ | mM^-3^ ms^-1^ | RyR rate constant | E76 | 2 |
| __ | 1.93 | ms^-1^ | RyR rate constant | E76 | 2 |
| $k_{c}^{+}$ | 0.1 | ms^-1^ | RyR rate constant | E76 | 15 |
| __ | 8.0 × 10^-4^ | ms^-1^ | RyR rate constant | E76 | 2 |

### S2.10. Ca^2+^ transport and buffering parameters

| Symbol | Value | Units | Description | Eq. | Ref. |
| --- | --- | --- | --- | --- | --- |
| ***τ***_tr_ | 0.5747 | ms | Time constant for transfer from subspace to myoplasm | E82 | 16 |
| ***τ***_xfer_ | 9.09 | ms | Time constant for transfer from NSR to JSR | E83 | 3 |
| __ | 2.38 × 10^-3^ | mM | Ca^2+^ half saturation constant for calmodulin | E84 | 3 |
| __ | 0.8 | mM | Ca^2+^ half saturation constant for calsequestrin | E85 | 3 |
| __ | 100 | mM^-1^ ms^-1^ | Ca^2+^ on-rate for troponin high affinity sites | E86 | 3 |
| __ | 3.3 10^-4^ | ms^-1^ | Ca^2+^ off-rate for troponin high affinity sites | E87 | 3 |
| __ | 100 | mM^-1^ ms^-1^ | Ca^2+^ on-rate for troponin low affinity sites | E88 | 3 |
| __ | 4 10^-2^ | ms^-1^ | Ca^2+^ off-rate for troponin low affinity sites | E88 | 2 |
| [HTRPN]_tot_ | 0.14 | mM | Total troponin high-affinity sites | E87 | 2 |
| [LTRPN]_tot_ | 0.07 | mM | Total troponin low-affinity sites | E88 | 2 |
| [CMDN]_tot_ | 5.0 × 10^-2^ | mM | Total myoplasmic calmoduling concentration | E84 | 2 |
| [CSQN]_tot_ | 15 | mM | Total NSR calsequestrin concentration | E85 | 3 |

### S2.11. Force generation parameters

| Symbol | Value | Units | Description | Eq. | Ref. |
| --- | --- | --- | --- | --- | --- |
| __ | 0.04 | ms^-1^ | Transition rate from tropomyosin permissive to non-permissive | E96 | 3 |
| SL | 2.15 | µm | Sarcomere length | E111 | 3 |
| f_XB_ | 0.05 | ms^-1^ | Transition rate from weak to strong cross bridge | E102 | 16 |
| $g_{XB}^{\min}$ | 0.1 | ms^-1^ | Minimum transition rate from strong to weak cross bridge | E105 | 16 |
| ξ | 0.1 | N mm^-2^ | Conversion factor normalizing to physiological force | E120 | 3 |
| $V_{AM}^{\max}$ | 7.2 × 10^-3^ | mM ms^-1^ | Maximal rate of ATP hydrolysis by myofibrils (AM ATPase) | E122 | 6 |
| *$K_{M,AM}^{ATP}$* | 0.03 | mM | ATP half saturation constant of AM ATPase | E122 | 6 |
| *$K_{i,AM}$* | 0.26 | mM | ADP inhibition constant of AM ATPase | E122 | 6 |

### S2.12. Cytoplasmic energy handling parameters

| Symbol | Value | Units | Description | Eq. | Ref. |
| --- | --- | --- | --- | --- | --- |
| C_T_ | 25 | mM | Total concentration of creatine metabolites (both compartments) |  | 17 ,18 |
| $k_{CK}^{cyto}$ | 1.4 × 10^-4^ | ms^-1^ | Forward rate constant of cytoplasmic CK | E140 | 21 |
| $k_{CK}^{mito}$ | 1.33 × 10^-6^ | ms^-1^ | Forward rate constant of mitochondrial CK | E141 | 21 |
| $k_{tr}^{Cr}$ | 2.0 × 10^-3^ | ms^-1^ | Transfer rate constant of CrP | E142 | 21 |
| K_EQ_ | 0.0095 |  | Equilibrium constant of CK | E140 | 17, 16 |
| $V_{ATPase}^{cyto}$ | 1.0 10^-5^ | mM ms^-1^ | Constitutive cytosolic ATP consumption rate | E125 | 19 |

### S2.13. Tricarboxylic acid cycle parameters

| Symbol | Value | Units | Description | Eq. | Ref. |
| --- | --- | --- | --- | --- | --- |
| [AcCoA] | 1.0 | mM | Acetyl CoA concentration | E143 | 4 |
| $k_{cat}^{CS}$ | 0.05 | ms^-1^ | Catalytic constant of CS | E143 | ** |
| $E_{T}^{CS}$ | 0.4 | mM | Concentration of CS | E143 | 4 |
| $K_{M}^{AcCoA}$ | 1.26 × 10^-2^ | mM | Michaelis constant for AcCoA | E143 | 4 |
| $K_{M}^{OAA}$ | 6.4 × 10^-4^ | mM | Michaelis constant for OAA | E143 | 4 |
| C_Kint_ | 1.0 | mM | Sum of TCA cycle intermediates’ concentration | E138 | 4 |
| $k_{f}^{ACO}$ | 1.25 × 10^-2^ | ms^-1^ | Forward rate constant of ACO | E144 | 4 |
| $K_{E}^{ACO}$ | 2.22 |  | Equilibrium constant of ACO | E144 | 4 |
| $K_{ADP}^{a}$ | 0.62 | mM | Activation constant by ADP | E145 | ** |
| $K_{Ca}^{a}$ | 0.0005 | mM | Activation constant for Ca^2+^ | E145 | * |
| $K_{i,NADH}$ | 0.19 | mM | Inhibition constant by NADH | E146 | 4 |
| $k_{cat}^{IDH}$ | 0.03 | ms^-1^ | Rate constant of IDH | E147 | * |
| $E_{T}^{IDH}$ | 0.109 | mM | Concentration of IDH | E147 | 4 |
| [H^+^] | 2.5 × 10^-5^ | mM | Matrix proton concentration | E147 | 4 |
| k_h,1_ | 8.1 × 10^-5^ | mM | Ionization constant of IDH | E147 | 4 |
| k_h,2_ | 5.98 × 10^-5^ | mM | Ionization constant of IDH | E147 | 4 |
| $K_{M}^{ISOC}$ | 1.52 | mM | Michaelis constant for isocitrate | E147 | 4 |
| ni | 2.0 |  | Cooperativity for isocitrate | E147 | 4 |
| $K_{M}^{NAD}$ | 0.923 | mM | Michaelis constant for NAD^+^ | E147 | 4 |
| $K_{D}^{Mg^{2+}}$ | 0.0308 | mM | Activation constant for Mg^2+^ | E148 | 4 |
| $K_{D}^{Ca^{2+}}$ | 1.27 × 10^-3^ | mM | Activation constant for Ca^2+^ | E148 | 4 |
| $E_{T}^{KGDH}$ | 0.5 | mM | Concentration of KGDH | E149 | 4 |
| $k_{cat}^{KGDH}$ | 0.05 | ms^-1^ | Rate constant of KGDH | E149 | ** |
| $K_{M}^{\alpha KG}$ | 1.94 | mM | Michaelis constant for αKG | E149 | 4 |
| $K_{M}^{NAD}$ | 38.7 | mM | Michaelis constant for NAD | E149 | 4 |
| n_αKG_ | 1.2 |  | Hill coefficient of KGDH for αKG | E149 | 4 |
| Mg^2+^ | 0.4 | mM | Mg^2+^ concentration in mitochondria | E148 | 4 |
| $k_{f}^{SL}$ | 5.0 × 10^-4^ | mM^-1^ ms^-1^ | Forward rate constant of SL | E150 | ** |
| $K_{E}^{SL}$ | 3.115 |  | Equilibrium constant of the SL reaction | E150 | 4 |
| [CoA] | 0.02 | mM | Coenzyme A concentration | E150 | 4 |
| $k_{cat}^{SDH}$ | 3.0 × 10^-3^ | ms^-1^ | Rate constant of SDH | E151 | ** |
| $E_{T}^{SDH}$ | 0.5 | mM | SDH enzyme concentration | E151 | 4 |
| $K_{M}^{Suc}$ | 0.03 | mM | Michaelis constant for succinate | E151 | 4 |
| $K_{i}^{FUM}$ | 1.3 | mM | Inhibition constant by fumarate | E151 | 4 |
| $K_{i,sdh}^{OAA}$ | 0.15 | mM | Inhibition constant by oxalacetate | E151 | 4 |
| $k_{f}^{FH}$ | 3.32 × 10^-3^ | ms^-1^ | Forward rate constant for FH | E152 | ** |
| $K_{E}^{FH}$ | 1.0 |  | Equilibrium constant of FH | E152 | 4 |
| k_h1_ | 1.13 × 10^-5^ | mM | Ionization constant of MDH | E153 | 4 |
| k_h2_ | 26.7 | mM | Ionization constant of MDH | E153 | 4 |
| k_h3_ | 6.68 × 10^-9^ | mM | Ionization constant of MDH | E154 | 4 |
| k_h4_ | 5.62 × 10^-6^ | mM | Ionization constant of MDH | E154 | 4 |
| k_offset_ | 3.99 × 10^-2^ |  | pH-independent term in the pH activation factor of MDH | E153 | 4 |
| $k_{cat}^{MDH}$ | 0.111 | ms^-1^ | Rate constant of MDH | E155 | ** |
| $E_{T}^{MDH}$ | 0.154 | mM | Total MDH enzyme concentration | E155 | 4 |
| $K_{M}^{MAL}$ | 1.493 | mM | Michaelis constant for malate | E155 | 4 |
| $K_{i}^{OAA}$ | 3.1 × 10^-3^ | mM | Inhibition constant for oxalacetate | E155 | 4 |
| $K_{M}^{NAD}$ | 0.2244 | mM | Michaelis constant for NAD^+^ | E155 | 4 |
| [GLU] | 10.0 | mM | Glutamate concentration | E156 | 4 |
| $k_{f}^{AAT}$ | 6.44 × 10^-4^ | ms^-1^ | Forward rate constant of AAT | E156 | 4 |
| $K_{E}^{AAT}$ | 6.6 |  | Equilibrium constant of AAT | E156 | 4 |
| k_ASP_ | 1.5 × 10^-6^ | ms^-1^ | Rate constant of aspartate consumption | E156 | ** |

Note: *. The values of the Isocitrate dehydrogenase activation constants by ADP and Ca^2+^ have been modified to reproduce appropriately the kinetics reported by Rutter and Denton (22). **. The kinetic constants of all the TCA cycle enzyme steps have been multiplied by a factor of 1.5-4 with respect to those indicated in g to match the fluxes of the TCA cycle (22) in the integrated model that faces additional restrictions than those of the isolated mitochondrial model.

## S2.14. Oxidative Phosphorylation parameters

| Symbol | Value | Units | Description | Eq. | Ref. |
| --- | --- | --- | --- | --- | --- |
| r_a_ | 6.394 × 10^-13^ | ms^-1^ | Sum of products of rate constants | E157 | 4 |
| r_b_ | 1.762 × 10^-16^ | ms^-1^ | Sum of products of rate constants | E158 | 4 |
| r_c1_ | 2.656 × 10^-22^ | ms^-1^ | Sum of products of rate constants | E157 | 4 |
| r_c2_ | 8.632 × 10^-30^ | ms^-1^ | Sum of products of rate constants | E157 | 4 |
| r_1_ | 2.077 × 10^-18^ |  | Sum of products of rate constants | E157 | 4 |
| r_2_ | 1.728 × 10^-9^ |  | Sum of products of rate constants | E157 | 4 |
| r_3_ | 1.059 × 10^-26^ |  | Sum of products of rate constants | E157 | 4 |
| ρ^res^ | 3.0 × 10^-3^ | mM | Concentration of electron carriers (respiratory complexes I-III-IV) | E157 | 4 |
| K_res_ | 1.35 × 10^18^ |  | Equilibrium constant of respiration | E159 | 4 |
| ρ^res(F)^ | 3.75 × 10^-4^ | mM | Concentration of electron carriers (respiratory complexes II-III-IV) | E161 | * |
| ΔΨ_B_ | 50 | mV | Phase boundary potential | E157 | 4 |
| g | 0.85 |  | Correction factor for voltage | E157 | 4 |
| K_res(F)_ | 5.765 × 10^13^ |  | Equilibrium constant of FADH_2_ oxidation | E162 | 4 |
| $\text{K}_{\text{i}}^{\text{OAA}}$ | 0.15 |  | Inhibition constant for OAA | E162 | 11 |
| p_a_ | 1.656 × 10^-8^ | ms^-1^ | Sum of products of rate constants | E163 | 4 |
| p_b_ | 3.373 × 10^-10^ | ms^-1^ | Sum of products of rate constants | E164 | 4 |
| p_c1_ | 9.651 × 10^-17^ | ms^-1^ | Sum of products of rate constants | E163 | 4 |
| p_c2_ | 4.585 × 10^-17^ | ms^-1^ | Sum of products of rate constants | E163 | 4 |
| p_1_ | 1.346 × 10^-8^ |  | Sum of products of rate constants | E163 | 4 |
| p_2_ | 7.739 × 10^-7^ |  | Sum of products of rate constants | E163 | 4 |
| p_3_ | 6.65 × 10^-15^ |  | Sum of products of rate constants | E163 | 4 |
| ρ^F1^ | 1.5 | mM | Concentration of F_1_F_0_-ATPase | E163 | 4 |
| K_F1_ | 1.71 × 10^6^ |  | Equilibrium constant of ATP hydrolysis | E165 | 4 |
| Pi | 2.0 | mM | Inorganic phosphate concentration | E165 | ** |
| C_A_ | 1.5 | mM | Total sum of mito adenine nucleotides | E129 | ** |
| V_maxANT_ | 0.025 | mM ms^-1^ | Maximal rate of the ANT | E139 | 4 |
| h^ANT^ | 0.5 |  | Fraction of ΔΨ_m_ | E139 | 4 |
| g_H_ | 1.0 × 10^-8^ | mM ms^-1^ mV^-1^ | Ionic conductance of the inner membrane | E166 | 4 |
| ΔpH | -0.6 | pH units | pH gradient across the inner memb. | E167 | 4 |
| C_PN_ | 10.0 | mM | Total sum of mito pyridine nucleotides | E160 | 4 |
| C_mito_ | 1.812 × 10^-3^ | mM mV^-1^ | Inner membrane capacitance | E123 | 4 |

Note: *. The respiratory complex II carriers concentration was adjusted with respect to the value in (4) to match the reported range of oxygen consumption rates (0.04 to 0.5 mM s^-1^ = 5 - 60 µmol O_2_/min/mg) (26). **. The total nucleotide level and inorganic phosphate were corrected with respect to previous publication (4) to follow reported experimental evidence (23, 26)

## S2.15. Mitochondrial Ca^2+^ handling parameters

| Symbol | Value | Units | Description | Eq. | Ref. |
| --- | --- | --- | --- | --- | --- |
| $V_{\max1}^{uni}$ | 7× 10^-3^ | mM ms^-1^ | Vmax uniport Ca^2+^ transport | E168 | * |
| $V_{\max2}^{uni}$ | 9× 10^-5^ | mM ms^-1^ | Vmax uniport Ca^2+^ transport | E168.1 | * |
| ΔΨ° | 91 | mV | Offset membrane potential | E168 | 4 |
| K_act_ | 3.8 × 10^-4^ | mM | Activation constant | E168 | 4 |
| K_trans_ | 0.019 | mM | K_d_ for translocated Ca^2+^ | E168 | 4 |
| L | 110.0 |  | Keq for conformational transitions in uniporter | E168 | 4 |
| n_a_ | 2.8 |  | Uniporter activation cooperativity | E168 | 4 |
|  | 0.8 × 10^-4^ | mM ms^-1^ | Vmax of Na^+^/Ca ^2+^ antiporter | E169 | $ |
| b | 0.5 |  | ΔΨ_m_ dependence of Na^+^/Ca^2+^ antiporter | E169 | 4 |
| K_Na_ | 9.4 | mM | Antiporter Na^+^ constant | E169 | 4 |
| K_Ca_ | 3.75 × 10^-4^ | mM | Antiporter Ca^2+^ constant | E169 | 4 |
| n | 3 |  | Na^+^/Ca^2+^ antiporter cooperativity | E169 | 4 |
| *δ* | 3.0× 10^-4^ |  | Fraction of free [Ca^2+^]_m_ | A93 | 4 |

Note: $. The maximal rate of mNCE was adjusted to meet the transport fluxes experimentally determined (24, 25) in the new integrated model structure in which the Ca^2+^ levels in the cytoplasm are no longer steady as they were in the isolated mitochondrial model (4). The maximal rate of Ca^2+^ uptake from cytosol and microdomain were adjusted accordingly so that the total steady-state mitochondrial Ca^2+^ uptake flux is the same as that in the ECME-RIRR model (27).

### S2.16 Mitochondrial ionic transportation parameters

| Symbol | Value | Units | Description | Eq. | | Ref. |
| --- | --- | --- | --- | --- | --- | --- |
| $k_{1}^{+}$ | 0.0252 | ms^-1^ | NHE forward rate constant | E212 | 1 | |
| $k_{1}^{-}$ | 0.0429 | ms-1 | NHE backward rate constant | E213 | 1 | |
| $k_{4}^{+}$ | 0.16 | ms-1 | NHE forward rate constant | E214 | 1 | |
| $k_{4}^{-}$ | 0.0939 | ms-1 | NHE backward rate constant | E215 | 1 | |
| $K_{Na\_NHE}$ | 24 | mM | Na^+^ Dissociation constant | E212-215 | 1 | |
| $K_{H\_NHE}$ | 1.585×10^-4^ | mM | H^+^ Dissociation constant | E212-215 | 1 | |
| $pK_{i}$ | 8.52 |  | Proton inhibitory constant | E211 | 1 | |
| $n_{i}$ | 3 |  | Hill coefficient for H^+^ binding | E211 | 1 | |
| $c_{NHE}$ | 0.00785 | mM | NHE concentration | E211 | 1 | |
| $K_{H\_KHE}$ | 0.64 | mM | H^+^ Dissociation constant | E216-217 | ** | |
| $K_{K\_KHE}$ | 4×10^-4^ | mM | K^+^ Dissociation constant | E216-217 | ** | |
| $c_{KHE}$ | 0.75 | mM | KHE concentration | E217 | ** | |
| $\delta_{KH}$ | 0.64 |  | KHE backward rate constant | E217 | ** | |
| $K_{Pi,i}$ | 11.06 | mM | Extra-matrix Pi binding constant | E218-219 | 1 | |
| $K_{Pi,m}$ | 11.06 | mM | Mitochondrial matrix Pi binding constant | E218-219 | 1 | |
| $K_{OH,i}$ | 4.08×10^-5^ | mM | Extra-matrix OH- binding constant | E218-219 | 1 | |
| $K_{OH,m}$ | 4.08×10^-5^ | mM | Mitochondrial matrix OH- binding constant | E218-219 | 1 | |
| $V_{PIC,f}$ | 90 | µmol min^-1^ mg protein^-1^ | Forward V_max_ of phosphate carrier | E218 | 1 | |
| $V_{PIC,b}$ | 90 | µmol min^-1^ mg protein^-1^ | Backward V_max_ of phosphate carrier | E218 | 1 | |
| $c_{PiC}$ | 1.6915 | mg protein ml^-1^ | PiC concentration | E218 | 1 | |
| *δ_H_* | 1×10^-4^ |  | Proton buffer capacity | E208 | 1 | |
| *δ_Pi_* | 0.01 |  | P_i_ buffer capacity | E209 | * | |

Note: *. The mitochondrial P_i_ was buffered as [H^+^]_m_ (1) to maintain [P_i_]_m_ level in the new integrated model structure. **. K^+^/H^+^ exchanger parameters were added to balance [H^+^]_m_ at a level shown in (1) .

### S2.17 Antioxidant system parameters

| Symbol | Value | Units | Description | Eq. | Ref. |
| --- | --- | --- | --- | --- | --- |
| $\Phi_{1}$ | 5.0×10^-3^ | mM ms | Constant for GPX activity | E171-172 | 1 |
| $\Phi_{2}$ | 0.75 | mM ms | Constant for GPX activity | E171-172 | 1 |
| $E_{T}^{GPXm}$ | 1.0×10^-5^ | mM | Mitochondrial matrix concentration of GPX | E171 | 1 |
| $E_{T}^{GPXi}$ | 5.0×10^-5^ | mM | Extra-matrix concentration of GPX | E172 | 1 |
| $k_{GR}^{1}$ | 2.5×10^-3^ | ms^-1^ | Catalytic constant of GR | E173-174 | 1 |
| $E_{T}^{GRm}$ | 9.0×10^-4^ | mM | Mitochondrial matrix concentration of GR | E173 | 1 |
| $E_{T}^{GRi}$ | 9.0×10^-4^ | mM | Extra-matrix concentration of GR | E174 | 1 |
| $K_{M}^{NADPH}$ | 0.015 | mM | Michaelis constant for NADPH of GR | E173-174 | 1 |
| $K_{M}^{GSSG}$ | 0.06 | mM | Michaelis constant for GSSG of GR | E173-174 | 1 |
| $[NADPH]_{i}$ | 7.5×10^-2^ | mM | Extra-matrix NADPH concentration | E173-174 | 1 |
| $G_{T}$ | 6 | mM | Total pool of glutathione | E184 | 1 |
| $k_{grx{}_{m}}$ | 1×10^-4^ | mM s^-1^ | Rate constant of mitochondrial matrix  glutaredoxin reaction | E175 | 1 |
| $k_{grx{}_{i}}$ | 1×10^-4^ | mM s^-1^ | Rate constant of extra-matrix glutaredoxin  reaction | E176 | 1 |
| $K_{eq}^{GRX}$ | 1.37×10^-3^ | mM^-1^ | Equilibrium constant of glutaredoxin | E175-176 | 1 |
| $K_{m}^{Grx}$ | 0.01 | mM | Michaelis constant for GSH of GRX | E175-176 | 1 |
| $K_{m}^{PSSG}$ | 0.0005 | mM | Michaelis constant for glutathionylated  protein of glutaredoxin | E175-176 | 1 |
| $k_{PSH}^{1}$ | 0.64 | ms^-1^ | Rate constant of protein glutathionylation | E177-178 | 1 |
| $E_{T}^{PSH}$ | 4×10^-4^ | mM | Concentration of proteins that can become  glutathionylated | E177-178 | 1 |
| $K_{M}^{GSH}$ | 0.75 | mM | Michaelis constant of GSH for  glutathionylation | E177-178 | 1 |
| $K_{act}^{H_{2}O_{2}}$ | 1×10^-3^ | mM | Activation constant of H_2_O_2_ for protein  glutathionylation | E177-178 | 1 |
| $k_{SOD}^{1}$ | 1.2×10^-3^ | mM^-1^ ms^-1^ | Second-order rate constant of SOD | E179-180 | 1 |
| $k_{SOD}^{3}$ | 24 | mM^-1^ ms^-1^ | Second-order rate constant of SOD | E179-180 | 1 |
| $k_{SOD}^{5}$ | 2.4×10^-4^ | ms^-1^ | First-order rate constant of SOD | E179-180 | 1 |
| $K_{i}^{H_{2}O_{2}}$ | 0.5 | mM | Inhibition constant for H_2_O_2_ | E179-180 | 1 |
| $E_{MnSOD}^{T}$ | 1.0×10^-4^ | mM | Mitochondrial concentration of MnSOD | E179 | 1 |
| $E_{CuZnSOD}^{T}$ | 8.6×10^-4^ | mM | Concentration of Cu,ZnSOD | E180 | 1 |
| $E_{T}^{\Pr x3m}$ | 3×10^-3^ | mM | Mitochondrial matrix concentration of Trx  peroxidase (Prx) | E186 | 1 |
| $E_{T}^{\Pr x3i}$ | 1×10^-1^ | mM | Extra-matrix concentration Prx | E187 | 1 |
| $\Phi_{1\Pr x}$ | 3.83 | mM ms | Constant for TxPX activity | E186-187 | 1 |
| $\Phi_{2\Pr x}$ | 1.85 | mM ms | Constant for TxPX activity | E186-187 | 1 |
| $E_{T}^{TrxR2m}$ | 3.5×10^-4^ | mM | Mitochondrial matrix concentration of  TrxR2 | E188 | 1 |
| $E_{T}^{TrxR2i}$ | 3.5×10^-4^ | mM | Extra-matrix concentration of TrxR | E189 | 1 |
| $K_{M}^{TrxSS}$ | 0.035 | mM | Michaelis constant for oxidized Trx  [Trx(SS)] of TrxR | E188-189 | 1 |
| $K_{Mtrx}^{NADPH}$ | 0.012 | mM | Michaelis constant for NADPH of Trx | E188-189 | 1 |
| $k_{TrxR}^{1}$ | 22.7×10^-3^ | ms^-1^ | Rate constant of TrxR | E188-189 | 1 |
| $TrxT_{m}$ | 0.025 | mM | Total pool of mitochondrial matrix  thioredoxin | E190 | 1 |
| $TrxT_{i}$ | 0.05 | mM | Total pool of extra-matrix thioredoxin | E191 | 1 |
| $k_{CAT}^{1}$ | 17 | mM^-1^ ms^-1^ | Rate constant of catalase (CAT) | E192 | 1 |
| $E_{CAT}^{T}$ | 1×10^-6^ | mM | Extra-matrix concentration of CAT | E192 | 1 |
| $fr$ | 5.0×10^-2^ | mM^-1^ | Hydrogen peroxide inhibition factor of CAT | E192 | 1 |
| $C_{NADP_{m}}$ | 0.1 | mM | Sum of NADPH plus NADP^+^ | E193 | 1 |
| $k_{m\_THD}^{NADPH}$ | 0.02 | mM | Michaelis constant for NADPH in  transhydrogenase (THD) | E194-195 | 1 |
| $k_{m\_THD}^{NADHm}$ | 0.01 | mM | Michaelis constantfor NADH in THD | E194-195 | 1 |
| $k_{m\_THD}^{NAD}$ | 0.125 | mM | Michaelis constant for NAD in THD | E194-195 | 1 |
| $k_{m\_THD}^{NADP}$ | 0.017 | mM | Michaelis constant for NADP in THD | E194-195 | 1 |
| $E_{T}^{THD}$ | 1.187×10^-5^ | mM | Concentration of THD enzyme | E195 | 1 |
| $k_{a}^{THD}$ | 1.17474 | ms^-1^ | Forward catalytic constant of THD | E195 | 1 |
| $k_{b}^{THD}$ | 10 | ms^-1^ | Reverse catalytic constant of THD | E195 | 1 |

### S2.18. States variables initial values

| Symbol | Description | Value |
| --- | --- | --- |
| V | Sarcolemmal membrane potential | -84.22 |
| m_Na_ | Sodium channel activation gate | 0.03 |
| n_Na_ | Sodium channel inactivation gate | 0.98 |
| j_Na_ | Sodium channel slow inactivation gate | 0.99 |
| X | Potassium channel activation gate | 1.75 × 10^-4^ |
| [Na^+^]_i_ | Intracellular Na^+^ concentration | 5.03 |
| [Na^+^]_m_ | Mitochondrial Na^+^ concentration | 2.66 |
| [K^+^]_i_ | Intracellular K^+^ concentration | 134.56 |
| [Ca^2+^]_i_ | Intracellular Ca^2+^ concentration | 4.58 × 10^-5^ |
| [Ca^2+^]_NSR_ | Network SR Ca^2+^ concentration | 0.15 |
| [Ca^2+^]_JSR_ | Junctional SR Ca^2+^ concentration | 0.15 |
| [Ca^2+^]_SS_ | Ca^2+^ concentration in the subspace | 4.80×10^-5^ |
| [Ca^2+^]_m_ | Mitochondrial free Ca^2+^ concentration | 9.03 ×10^-4^ |
| [H^+^]_m_ | Mitochondrial H^+^ concentration | 5.51×10^-5^ |
| [P_i_]_m_ | Mitochondrial P_i_ concentration | 4.91 |
| P_C1_ | Fraction of RyR channels in P_C1_ state | 0.93 |
| P_C2_ | Fraction of RyR channels in P_C2_ state | 6.22×10^-2^ |
| P_O2_ | Fraction of RyR channels in P_O2_ state | 1.721 ×10^-11^ |
| C_0_ | L-type Ca^2+^ channel closed – mode normal | 0.99 |
| C_1_ | L-type Ca^2+^ channel closed – mode normal | 1.53 ×10^-5^ |
| C_2_ | L-type Ca^2+^ channel closed – mode normal | 8.88 × 10^-11^ |
| C_3_ | L-type Ca^2+^ channel closed – mode normal | 2.27 × 10^-16^ |
| C_4_ | L-type Ca^2+^ channel closed – mode normal | 2.19 × 10^-22^ |
| O | L-type Ca^2+^ channel open – mode normal | 3.29 × 10^-23^ |
| C_Ca0_ | L-type Ca^2+^ channel closed – mode Ca | 9.04 × 10^-4^ |
| C_Ca1_ | L-type Ca^2+^ channel closed – mode Ca | 5.57 × 10^-8^ |
| C_Ca2_ | L-type Ca^2+^ channel closed – mode Ca | 1.28 × 10^-12^ |
| C_Ca3_ | L-type Ca^2+^ channel closed – mode Ca | 1.32 × 10^-17^ |
| C_Ca4_ | L-type Ca^2+^ channel closed – mode Ca | 5.08 × 10^-23^ |
| O_Ca_ | L-type Ca^2+^ channel open – mode Ca | 2.71 × 10^-27^ |
| y | ICa inactivation gate | 0.98 |
| [LTRPNCa] | Ca^2+^ bound to low affinity troponin sites | 7.20 × 10^-3^ |
| [HTRPNCa] | Ca^2+^ bound to high affinity troponin sites | 0.13 |
| [N_0_] | Nonpermissive tropomyosyn with 0 cross bridges | 0.99 |
| [N_1_] | Nonpermissive tropomyosyn with 1 cross bridges | 8.99× 10^-6^ |
| [P_0_] | Permissive tropomyosyn with 0 cross bridges | 1.04 × 10^-5^ |
| [P_1_] | Permissive tropomyosyn with 1 cross bridges | 9.02 × 10^-6^ |
| [P_2_] | Permissive tropomyosyn with 2 cross bridges | 1.68 × 10^-5^ |
| [P_3_] | Permissive tropomyosyn with 3 cross bridges | 1.46 × 10^-5^ |
| [ATP]_i_ | EC coupling linked ATP concentration | 7.99 |
| [ATP]_ic_ | Cytosolic ATP concentration not linked to EC coupling | 7.99 |
| [CrP]_i_ | Mitochondrial linked creatine phosphate concentration | 23.07 |
| [CrP]_ic_ | Cytosolic creatine phosphate concentration | 23.06 |
| [ADP]_m_ | Mitochondrial ADP concentration | 0.0083 |
| [NADH] | Mitochondrial NADH concentration | 9.04 |
| ΔΨ_m_ | Inner mitochondrial membrane potential | - 186.0 |
| [ISOC] | Isocitrate concentration (mitochondrial) | 0.52 |
| [αKG] | α-ketoglutarate concentration (mitochondrial) | 2.75 × 10^-4^ |
| [SCoA] | Succinyl CoA concentration (mitochondrial) | 0.165 |
| [Suc] | Succinate concentration (mitochondrial) | 4.25 × 10^-4^ |
| [FUM] | Fumarate concentration (mitochondrial) | 0.043 |
| [MAL] | Malate concentration (mitochondrial) | 0.0033 |
| [OAA] | Oxalacetate concentration (mitochondrial) | 1.10 × 10^-7^ |
| [ROS]_i_ | ROS concentration (cytoplasmic) | 3.79 × 10^-9^ |
| [ROS]_m_ | ROS concentration (mitocondrial) | 9.60 × 10^-6^ |
| [H_2_O_2_]_i_ | Hydrogen peroxdize (cytoplasmic) | 4.82 × 10^-8^ |
| [H_2_O_2_]_m_ | Hydrogen peroxdize (mitocondrial) | 2.63 × 10^-4^ |
| [GSH]_i_ | Reduced glutathione (cytoplasmic) | 1.62 |
| [GSH]_m_ | Reduced glutathione (mitocondrial) | 1.65 |
| [GSSG]_m_ | Oxidized glutathione (mitocondrial) | 1.32 |
| [PSSG]_m_ | Mitochondrial matrix glutathionylated proteins | 8.15 × 10^-4^ |
| [PSSG]_i_ | Extra-matrix glutathionylated proteins | 5.60 × 10^-5^ |
| [TrxSH2]_m_ | Extra-matrix reduced thioredoxin | 2.17 × 10^-2^ |
| [TrxSH2]_i_ | Extra-matrix reduced thioredoxin | 4.99 × 10^-2^ |

## References

1. Kembro JM, Aon MA, Winslow RL, O'Rourke B, Cortassa S (2013) Integrating mitochondrial energetics, redox and ROS metabolic networks: a two-compartment model. Biophys J 104(2):332-43.

2. Jafri MS, Rice JJ, and Winslow RL (1998) Cardiac Ca2+ dynamics: the roles of ryanodine receptor adaptation and sarcoplasmic reticulum load. Biophys J 74:1149-1168.

3. Rice JJ, Jafri MS, and Winslow RL (2000) Modeling short-term interval-force relations in cardiac muscle. Am J Physiol Heart Circ Physiol 278:H913-931.

4. Cortassa S, Aon MA, Marban E, Winslow RL, and O'Rourke B (2003) An integrated model of cardiac mitochondrial energy metabolism and calcium dynamics. Biophys J 84:2734-2755.

5. Sakamoto J, and Tonomura Y (1980) Order of release of ADP and Pi from phosphoenzyme with bound ADP of Ca2+-dependent ATPase from sarcoplasmic reticulum and of Na+, K+-dependent ATPase studied by ADP-inhibition patterns. J Biochem (Tokyo) 87:1721-1727.

6. Karatzaferi C, Myburgh KH, Chinn MK, Franks-Skiba K, and Cooke R (2003). Effect of an ADP analog on isometric force and ATPase activity of active muscle fibers. Am J Physiol Cell Physiol 284:C816-825.

7. Luo CH, and Rudy Y (1994) A dynamic model of the cardiac ventricular action potential. I. Simulations of ionic currents and concentration changes. Circ Res 74:1071-1096.

8. Pasa TC, Otero AS, Barrabin H, and Scofano HM (1992) Regulation of the nucleotide dependence of the cardiac sarcolemma Ca(2+)-ATPase. J Mol Cell Cardiol 24:233-242.

9. Chen C, Ko Y, Delannoy M, Ludtke SJ, Chiu W, and Pedersen PL (2004) Mitochondrial ATP synthasome: three-dimensional structure by electron microscopy of the ATP synthase in complex formation with carriers for Pi and ADP/ATP. J Biol Chem 279:31761-31768.

10. Bers DM (2001) Excitation-contraction coupling and cardiac contractile force. Kluwer Academic Publishers, Dordrecht, Boston.

11. Page E (1978) Quantitative ultrastructural analysis in cardiac membrane physiology. Am J Physiol 235:C147-158.

12. Sheu SS, and Fozzard HA (1982) Transmembrane Na+ and Ca2+ electrochemical gradients in cardiac muscle and their relationship to force development. J Gen Physiol 80:325-351.

13. Sheu SS (1989) Cytosolic sodium concentration regulates contractility of cardiac muscle. Basic Res Cardiol 84 Suppl 1:35-45.

14. Elimban V, Zhao D, Dhalla NS (1987) A comparative study of the rat heart sarcolemmal Ca2+-dependent ATPase and myosin ATPase. Mol Cell Biochem 77:143-152.

15. Winslow RL, Rice J, Jafri S, Marban E, O'Rourke B (1999) Mechanisms of altered excitation-contraction coupling in canine tachycardia-induced heart failure, II: model studies. Circ Res 84:571-586.

16. Ruf T, Hebisch S, Gross R, Alpert N, Just H, and Holubarsch C (1996) Modulation of myocardial economy and efficiency in mammalian failing and non-failing myocardium by calcium channel activation and beta-adrenergic stimulation. Cardiovasc Res 32:1047-1055.

17. Selivanov VA, Alekseev AE, Hodgson DM, Dzeja PP, Terzic A (2004) Nucleotide-gated KATP channels integrated with creatine and adenylate kinases: amplification, tuning and sensing of energetic signals in the compartmentalized cellular environment. Mol Cell Biochem 256-257:243-256.

18. Joubert F, Gillet B, Mazet JL, Mateo P, Beloeil J, Hoerter JA (2000). Evidence for myocardial ATP compartmentation from NMR inversion transfer analysis of creatine kinase fluxes. Biophys J 79:1-13.

19. Ingwall JS, Weiss RG (2004) Is the failing heart energy starved? On using chemical energy to support cardiac function. Circ Res 95:135-145.

20. Luo CH, Rudy Y (1994) A dynamic model of the cardiac ventricular action potential. I. Simulations of ionic currents and concentration changes. Circ Res 74:1071-1096.

21. Clark JF, Kuznetsov AV, Radda GK (1997) ADP-regenerating enzyme systems in mitochondria of guinea pig myometrium and heart. Am J Physiol 272:C399-404.

22. Rutter GA, Denton RM (1988) Regulation of NAD+-linked isocitrate dehydrogenase and 2-oxoglutarate dehydrogenase by Ca2+ ions within toluene-permeabilized rat heart mitochondria. Interactions with regulation by adenine nucleotides and NADH/NAD+ ratios. Biochem J 252:181-189.

23. Randle PJ, Tubbs PK (1979) Carbohydrate and fatty acid metabolism. In Handbook of Physiology. Berne RM, Sperelakis N, Geiger R, editors. American Physiological Society, Bethesda. 805-844.

24. Gunter KK, Gunter TE (1994) Transport of calcium by mitochondria. J Bioenerg Biomembr 26:471-485.

25. Gunter TE, Pfeiffer DR (1990) Mechanisms by which mitochondria transport calcium. Am J Physiol 258:C755-786.

26. Balaban RS (2002) Cardiac energy metabolism homeostasis: role of cytosolic calcium. J Mol Cell Cardiol 34:1259-1271.

27. Zhou L, Cortassa S, Wei AC, Aon MA, Winslow RL, et al. (2009) Modeling cardiac action potential shortening driven by oxidative stress-induced mitochondrial oscillations in guinea pig cardiomyocytes. Biophys J 97: 1843-1852.
